# The reduction of astrocytic tau prevents amyloid-β-induced synaptotoxicity

**DOI:** 10.1093/braincomms/fcac235

**Published:** 2022-09-19

**Authors:** Pablo Cisternas, Xavier Taylor, Pablo Martinez, Orlando Maldonado, Nur Jury, Cristian A Lasagna-Reeves

**Affiliations:** Stark Neurosciences Research Institute, Indiana University School of Medicine, Indianapolis, IN 46202, USA; Department of Anatomy, Cell Biology & Physiology, Indiana University School of Medicine, Indianapolis, IN 46202, USA; Stark Neurosciences Research Institute, Indiana University School of Medicine, Indianapolis, IN 46202, USA; Department of Anatomy, Cell Biology & Physiology, Indiana University School of Medicine, Indianapolis, IN 46202, USA; Stark Neurosciences Research Institute, Indiana University School of Medicine, Indianapolis, IN 46202, USA; Department of Anatomy, Cell Biology & Physiology, Indiana University School of Medicine, Indianapolis, IN 46202, USA; Stark Neurosciences Research Institute, Indiana University School of Medicine, Indianapolis, IN 46202, USA; Stark Neurosciences Research Institute, Indiana University School of Medicine, Indianapolis, IN 46202, USA; Department of Anatomy, Cell Biology & Physiology, Indiana University School of Medicine, Indianapolis, IN 46202, USA; Stark Neurosciences Research Institute, Indiana University School of Medicine, Indianapolis, IN 46202, USA; Department of Anatomy, Cell Biology & Physiology, Indiana University School of Medicine, Indianapolis, IN 46202, USA; Center for Computational Biology and Bioinformatics, Indiana University School of Medicine, Indianapolis, IN 46202, USA

**Keywords:** tau, astrocytes, synaptotoxicity, neuroprotection, beta-amyloid

## Abstract

Alzheimer’s disease is a neurological disorder characterized by the overproduction and aggregation of amyloid-beta and the phosphorylation and intraneuronal accumulation of tau. These events promote synaptic dysfunction and loss, leading to neurodegeneration and cognitive deficits. Astrocytes are intimately associated with synapses and become activated under pathological conditions, becoming neurotoxic and detrimentally affecting synapses. Although it has been established that reducing neuronal tau expression prevents amyloid-beta-induced toxicity, the role of astrocytic tau in this setting remains understudied. Herein, we performed a series of astrocytic and neuronal primary cultures to evaluate the effects of decreasing astrocytic tau levels on astrocyte-mediated amyloid-beta-induced synaptic degeneration. Our results suggest that the downregulation of tau in astrocytes mitigates the loss of synapses triggered by their exposure to amyloid-beta. Additionally, the absence of tau from astrocytes promotes the upregulation of several synaptoprotective genes, followed by increased production of the neuroprotective factor Pentraxin 3. These results expand our understanding of the contribution of astrocytic tau to the neurodegenerative process induced by amyloid-beta-stimulation and how reducing astrocytic tau could improve astrocyte function by stimulating the expression of synaptoprotective factors. Reducing endogenous astrocytic tau expression could be a potential strategy to prevent synaptic damage in Alzheimer's disease and other neurological conditions.

## Teaser

Cisternas *et al.* reported how the downregulation of tau in astrocytes mitigates the loss of synapses triggered by their exposure to amyloid-beta. The authors suggested that the absence of tau from astrocytes promotes the upregulation of a series of synaptoprotective genes such as Pentraxin 3.

## Graphical Abstract

**Graphical abstract:**
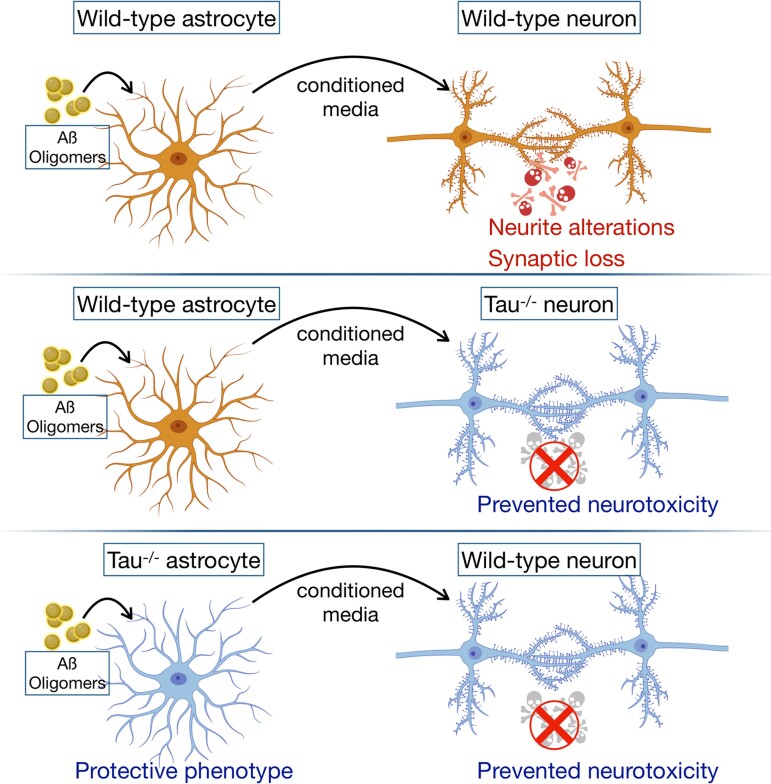

## Introduction

Alzheimer’s disease is a neurological disorder characterized by extracellular plaques composed of aggregated forms of the amyloid-beta (Aβ) peptide and intraneuronal neurofibrillary tangles (NFTs), neuropil threads, and dystrophic neurites that contain aggregated forms of the protein tau (‘tau-pathology’).^1–3^ The pathways underlying tau-pathology-induced synaptotoxicity, neurodegeneration and later cognitive deficits are not fully understood. The prevailing hypothesis is that hyperphosphorylation, misfolding, and fibrillization of tau impairs synaptic structures and triggers neuronal death;^4,5^ it is widely accepted that tau acquires a toxic gain of function. Alternatively, it has recently been suggested that physiological tau functions could possibly contribute to brain disease by allowing other factors and processes to alter other signalling pathways.^6^ For instance, Aβ-mediated neurodegeneration and cognitive dysfunction are considered to depend largely on tau.^6^ It has been reported that reducing tau expression prevents or diminishes Aβ-induced toxicity in cultures of rodent primary neurons.^7,8^ Moreover, *in vivo* studies have revealed that total genetic ablation of endogenous murine tau in a hAβPP mouse model prevents behavioural deficits and synaptic alterations.^7,9–12^ The results of these studies indicate that lowering tau levels is a potential therapeutic strategy for Alzheimer’s disease, bypassing the need to determine which forms of tau are most detrimental.

Under physiological conditions, tau is mainly expressed by neurons.^13^ Therefore, most tau-related studies, including those evaluating the effect of decreasing tau levels, have been focused on neurons.^14,15^ Interestingly, tau is also expressed in astrocytes, although at lower levels than in neurons. Under basal conditions, tau expression is 5 and 10 times lower in astrocytes than in neurons in humans and mice, respectively.^13,16^ Under physiological conditions, several astrocytic mechanisms contribute to the regulation of neuronal function, synaptic integrity and plasticity.^17^ Therefore, alterations in astrocytic function may contribute to synaptic loss in Alzheimer’s disease;^18^ however, the extent of this contribution is currently unclear. As the expression levels of astrocytic tau are low, its relevance has yet to be studied in detail. Recent studies have revealed that lowering tau levels in 4R tau-expressing iPSC-derived astrocytes improves neuronal survival.^19^ Thus, we aimed to determine if the deletion of astrocytic tau protects synapses against the toxic effects of Aβ oligomers.

Our present results suggest that astrocytes could be important mediators of Aβ-induced neurotoxicity and that reducing endogenous astrocytic tau levels ameliorates synaptic loss. Our gene expression analyses suggest that this beneficial effect could partly be due to the upregulation of several astrocytic neuroprotective factors, among which PTX3 showed the most significant increase.

## Materials and methods

### Animals

All work involving mice was performed following the Institutional Animal Care and Use Committee guidelines and in compliance with the Animal Welfare Act, the Guide for the Care and Use of Laboratory Animals, the Office of Laboratory Animal Welfare and the guidelines of Indiana University School of Medicine. Animals were provided with water and food *ad libitum* and housed with light/dark cycles of 12 hrs. P0-P1 wild-type (WT, C57BL/6J, JAX # 000664) and tau knockout (Tau^−/−^, JAX # 007251) male and female pups were used for the cortical astrocyte and neuronal culture experiments, and 6-month-old (6 mo) WT and tau^−/−^ mice were used for the immunofluorescence (IF) studies. Tissues were collected after the animals were euthanized by decapitation under deep anaesthesia. Brains were extracted and prepared as previously described.^20^

### Astrocyte cultures

Astrocytes were cultured as previously described.^21^ Briefly, brains were extracted, and cortices were dissected and washed with calcium-and magnesium-free Hank’s balanced salt solution (CMF-HBSS). Cortices were resuspended in 4.5 ml of CMF-HBSS and incubated with 2.5% trypsin and 1% DNAse for 15 min at 37°C, with gentle swirling of the tubes every 5 min. Next, a cell suspension was obtained by carefully pipetting the tissue. The cell suspension was filtered through a 40 μm pore cell strainer and centrifuged at 1000 rpm for 8 min. The cells were counted, and 1 × 10^6^ cells/ml were seeded in 12 well plates containing 18 mm sterile coverslips in 37°C prewarmed glial medium [minimal essential medium (MEM)], 0.6% glucose, 1 ×  penicillin/streptomycin, and 10% fetal bovine serum (FBS) for the IF experiments. Sixty-millimetre cell culture dishes were used for astrocyte conditioned medium (ACM) collection. The cells were maintained by replacing the glial medium every 2 days until the desired confluency was achieved at 11 days.

### Neuronal cultures

The procedure used for cortical neuronal culture was based on previous work.^22^ Briefly, brains were extracted, and cortices were dissected and washed twice with dissection medium (DM, 97.5% HBSS, 1 ×  sodium pyruvate, 0.1% glucose, 10 mM HEPES). The cortices were suspended in 4.5 ml of DM and incubated with 2.5% trypsin and 1% DNAse for 15 min. After 2 washes with DM, cortices were washed twice with 37°C prewarmed plating media (PM, 86.55% MEM Eagle’s with Earle’s BSS, 10% filtered and heat-inactivated FBS, 0.45% glucose, 1X sodium pyruvate, 1X glutamine, 1X penicillin/streptomycin). Tissue was disaggregated using glass Pasteur pipettes with tips previously rounded by gentle flaming. The resulting suspension was filtered through a cell strainer (40 μm pore). The cells were counted, and 200 000 cells/ml were seeded on 12 well plates containing 1 ml of 37°C prewarmed maintenance medium (MM, 95% neurobasal media, 1 ×  B-27 supplement, 1 ×  glutamine, 1 ×  penicillin/streptomycin) and 18 mm coverslips treated overnight with 0.5% poly-L-lysine in borate buffer. Half of the media was replaced with fresh 37°C prewarmed MM 5 hours after seeding. On the third day of culture, cytosine arabinoside (AraC) was added at a final concentration of 3 μM. Neurons were maintained for 14 DIV. Half of the medium was replaced with fresh 37°C prewarmed MM every 2 days.

### Amyloid-beta oligomer preparation

Aβ oligomers were prepared as previously described.^23^ Briefly, 0.3 mg of lyophilized peptide Aβ_1–42_ (03112 Novex by Life Technologies) was resuspended in 500 μl of 50% acetonitrile/water and relyophilized. The protein powder was dissolved in 200 μl of hexafluoroisopropanol (HFIP), and the suspension was incubated for 15 min at room temperature (RT). Next, 700 μl of cell culture-grade ultrapure water was added, and the suspension was stirred at 500 rpm using a Teflon-coated micro stir bar for 36 h at 22°C in a fume hood. An 18-gauge needle was used to make three holes in the caps of the tubes to allow the evaporation of HFIP.

### Astrocyte stimulation with Aβ, collection of astrocyte conditioned medium and recombinant PTX3 addition

After culturing astrocytes for 11 days *in vitro* (11 DIV), Aβ oligomers were added at a final concentration of 1 μM and the cells were incubated for a further 24 h. This concentration of Aβ, which is higher than physiological levels,^24^ has been previously used in astrocyte culture to trigger reactivity without cellular death^25–27^ The next day, the astrocytes were washed twice with 37°C prewarmed cell culture-grade sterile PBS (1X), and the medium was replaced with glial medium without either phenol red or FBS. The medium was conditioned by astrocytes for 24 h. The next day, the ACM was collected and lyophilized. The protein powder was resuspended in 1.5% of the original lyophilized volume in cell culture-grade sterile PBS (1X). The total protein content of the ACM was determined by the BCA method, and 10 μg of total protein was added to 14 DIV neuronal cultures. The endotoxin content of the ACM was quantified using the Pierce Chromogenic Endotoxin Quantification kit (A39552, Thermo) according to the manufacturer’s instructions. The conditioned media contained under 1 endotoxin unit (EU)/ml, a non-toxic concentration incapable of inducing significant glial activation.^28–31^ Recombinant human PTX3 (R&D Systems 10292-TS-050) was prepared in MM and applied to neuronal cultures at a final concentration of 1 μg/ml as previously described.^32^ Heat-inactivated PTX3 (iPTX3) was obtained by heating aliquots of PTX3 for 10 min at 70°C. Neurons were incubated with the ACM for 24 h, fixed in 37°C prewarmed 4% paraformaldehyde/4% glucose in PBS 1X for 15 min, and stained for synaptic markers.

### AAV production and astrocyte transduction

The mouse tau shRNA sequence (CTGGAGCAGAAATTGTGTATAA) was used for the shRNA experiments. The shRNA-tau sequence was cloned downstream of the GFAP promoter and packaged into an AAV9 vector from Vectorbuilder. A scrambled shRNA sequence (shScr) was used as the transduction control. Primary astrocyte cultures were transduced with 2.44 × 1011 viral particles at 11 DIV for 7 days with the shRNA-tau to downregulate astrocytic tau or shScr.

### Immunofluorescence studies in astrocyte and neuronal cultures

Fixed cells on coverslips were washed with 1X PBS and permeabilized with 0.1% Triton X-100 in 1X PBS for 15 min, followed by a single 5 min wash with 1X PBS. Next, non-specific epitopes were blocked with 3% BSA in 1X PBS for 1 h at RT. Primary antibodies were diluted in 1% BSA in 1X PBS and added to the cells. The cells were then incubated overnight (ON) in a humidified chamber at 4°C. The following antibodies were used for neurons: Synapsin-1 (1:100, ab64581 Abcam) and PSD95 (1:100, ab2723 Abcam). GFAP (1:100, G3893 Sigma) and PTX3 (1:100, PA5-101097 Invitrogen) were used for astrocytes. The next day, the coverslips were washed 3 times with 1X PBS and the corresponding mouse and rabbit Alexa Fluor 488 and 568 secondary antibodies (1:100 Invitrogen A32723 and A11036, respectively) were added. The coverslips were then incubated in 1% BSA for 1 h at RT. Next, three 1X PBS washes were performed, and the coverslips were mounted with Vectashield containing DAPI (Vector Laboratories) and sealed with nail polish. The percentage of GFAP-positive cells was determined to assess astrocyte purity. The coverslips were examined using a Nikon A1R Scanning Confocal Microscope coupled with Nikon NIS Elements imaging software. Synaptic cluster quantification was manually performed with Fiji (ImageJ) as previously described.^33^ Immunofluorescence intensity was manually analyzed with Fiji as previously described.^34^ For each staining procedure, as a negative control, primary antibodies were omitted to determine background and autofluorescence.

### Brain section immunofluorescence

Paraffin sections were deparaffinized in xylene, rehydrated in descending grades of ethanol (EtOH) and washed with deionized water. After blocking with 3% BSA in 1X PBS, the sections were incubated ON at 4°C with GFAP (1:100, G3893 Sigma) and PTX3 (1:100, PA5-101097 Invitrogen) antibodies prepared in 1% BSA 1X PBS. The next day, three 5-minute washes with 1X PBS were performed, followed by incubation for 1 h with the corresponding Alexa Fluor 488 and 568 secondary antibodies in 1% BSA 1X PBS. Finally, brain sections were washed threetimes with 1X PBS, quickly dipped in deionized water and mounted with Vectashield containing DAPI (Vector Laboratories). The sections were examined using the microscope and procedures described for the astrocyte and neuronal cell immunofluorescence experiments. The colocalization analysis was conducted automatically using Fiji with the JACoP plugin; Pearson’s correlation coefficient was used as a colocalization index, as previously described.^35^ For each staining procedure, as a negative control, primary antibodies were omitted to determine background and autofluorescence.

### Western blots

Astrocyte cultures were washed twice with sterile 1X PBS and scraped from the dish with 100 μl RIPA buffer supplemented with one protease inhibitor cocktail tablet/10 ml (Roche 011836170001). The lysate was resuspended thoroughly and centrifuged at 15 000 RPM at 4°C for 10 min. The supernatant was collected, and the protein concentration was determined using the BCA method. The samples were mixed with loading buffer, and 30 μg of total protein was loaded on a NuPAGE 4–12% Bis-Tris protein gel (Invitrogen) and separated at 70 V. The proteins were dry-transferred into a nitrocellulose membrane. The membranes were blocked for 1 h in 5% dry milk powder reconstituted in TBS-T 0.01%; subsequently, total tau (DAKO A0024, 1:000) and β-actin (Santa Cruz sc4778, 1:5000) primary antibodies diluted in TBS-T 0.01% -BSA 3% were added, and the membranes were incubated ON at 4°C. The next day, the antibodies were replaced with the corresponding secondary HRP-conjugated antibodies (goat anti-mouse and goat anti-rabbit Jackson Immunoresearch 711035152 and 715035150, respectively) in 5% milk, and the membranes were incubated for 1 h at RT. According to the manufacturer's specification, the membranes were washed twice with TBS-T and visualized using SuperSignal West Pico PLUS Chemiluminescent (ThermoFisher) developing solution.

### Nanostring gene expression analysis

Total mRNA was purified from primary astrocyte cultures and multiplexed using the nCounter analysis system (NanoString Technologies, Seattle, WA, USA) combined with the nCounter Mouse Glial Profiling Panel. Briefly, 100 ng total RNA per sample was loaded and hybridized with probes for 16 h at 65° C, following the manufacturer’s protocol. Only samples with an RNA integrity number (RIN) > 9 were used for the NanoString analysis. Counts for target genes were normalized to the best fitting housekeeping genes as determined by nSolver software to account for variation in RNA content. The background signal was calculated as the mean value of the negative hybridization control probes. The expression data were excluded when the background signals were lower than the average negative control background signal, and probes with <100 reads for six or more samples were removed from the analysis. Downstream analyses and visualizations of gene expression datasets were performed using the NanoString nCounter Advanced Analysis Report software.

### ELISA assay of ACM

The Abcam Mouse PTX3 ELISA Kit (ab245713) was used to assess the concentration of PTX3 present in the ACM. The 96-well plate was prepared following the manufacturer’s instructions, using 50 μl of undiluted ACM, obtained as described earlier in the Methods. Finally, the plate was read at 405 nm on a microplate reader, and the absorbances on the different samples were analyzed and plotted.

### Cell viability assay

Cell survival was measured using the Cell Titer 96 AQueous One Solution kit according to the manufacturer’s directions (G3582, Promega). Briefly, ninety-six well tissue culture plates were used to seed astrocytes at a density of 10.000 cells per well. At 11 DIV, astrocytes were treated with 1 μM of Aβ oligomers. After 24 h of incubation, the media was replaced with 100 μl of fresh culture medium per well, and 20 μl of Cell Titer 96 AQueous One Solution containing MTS tetrazolium salt was added. MTS is bio-reduced by cells into a coloured formazan product. After 4 h of incubation at 37°C, the absorbance of the wells was measured at 490 nm using a plate reader. The quantity of formazan product is directly proportional to the number of living cells in the culture.

### Data collection and *n* breakdown

**Figure 1:**
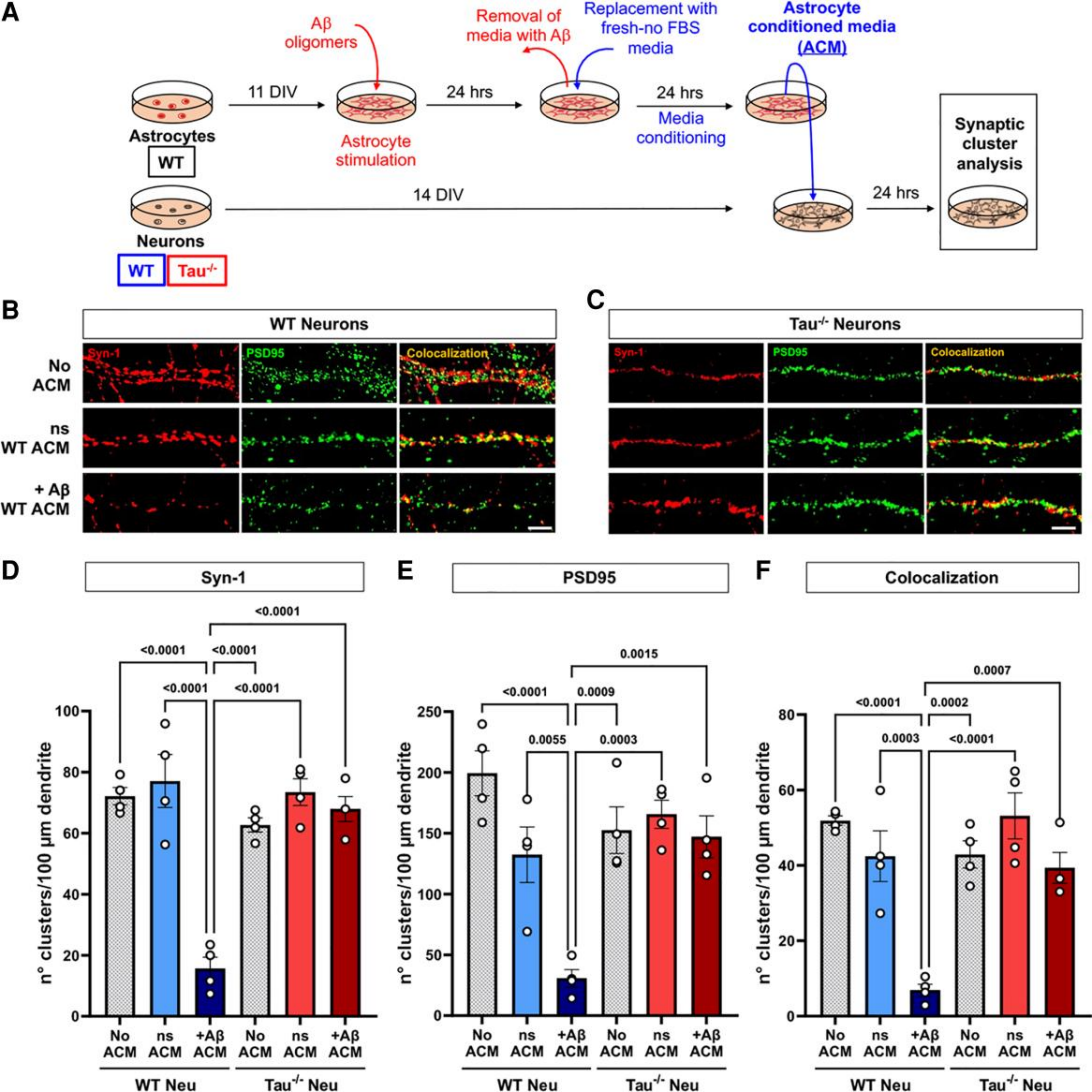
**Neuronal tau ablation prevents synaptic loss in neurons treated with Aβ-stimulated ACM.** (**A**) Schematic representation of the methodology used in the present experiments. (**B and C**) Representative images of 14 DIV WT and tau^−/−^ neurons treated with 10 μg of total protein of ns or Aβ-stimulated (+Aβ) WT ACM. No ACM treatment was used as a control. After 24 h of incubation, the number of Synapsin-1 (**D**, Syn-1), PSD95 (**E**) and synaptic clusters (**F**, colocalization) was quantified. *n* = 4. Shapiro–Wilk normality test, one-way ANOVA test, significance = *P* < 0.05. *P* values indicated on each graph. Data is presented as the mean ± SEM. Bar = 10 μm.

**Figure 2:**
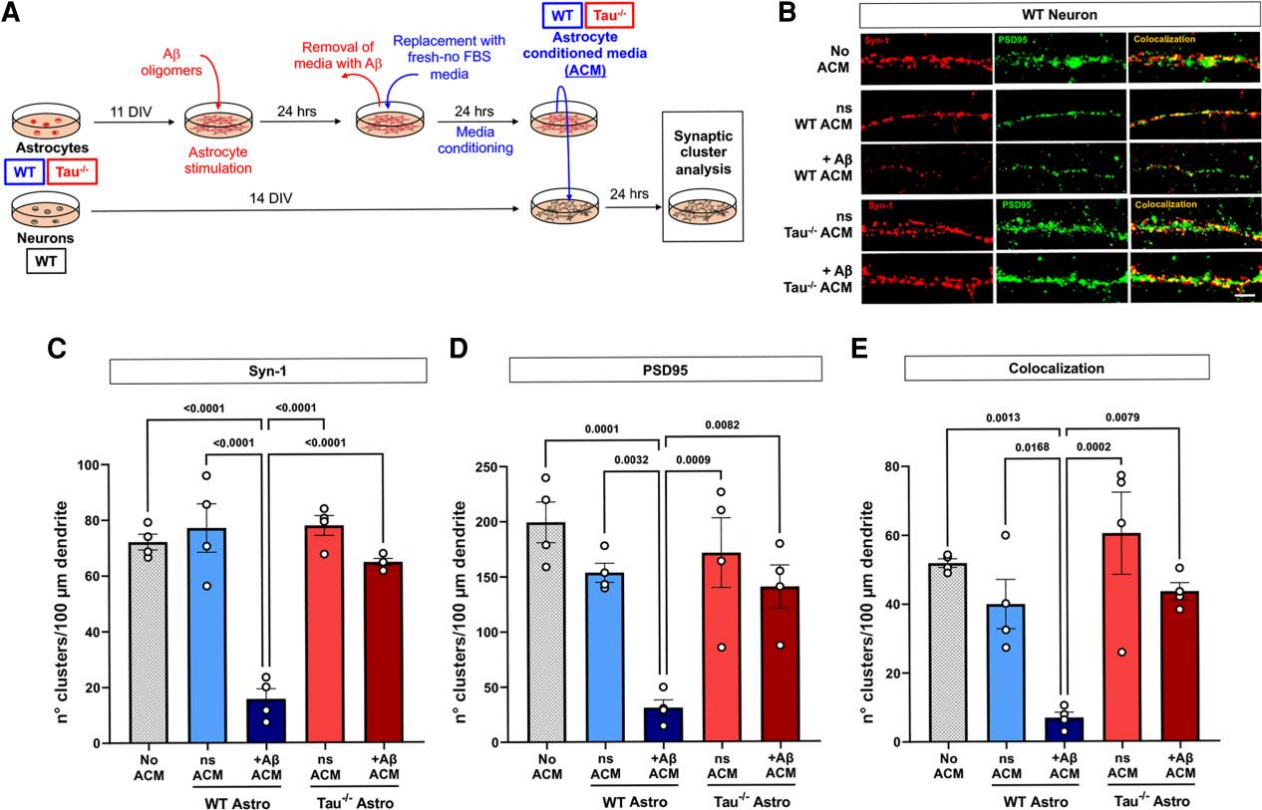
**Astrocytic tau ablation prevents synaptic loss in WT neurons treated with Aβ-stimulated ACM.** (**A**) Schematic representation of the methodology used in the present experiments. (**B**) Representative images of 14 DIV WT neurons treated with 10 μg of total protein from ns or Aβ-stimulated (+Aβ) astrocyte conditioned media (ACM). No ACM treatment was used as a control. After 24 h of incubation, the number of Synapsin-1 (**C**, Syn-1), PSD95 (**D**) and synaptic clusters (**E**, colocalization) were quantified. *n* = 4. Shapiro–Wilk normality test, one-way ANOVA test, significance = *P* < 0.05. *P* values indicated on each graph. Data are presented as the mean ± SEM. Bar = 10 μm.

**Figure 3:**
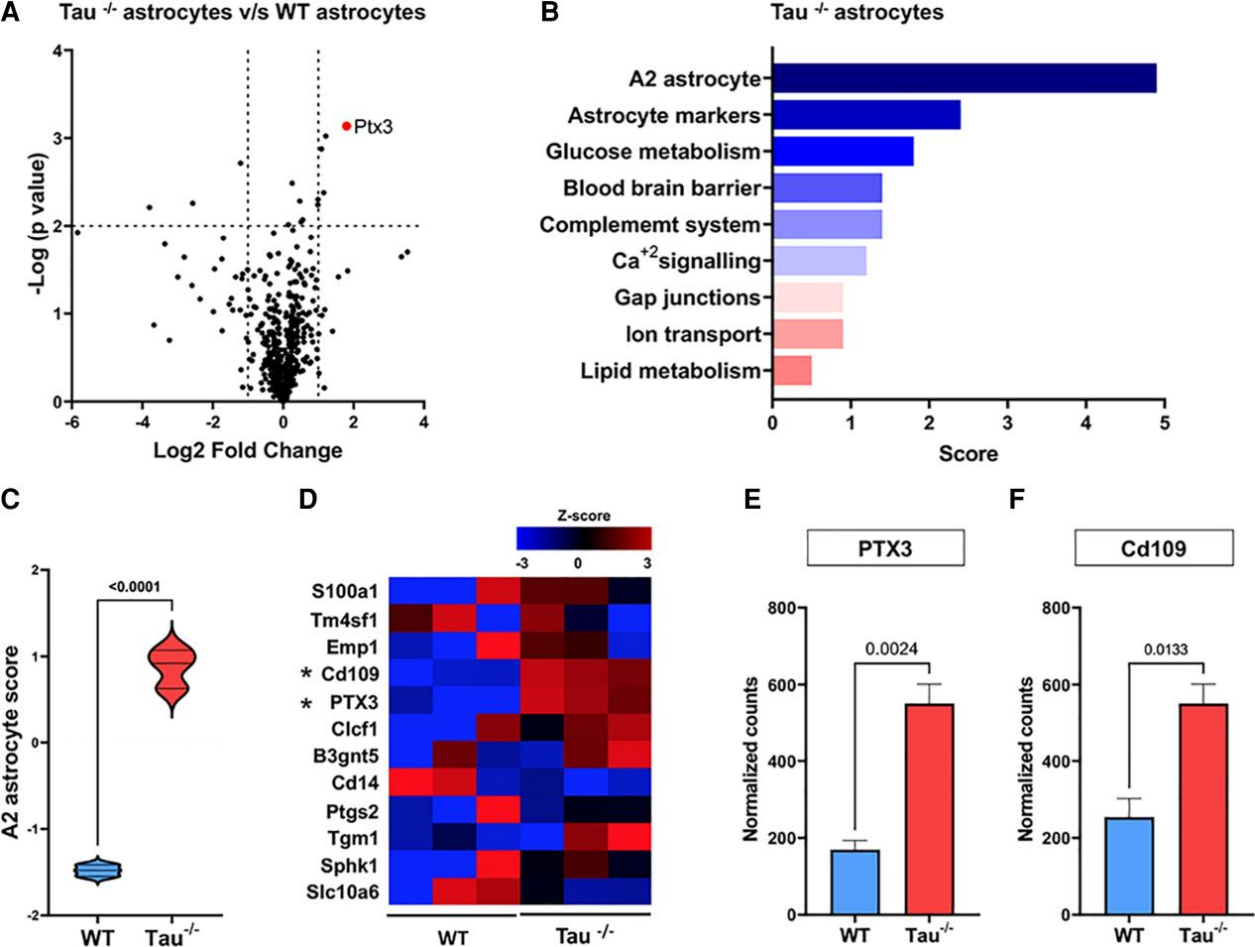
**Tau^−/−^ astrocytes exhibit a protective-type nature.** (**A**) Volcano plot of the differential gene expression between WT and tau^−/−^ astrocytes in the Glial Profiling Panel from Nanostring®. The analysis was performed using nSolver® software. The false discovery rate corrected level of significance is shown as the horizontal line. Significance was set at *P* < 0.05 and log2 Fold Change (vertical lines) at −1 & 1. (**B**) Undirected Global Significance Scores for the top nine gene set annotations provided by nSolver® software from the comparison between WT and tau^−/−^ astrocytes. (**C**) Violin plot representation of the A2 gene set annotation score in WT or tau^−/−^ astrocytes. Shapiro–Wilk normality test, Student’s *t*-test, *P* < 0.05. (**D**) Gene expression heatmap of differentially expressed A2 annotation-related genes between WT and tau^−/−^ astrocytes. (**E**) & (**F**) Normalized counts of PTX3 and Cd109 mRNA. Shapiro–Wilk normality test, Student’s *t*-test, *P* < 0.05. *n* = 3 for all experiments. Data on **C, E** and **F** are presented as the mean ± SEM.

**Figure 4:**
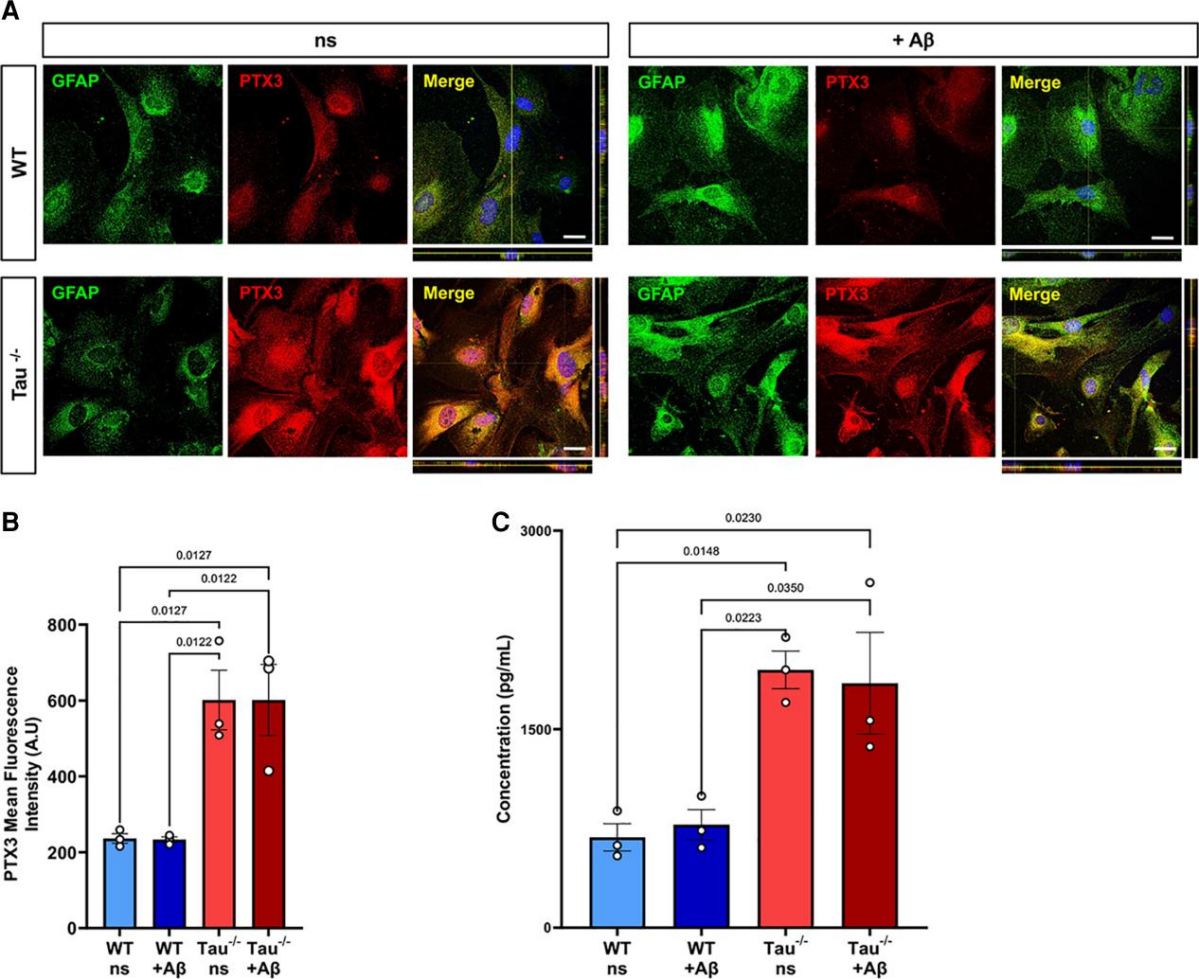
**PTX3 is increased in tau^−/−^ astrocytic culture.** (**A**) Representative images of WT and tau^−/−^ astrocyte cultures treated with 1 μM Aβ oligomers, stained for PTX3 and GFAP. ns astrocytes were used as a control. Bar = 20 μm (**B**) Quantification of the mean fluorescence intensity of PTX3 in the conditions shown in A. *n* = 3, Shapiro–Wilk normality test, one-way ANOVA test, significance = *P* < 0.05. *P* values indicated on the graph. Data are presented as the mean ± SEM. (**C**) PTX3 concentration in astrocyte cultured media of ns or Aβ-stimulated WT and tau^−/−^ astrocyte cultures. *n* = 3 Shapiro–Wilk normality test, one-way ANOVA test, significance = *P* < 0.05. *P* values indicated on the graph. Data are presented as the mean ± SEM.

**Figure 5:**
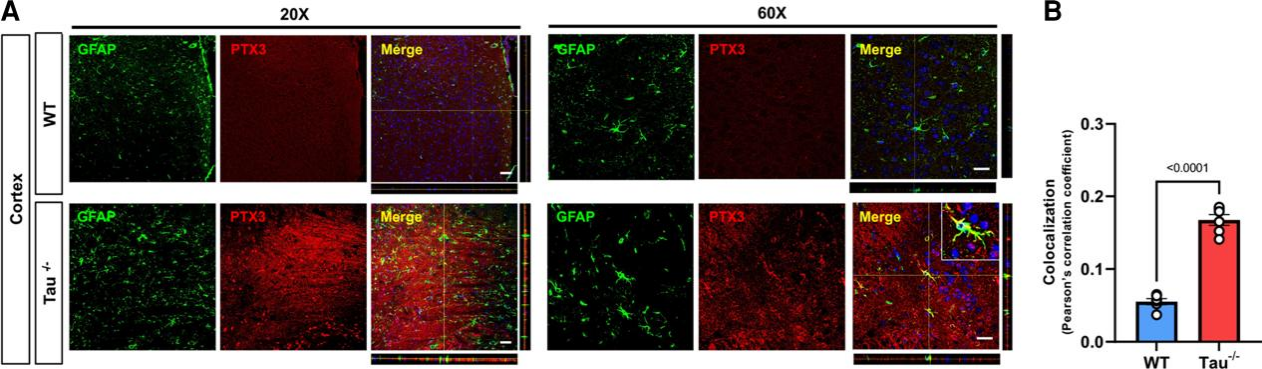
**PTX3 is increased in the tau^−/−^ mouse brain.** (**A**) Representative images of the cortex from 6-month-old WT and tau^−/−^ mice stained for PTX3 and GFAP. Bar = 50 μm on 20X and 20 μm on 60X. (**B**) Quantification of the colocalization index of PTX3 and GFAP. *n* = 6 Shapiro–Wilk normality test, one-way ANOVA test, significance = *P* < 0.05. *P* values indicated on the graph. Data are presented as the mean ± SEM.

**Figure 6:**
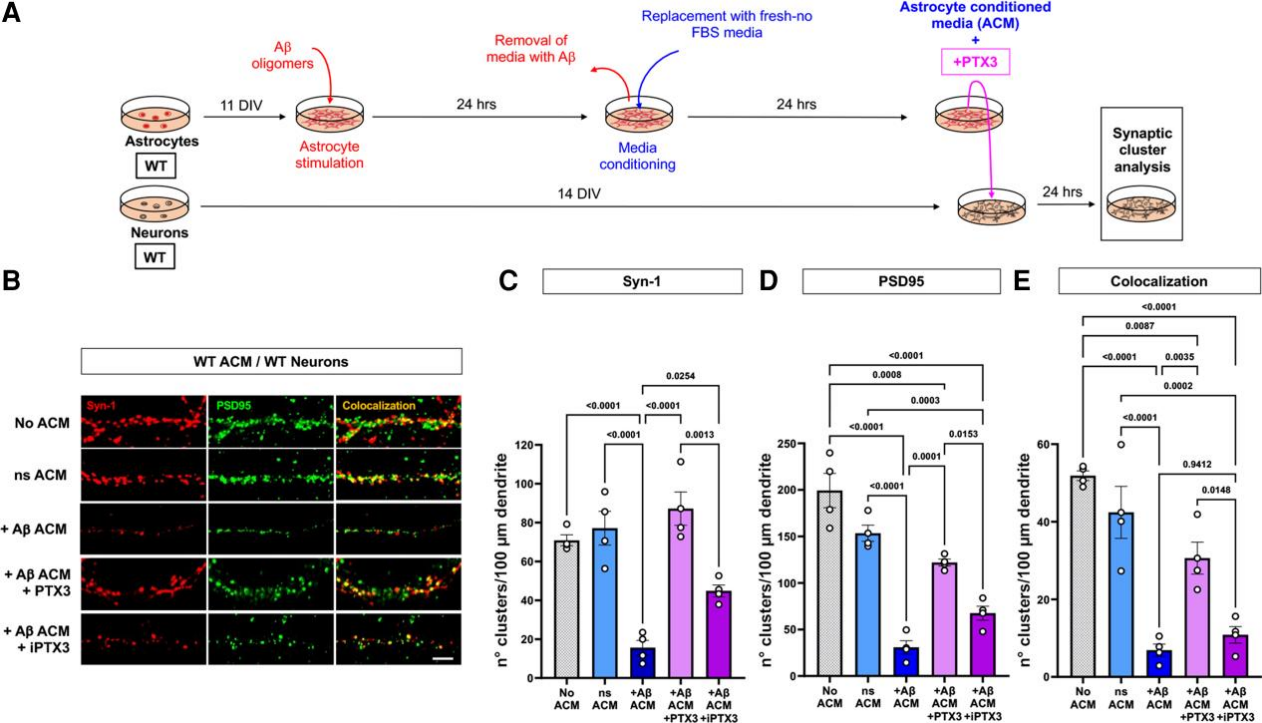
**PTX3 rescues the synaptotoxic effects of Aβ stimulated ACM.** (**A**) Schematic representation of the methodology used in the present experiments. (**B**) Representative images of 14 DIV WT neurons treated with 10 μg of total protein from ns or Aβ-stimulated (+Aβ) ACM with or without 1 μg/ml recombinant PTX3 or heat-inactivated PTX3 (iPTX3). (**C–E**) After 24 h of incubation, the numbers of Synapsin-1, PSD95 and synaptic clusters were quantified. *n* = 4 cultures. Shapiro–Wilk normality test, one-way ANOVA, significance = *P* < 0.05. *P* values shown on each graph. Data is presented as the mean ± SEM. Bar = 10 μm.

**Figure 7:**
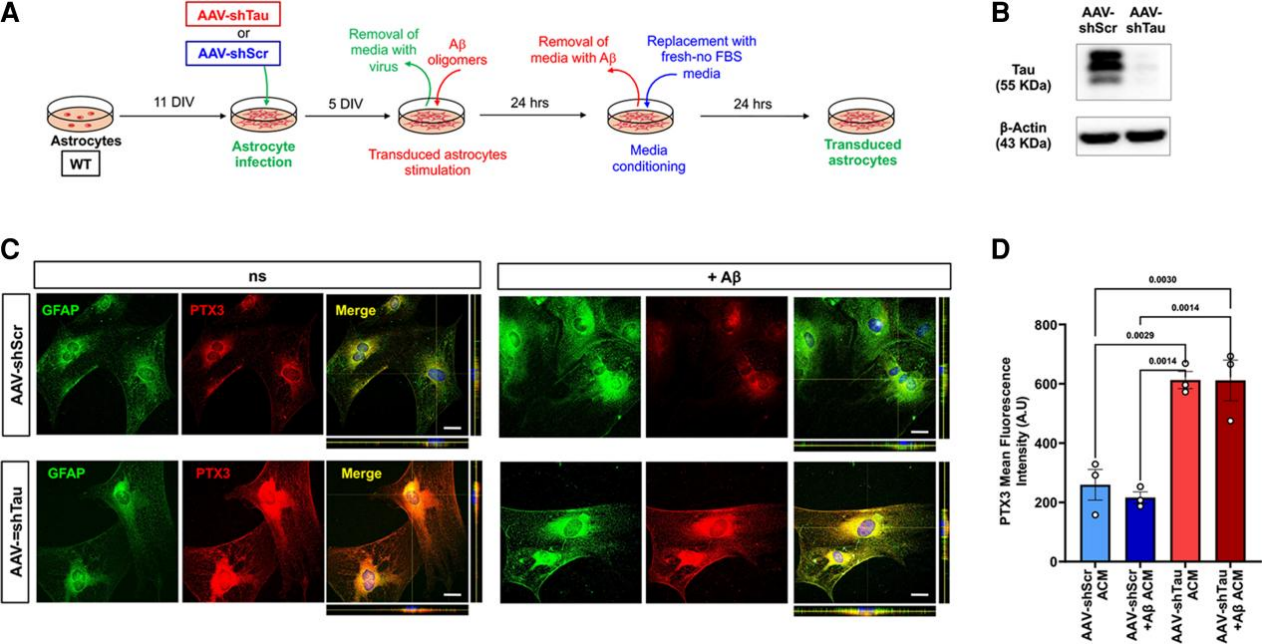
**Astrocytic tau expression silencing via short hairpin increases astrocyte-derived PTX3 levels.** (**A**) Schematic representation of the methodology used in the present experiments. (**B**) Representative western blot for total tau in shScr or shTau transduced astrocytes lysates. β-Actin was used as the loading control. See Supplementary material for uncropped blot. (**C**) Representative images of WT shScr or shTau transduced astrocyte cultures treated with 1 μM Aβ oligomers stained for PTX3 and GFAP. ns astrocytes were used as the control. Bar = 20 μm. (**D**) Quantification of the mean fluorescence intensity of PTX3 on the conditions shown in **C**. All experiments *n* = 3. Shapiro–Wilk normality test, one-way ANOVA test, significance = *P* < 0.05. *P* values indicated on the graph. Data are presented as the mean ± SEM.

**Figure 8:**
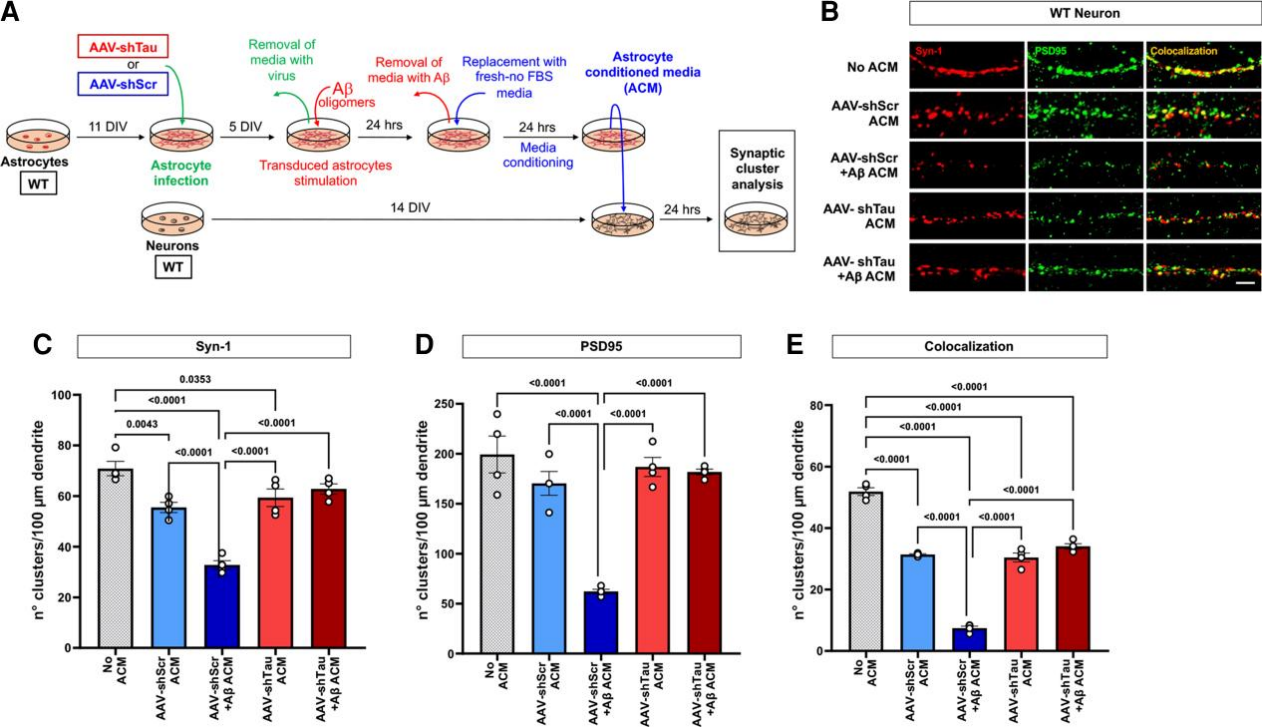
**Astrocytic tau expression silencing via short hairpin RNA prevents synaptic loss in WT neurons treated with Aβ-stimulated ACM.** (**A**) Schematic representation of the methodology used in the present experiments. (**B**) Representative images of 14 DIV WT neurons treated with 10 μg of total protein of AAV-shTau or AAV-shScr non-stimulated (ns) or Aβ-stimulated (+Aβ) WT ACM. Neurons without any type of ACM treatment were used as a control. After 24 h of incubation, the number of Synapsin-1 (**C**, Syn-1), PSD95 (**D**) and synaptic clusters (**E**, colocalization) were quantified. *n* = 4 cultures; 10 neurons were analyzed per culture. Normality was assessed by a Shapiro–Wilk normality test, significance with a one-way ANOVA. Significance = *P* < 0.05. *P* values are shown on each graph. Data are presented as the mean ± SEM. Bar = 10 μm.

The experimental analyses and data collection protocols were performed blind unless otherwise stated. There were no exclusion criteria. The only inclusion criterion was the age of the mice used in this study (P0-P1 and 6 months old). The *n* breakdown for each set of experiments is as follows. *Synaptic quantification* (Figs. 1, 2, 6 and 8): we established four independent neuronal cultures from individual littermates (*n* = 4). Ten photographs of each culture per condition were randomly taken across the coverslip. From each, a single neurite was randomly chosen, analyzed and quantified for synaptic markers. An individual *n* was obtained by averaging the quantifications of each of those 10 neurites. For *tau western blot, tau qPCR* (Supplementary Figure 1A–C)*, differential gene expression analysis* (Fig. 3 and Supplementary Figure 2)*, astrocyte-derived PTX3 concentration* (Fig. 4C)*, AAV-infection and generation of astrocyte conditioned media (ACM)* (Fig. 7), we generated three independent astrocyte cultures for each condition (*n* = 3), established from three independent littermates. For *GFAP/PTX3 co-staining in vitro* (Fig. 4A and B and Fig. 7C and D), we performed three independent astrocyte cultures (*n* = 3). We prepared three technical replicates with each culture. Five pictures per replicate were randomly taken across the coverslip and quantified. We obtained a ‘technical n’ by averaging the data obtained from these five photographs and to obtain the final *n*, we averaged the corresponding three technical *n* values. For *GFAP/PTX3 co-staining in vivo* (Fig. 5), we analyzed the brains of six different mice. A total of four photographs per mouse were taken randomly across the cortices. The final *n* was obtained by averaging the quantification of the colocalization of both markers in those four photos. For the *MTS astrocyte viability assay* (Supplementary Figure 1D), five independent cultures were performed. A total of two technical replicates were performed per culture and averaged to obtain the individual *n*.

### Statistical analysis

Statistical analyses were performed using the GraphPad Prism software. Normal distribution of data was evaluated using a Shapiro–Wilk Normality test; subsequently, statistical comparisons and *P*-value calculations were conducted using one-way ANOVA and two-tailed unpaired Student’s *t*-tests as stated for each experiment. Alternatively, the Kruskal–Wallis test was used when this criterion was not met. Significance was set at *P* < 0.05. Statistically significant *P* values are shown in the figures. Data are presented as the mean ± SEM.

### Data availability

The NanoString data supporting the conclusions of this article are available at NCBI’s Gene Expression Omnibus (GEO) and are accessible via series accession numbers GSE200231. The uncropped version of the western blots shown in this study can be found in Supplementary Figure 3. All other numerical data are available from the corresponding authors upon reasonable request.

## Results

### Decreasing neuronal tau levels mitigate astrocyte-mediated synaptotoxicity induced by Aβ.

It has been previously shown that endogenous tau ablation prevents Aβ toxicity in cultured rodent primary neurons.^36^ Additionally, it has been proposed that astrocytes could be active mediators of Aβ−induced neurotoxicity by exacerbating tau cleavage by caspase-3.^37^ Here, we first aimed to determine if the absence of tau in neurons confers protection against the synaptotoxic effects of Aβ-stimulated astrocytes. We performed a series of astrocytic and neuronal primary cultures in which WT astrocytes were first stimulated with Aβ oligomers. The culture medium was then replaced, and fresh medium was applied and conditioned by the Aβ-stimulated astrocytes. Next, this astrocyte conditioned media (ACM) was used to treat WT or tau^−/−^ neurons (Fig. 1A). As a control, we used ACM from non-stimulated (ns) astrocytes and no ACM treatment. Synaptic integrity was determined by quantifying the number of synaptic clusters of synapsin-1 (Syn-1, presynaptic), PSD95 (postsynaptic) and their level of synaptic colocalization. When tau was absent from neurons, the toxic effects of Aβ-stimulated astrocytes were prevented, as there was no reduction in the cluster number of both Syn-1 and PSD95 and their colocalization on Aβ-ACM-treated tau^−/−^ neurons in relation to control groups (Fig. 1B and C and quantification in D, E and F). These results show that neuronal tau ablation prevents the synaptotoxic effects of Aβ-stimulated astrocytes.

### Decreasing astrocytic tau levels mitigate astrocyte-mediated synaptotoxicity induced by Aβ

It has been recently reported that astrocytic tau is relevant to the process of synaptic loss in several neurological diseases.^17,38^ Therefore, we decided to evaluate the effect of decreasing astrocytic tau levels on astrocyte-mediated synaptotoxicity under Aβ stimulation. The primary culture approach was similar to the method described before; however, WT and tau^−/−^ astrocytes were treated with Aβ oligomers and synaptic integrity in WT neurons was analyzed after treatment with both types of ACM (Fig. 2A). Interestingly, when tau is absent from astrocytes, the synaptotoxic effects of Aβ induction appear to be prevented since Aβ-ACM-treated WT neurons did not show a reduction in the Syn-1 or PSD95 clusters or their colocalization in comparison to the control neurons (Fig. 2B and quantification in C, D and E). We measured tau mRNA and protein levels in WT astrocytes to determine if the neurotoxicity of these astrocytes stimulated with Aβ oligomers was due to astrocytic tau accumulation and aggregation. The Aβ treatment did not affect the levels of tau RNA and tau protein in these WT astrocytes (Supplementary Figure 1A–C). Nevertheless, we cannot rule out that Aβ treatment affected tau phosphorylation or cleavage that is not detected by the total tau antibody utilized in this study. Furthermore, Aβ-treatment did not affect the viability of WT and tau^−/−^ astrocyte cultures (Supplementary Figure 1D), suggesting that the astrocyte-mediated synaptotoxicity under Aβ stimulation could be due to the active secretion of toxic factors rather than to non-specific astrocytic death. These results suggest that astrocytic tau could be involved in synaptic loss under pathological conditions, and its cell-specific deletion confers synaptic protection under Aβ stimulation.

### Tau^−/−^ astrocytes adopt a neuroprotective phenotype

Given the protective effect of astrocytic tau ablation on Aβ-induced synaptotoxicity, we wondered whether there would be heterogeneity in gene expression between WT and tau^−/−^ astrocytes. Thus, we performed a partial transcriptomic analysis using the NanoString Technologies glial profiling panel, which evaluates the expression levels of 770 genes involved in glial cell biology. The volcano plot of the 770 genes analyzed confirmed that tau^−/−^ astrocytes have a distinct gene expression profile compared with WT astrocytes (Fig. 3A and GEO Accession number GSE200231). When the global significance scores of several gene annotations were analyzed, the highest score in tau^−/−^ astrocytes versus WT astrocytes was found in A2-astrocyte annotation, indicative of a neuroprotective astrocytic phenotype^39^ (Fig. 3B–C). Next, we plotted the normalized expression values (*z*-scores) of the genes contained in the A2 annotation on a heat map and compared the two astrocyte phenotypes. Of the 12 A2-neuroprotective genes analyzed, the expression levels of PTX3 and Cd109 were increased in tau^−/−^ astrocytes (Fig. 3D). These results were confirmed by analyzing the normalized counts of both genes (Fig. 3E and F). Importantly, it has become increasingly clear that more complex neuroinflammatory subtypes of astrocytic reactivity exist that do not necessarily align with this recently established A1/A2 dichotomy.^39–41^ Nevertheless, the NanoString glia profiling panel solely offers annotation for these two subtypes.

Interestingly, when we individually analyzed the expression levels of each of the 770 genes, we found that genes known to be positive regulators of synaptic integrity (*Ncam, Nrcam, Uchl1, Fgf2, and Dnm1l*)^42–50^ were upregulated in tau^−/−^ astrocytes versus WT controls (Supplementary Figure 2A and Supplementary Table 1). Consistently, we observed that negative regulators of synaptic integrity (*Ranbp9 and Hdac21*)^51–53^ were downregulated in tau^−/−^ astrocytes versus WT astrocytes (Supplementary Figure 2B and Supplementary Table 1). Taken together, these results show that the absence of astrocytic tau confers a neuroprotective phenotype marked by the increased expression of neuroprotective factors.

### PTX3 expression is upregulated in tau^−/−^ astrocytes and prevents the synaptotoxic effects of Aβ stimulation

PTX3, an acute-phase protein involved in the immune response to inflammation,^54,55^ is a newly discovered marker of anti-inflammatory protective-type astrocytes.^56^ Interestingly, PTX3 is involved in processes promoting synaptic remodelling and neurogenesis,^32,57^ and astrocytic-derived PTX3 preserves blood–brain barrier integrity in pathological conditions.^58^ This evidence, coupled with our findings that PTX3 is highly upregulated in tau^−/−^ astrocytes (Fig. 3A), suggests that PTX3 is an interesting candidate to evaluate in the context of the synaptoprotective effects of astrocytic tau ablation.

We first aimed to determine if PTX3 expression is upregulated in tau^−/−^ astrocytes at the protein level and if Aβ oligomers affect the levels of PTX3 in WT and tau^−/−^ astrocytes. We confirmed that PTX3 expression is upregulated in tau^−/−^ astrocytes by immunofluorescence (Fig. 4A and B). Interestingly, PTX3 levels were not affected by Aβ oligomers in both WT or tau^−/−^ astrocytes, suggesting that even with Aβ stimulation, PTX3 levels in tau^−/−^ astrocytes remain high (Fig. 4A and B). Next, we analyzed the levels of secreted PTX3 in astrocyte supernatants by ELISA and found that PTX3 levels are increased in tau^−/−^ ACM compared with those in WT ACM. The levels of secreted PTX3 in WT and tau^−/−^ ACM were not affected by Aβ treatment (Fig. 4C). To determine if the tau ablation-related increase in PTX3 levels also occurs *in vivo*, we performed double staining for astrocytes (GFAP) and PTX3 on the cortex of brain sections from 6-month-old tau^−/−^ mice and WT controls. We found increased PTX3 expression in cortices of tau^−/−^ mice compared with that in WT mice (Fig. 5). When we specifically quantified the colocalization between PTX3 and GFAP, brains from tau^−/−^ animals exhibited an increased colocalization index, suggesting an increase in PTX3 in tau^−/−^ astrocytes when compared to their WT controls (Fig. 5B). Together, these results suggest that astrocytic tau depletion increases PTX3 expression and secretion.

Next, we repeated our cell culture system approach with modifications to determine if PTX3 and its increase protect tau^−/−^ astrocytes from Aβ toxicity. ACM from Aβ-stimulated WT astrocytes was incubated with recombinant PTX3 and added to WT neurons to determine synaptic integrity. We used heat-inactivated PTX3 (iPTX3) as a control (Fig. 6A). In the presence of PTX3, the toxic effects of Aβ-stimulated astrocytes were avoided. This was demonstrated by a lack of reduction in Syn-1 and PSD95 clusters and a lack of change in their colocalization compared with the control group (Fig. 6B and quantification C–E). These results show that active PTX3 behaves as a synaptoprotective factor and prevents astrocyte-mediated synaptotoxicity induced by Aβ oligomers.

### Astrocytic tau silencing via short hairpin leads to an increase in PTX3 and prevents the synaptotoxic effects of Aβ

To confirm that the increased PTX3 levels and the synaptoprotective effect observed in tau^−/−^ astrocytes were indeed due to a decrease in tau levels and not a gene deletion effect, we evaluated whether silencing tau in WT astrocyte cultures would produce similar results. We transduced WT astrocytes with an AAV expressing a short hairpin for tau (shTau) and subsequently exposed these cells to Aβ oligomers. We used a virus with a scrambled shRNA sequence (shScr) as the control (Fig. 7A). After confirming the decrease in tau levels in astrocytes transduced with the shTau (Fig. 7B), we evaluated the effect of this decrease on PTX3 levels in these astrocyte cultures. As in tau^−/−^ astrocytes, WT astrocytes transduced with shTau showed an increase in the expression of PTX3 independently of the treatment with Aβ (Fig. 7C and D), confirming that the effect on PTX3 levels is due to a decrease in tau. Finally, we evaluated the effect of downregulating astrocytic tau levels on astrocyte-mediated neurotoxicity under Aβ stimulation (Fig. 8A). When tau was downregulated in WT astrocytes transduced with the shTau, the synaptotoxic effects of Aβ induction were prevented; Aβ-shTau ACM-treated WT neurons did not show a reduction in Syn-1 and PSD95 clusters or colocalization in comparison to Aβ-shScr ACM-treated neurons (Fig. 8B and quantification in C–E). These results suggest that the downregulation of tau in astrocytes triggers a neuroprotective genetic profile that mitigates astrocyte-mediated neurotoxicity induced by Aβ.

## Discussion

The data reported here suggest that astrocytic tau is a participating factor necessary for astrocyte-mediated synaptotoxicity induced by Aβ. We also show that astrocytes acquire a synaptoprotective genetic profile when tau levels are downregulated. This protective effect could arise from increased expression and secretion of neuroprotective factors such as PTX3.

Tau-pathology occurs downstream of Aβ accumulation in Alzheimer’s disease.^59–61^ The ablation or reduction of endogenous, nonaggregated WT tau prevents or diminishes Aβ toxicity *in vitro* and *in vivo*. For example, tau reduction prevents behavioural abnormalities in hAPP transgenic mice characterized by the formation of amyloid plaques but not tau aggregates.^7,9,62^ In neuronal cultures, tau ablation prevents axonal transport deficits caused by Aβ oligomers.^14,63^ Interestingly, decreasing endogenous tau levels is beneficial in murine models of autism,^64^ stroke^65^ and epilepsy^9,66,67^ despite the lack of abnormal tau, suggesting that this beneficial effect does not directly involve tau aggregation processes.

Recent studies have revealed that tau reduction could suppress the aberrant neuronal network activities contributed to by nonaggregated tau.^6,15^ Tau reduction was shown to differentially affect functions depending on the neuronal cell type.^15^ This differential effect of tau reduction could result from differences in the biological activities of tau in different cell types. These differences could be mediated by interactions between tau and cell-type-specific molecular pathways. Alternatively, tau ablation in one cell type might indirectly affect another cell type through changes in synaptic network activity. This theory is of substantial interest considering the known ability of astrocytes to interact with neurons at synapses.^68,69^ Interestingly, it has been previously reported that astrocyte-to-neuronal signalling is disrupted in the APP/PS1 mouse model, characterized by the accumulation of Aβ amyloid plaques.^70^ The same group recently demonstrated how the astrocyte-neuronal network interplay is disrupted in the APP/PS1 model.^71^ Specifically, the authors found that astrocytes show Aβ-amyloid density-related hyperactivity that may create a spatial distortion in astrocyte network activity, contributing to cortical neuronal network dysregulation. Therefore, as this study suggests, a loss of astrocyte-mediated regulation is a major contributor to neuronal network pathophysiology in Alzheimer’s disease,^71^ and as total tau reduction beneficially suppresses aberrant neuronal network activities,^6,15^ it is feasible to postulate that the benefit of total tau ablation *in vivo* could arise in part from astrocytic tau downregulation.

Transcriptomic profiling has helped identify the diverse heterogeneity and distinct molecular states of astrocytes in different disease models.^72^ In an early transcriptomic study^73^ and its follow-up,^39^ it was proposed that astrocytes adopt a neurotoxic phenotype after exposure to specific cytokines secreted by microglia exposed to lipopolysaccharide (LPS), whereas they acquire a neuroprotective phenotype after middle cerebral arterial occlusion, a model of ischaemic stroke. Recently, the heterogeneity of neuroinflammatory astrocyte subtypes *in vivo* after LPS challenge has been clarified at single-cell resolution, indicating widespread responses and distinct inflammatory transitions in astrocytes with defined transcriptomic profiles in response to inflammation.^41^ Furthermore, it has become increasingly clear that the binary astrocyte A1 and A2 division is not fixed and that neurotoxic and neuroprotective designations are not all-encompassing.^39–41^ Microglia-induced astrocytes can be identified by their upregulation of complement component 3 (C3) and have been found to lose many normal homeostatic functions, such as promoting neuronal survival, neurite outgrowth, and synapse formation. This suggests that ‘A1’ astrocytes are either unable to maintain synapses or are actively disassemble them by releasing multiple complement components that help drive synaptic degeneration. C3^+^ astrocytes also exert a toxic gain of function by secreting soluble neurotoxin(s) that induce neuronal and oligodendrocyte death, supporting the notion that C3^+^ astrocytes are involved in the development of neurodegenerative diseases.^39,74^ Interestingly, a recently published study demonstrated that the manipulation of tau splicing toward more 4R tau promotes a neurotoxic astrocyte phenotype characterized by the upregulation of C3 levels.^19^ Conversely, under certain stimuli, astrocytes can upregulate the expression of many neurotrophic factors that promote the survival and growth of neurons and thrombospondins, which promote synapse repair.^75^ Thus, subtypes of astrocytes might have beneficial or reparative functions. PTX3, a known marker for neuroprotective astrocytes,^39^ is an acute-phase protein linked to immune responses to inflammation.^56^ PTX3 increases neuronal stem cell proliferation^76^ and promotes synaptogenesis in hippocampal neuronal culture.^32^ PTX3 gene knockout has also been shown to reduce neuronal repair and regeneration following ischaemic brain injury.^58,76,77^ Additionally, PTX3 can bind and tune the complement activation pathway and prevent inflammatory reactions.^77–79^ Specifically, PTX3 regulates C3-deposition by interacting with and recruiting the negative regulator Factor H.^78,79^ This point is of great relevance, taking into consideration novel studies demonstrating that the loss of C3 and C3a receptors, which positively correlated with cognitive decline and Braak staging in human Alzheimer’s disease brains, ameliorates synapse loss and neurodegeneration in Alzheimer’s disease mouse models of amyloidosis and tauopathy.^80,81^ Our data suggest that a mechanism involving PTX3 could be responsible of the synaptoprotective effect of astrocytic tau ablation. In the present study, we demonstrated that the addition of recombinant active PTX3 to cellular media was synaptoprotective (Fig. 6). Nevertheless, the concentration of recombinant active PTX3 was higher than the level of endogenous PTX3 secreted by tau^−/−^ astrocytes (Fig. 4). Therefore, considering that other neuroprotective factors are upregulated in tau^−/−^ astrocytes (Fig. 3 and Supplementary Figure 2), we cannot rule out that other factors could also play a synaptoprotective role in the context of astrocytic tau downregulation.

Currently, we do not have a clear understanding of the mechanism by which tau regulates PTX3 levels in astrocytes. Nevertheless, based on our results, we speculate that the physiological function of tau in astrocytes is associated with the control of a signalling pathway responsible for PTX3 expression and that the downregulation of astrocytic tau levels not only promotes the expression of anti-inflammatory and neuroprotective factors, such as PTX3, but also directly or indirectly inhibits the upregulation of known pro-inflammatory and synaptotoxic related pathways. In conclusion, our study expands our understanding of how reducing tau improves astrocyte function by stimulating synaptoprotective factors. Additionally, we provided evidence for the possible role of astrocytic tau in the known neuronal effects of tau reduction on health and disease. Considering that the current study was performed in astrocytic primary cultures stimulated with a supraphysiological concentration of Aβ, further *in vivo* studies are necessary to validate the observations. Reducing endogenous astrocytic tau expression is a potential strategy for managing the deleterious effects of synaptic damage in Alzheimer’s disease and other neurological conditions.

## Supplementary Material

fcac235_Supplementary_Data

## Abbreviations

Aβ =: amyloid-beta peptide
ACM =: astrocyte conditioned media
AAV =: Adeno-associated viruses
A.U. =: arbitrary units
C3 =: complement component 3
DIV =: days *in vitro*
hAPP =: human amyloid precursor protein
iPTX3 =: inactivated PTX3
mo =: month old
ns =: non-stimulated
PTX3 =: pentraxin-related protein 3
SEM =: standard error of the mean
Syn1 =: Synapsin-1, Tau^−/−^ = tau knock out
WT =: wild-type
