# Supplementary material for: The reduction of astrocytic tau prevents amyloid-β-induced synaptotoxicity
Source: Brain Commun. 2022 Sep 19;4(5):fcac235. doi: 10.1093/braincomms/fcac235 (PMC9527666; doi:10.1093/braincomms/fcac235)
Supplement: fcac235_Supplementary_Data [file fcac235_supplementary_data.zip › Manuscript_original_submission.pdf]

OXFORD UNIVERSITY PRESS | Brain Communications

ASTROCYTIC TAU REDUCTION PREVENTS Aβ-INDUCED SYNAPTOTOXICITY

Journal:	*Brain Communications*
Manuscript ID	BRAINCOM-2022-015
Manuscript Type:	Original Article
Date Submitted by the Author:	11-Jan-2022
Complete List of Authors:	Cisternas, Pablo; Indiana University School of Medicine<br>Taylor, Xavier; Indiana University School of Medicine<br>Martinez, Pablo; Indiana University Purdue University Indianapolis, Department of Anatomy Cell Biology & Physiology<br>Maldonado, Orlando; Indiana University School of Medicine<br>Lasagna-Reeves, Cristian ; Indiana University School of Medicine, Anatomy Cell Biology and Physiology
Keywords:	Tau, Astrocytes, Synaptotoxicity, beta-amyloid, tau reduction

SCHOLARONE™
Manuscripts

ASTROCYTIC TAU REDUCTION PREVENTS Aβ-INDUCED SYNAPTOTOXICITY

Pablo Cisternas[1,2], Xavier Taylor[1,2], Pablo Martinez[1,2], Orlando Maldonado[1], Cristian A. Lasagna-Reeves[1,2,3]*

[1] Stark Neurosciences Research Institute, Indiana University School of Medicine, Indianapolis, IN, USA

[2] Department of Anatomy, Cell Biology & Physiology, Indiana University School of Medicine, Indianapolis, IN, USA

[3]Center for Computational Biology and Bioinformatics, Indiana University School of Medicine, Indianapolis, IN, USA.

***Corresponding author**:

Cristian A. Lasagna-Reeves, Ph.D.

Indiana University School of Medicine

The Stark Neurosciences Research Institute

Neurosciences Research Building 214G

320 West 15th Street

Indianapolis, IN, 46202

Office: (317) 274-7830

Email: clasagna@iu.edu

For Review Only

ABSTRACT

Alzheimer's disease (AD) is a neurological disorder characterized by the overproduction and aggregation of amyloid-beta (Aβ) and phosphorylation and intraneuronal accumulation of tau. These events promote synaptic dysfunction and loss, leading to neurodegeneration and cognitive deficits. Astrocytes are intimately associated with synapses and become activated under pathological conditions, becoming neurotoxic and detrimentally affecting synapses. Although it has been established that reducing neuronal tau expression prevents Aβ induced toxicity, the role of astrocytic tau in this setting remains understudied. Herein, we performed a series of primary culture experiments to evaluate the effect of decreasing astrocytic tau levels on astrocyte-mediated neurotoxicity with Aβ stimulation. Our results suggest that the downregulation of tau in astrocytes promotes a neuroprotective genetic profile that mitigates astrocyte-mediated neurotoxicity induced by Aβ, marked by the increased expression of neuroprotective factors such as Petraxin 3 (PTX3). These results expand our understanding of how reducing tau contributes to improving astrocyte function by stimulating synaptoprotective factors. Additionally, our study clarifies the role of astrocytic tau in known neuronal networks effects of tau reduction on health and disease. Reducing endogenous astrocytic tau expression could be a potential strategy to manage the deleterious effects of synaptic damage in AD and other neurological conditions.

Keywords: Tau, astrocytes, synaptotoxicity, neuroprotection, beta-amyloid.

For Review Only

INTRODUCTION

Alzheimer's disease (AD) is a neurological disorder characterized by extracellular plaques composed of aggregated forms of the amyloid-beta (Aβ) peptide and intraneuronal neurofibrillary tangles (NFTs), neuropil threads, and dystrophic neurites that contain aggregated forms of the protein tau ('tau pathology') [1-3]. The pathways underlying tau-pathology-induced synaptotoxicity, neurodegeneration and later cognitive deficit are not fully understood. The prevailing hypothesis is that hyperphosphorylation, misfolding, and fibrillization of tau impairs synaptic structures and triggers neuronal death [4,5]; it is widely accepted that tau acquires a toxic gain of function. The adverse effects of Aβ on neuronal degeneration and cognitive dysfunction are considered to depend largely on tau [6]. It has been reported that reducing tau expression prevents or diminishes Aβ-induced toxicity in cultures of rodent primary neurons [7,8]. Moreover, *in vivo* studies have revealed that total genetic ablation of endogenous murine tau in a hAβPP mouse model prevents behavioral deficits and synaptic alterations [7,9-12]. The results of these studies indicate that lowering tau levels is a potential therapeutic strategy for AD, bypassing the need to determine which forms of tau are most detrimental.

Under physiological conditions, tau is mainly expressed by neurons [13]. Therefore, most tau-related studies, including those evaluating the effect of decreasing tau levels, have been focused on neurons [14,15]. Interestingly, tau is also expressed in astrocytes, although at lower levels than in neurons [13,16]. Under physiological conditions, several astrocytic mechanisms contribute to the regulation of neuronal function, synaptic integrity and plasticity [17]. Alterations in astrocytic function may therefore contribute to synaptic loss in AD [18]; however, the extent of this contribution is currently unclear. As the expression levels of astrocytic tau in AD and related dementias are low, its relevance has yet to be studied in detail. Recent studies have revealed that tau accumulates

For Review Only

in the astrocytes of AD patients (16,17) and correlates with synaptic alterations; thus, we aimed to determine if astrocytic tau deletion protects synapses against the toxic effects of Aβ oligomers. Here, we determined that astrocytes are important mediators of Aβ-induced neurotoxicity and that reducing endogenous astrocytic tau expression ameliorates synaptotoxicity. Our gene expression analyses suggest that this beneficial effect could be due to the neuroprotective phenotype adopted by *Mapt* null astrocytes as a result of Pentraxin 3 (PTX3) expression upregulation.

MATERIALS AND METHODS

Animals

P0-P1 wild type (WT, C57BL/6J, JAX # 000664) and tau knockout ($Tau^{-/-}$, JAX # 007251) male and female pups were used for the cortical astrocyte and neuronal culture experiments, and 6-month-old (6 mo) WT and $tau^{-/-}$ mice were used for the immunofluorescence studies. Mice were housed at the Indiana University School of Medicine (IUSM) animal care facility and were maintained in accordance with USDA standards (12 h light/dark cycle; food and water *ad libitum*) and Guide for the Care and Use of Laboratory Animals Committee-approved procedures. Tissues were collected after the animals were euthanized by decapitation under deep anesthesia. Brains were extracted and prepared as previously described [19].

Astrocyte cultures

Astrocytes were cultured as previously described [20]. Briefly, brains were extracted and cortexes were dissected and washed with CMF-HBSS. Cortexes were resuspended in 4.5 mL of CMF-HBSS and incubated with 2.5% trypsin and 1% DNAse for 15 min at 37°C, with gentle swirling of the tubes every 5 min. Next, a cell suspension was obtained by carefully pipetting the tissue. The cell suspension was filtered through a 40 µm pore cell strainer and centrifuged at 1000 rpm

for 8 min. The cells were counted, and $1x10^6$ cells/mL were seeded in 12 well plates containing 18 mm sterile coverslips in 37°C prewarmed glial medium (minimal essential medium (MEM)), 0.6% glucose, 1x penicillin/streptomycin, and 10% fetal bovine serum (FBS) for the immunofluorescence experiments. Sixty-millimeter cell culture dishes were used for astrocyte conditioned medium (ACM) collection. The cells were maintained by replacing the glial medium every 2 days until the desired confluency was achieved at 12 days.

Neuronal cultures

The procedure used for cortical neuronal culture was based on previous work [21]. Briefly, brains were extracted and cortexes were dissected and washed twice with dissection medium (DM, 97.5% HBSS, 1X sodium pyruvate, 0.1% glucose, 10 mM HEPES). The cortexes were suspended in 4.5 mL of DM and incubated with 2.5% trypsin and 1% DNAse for 15 min. After 2 washes with DM, cortexes were washed twice with 37°C prewarmed plating media (PM, 86.55% MEM Eagle's with Earle's BSS, 10% filtered and heat-inactivated FBS, 0.45% glucose, 1X sodium pyruvate, 1X glutamine, 1X penicillin/streptomycin). Tissue was desegregated using glass Pasteur pipettes with tips previously rounded by gentle flaming. The resulting suspension was filtered through a cell strainer (40 μm pore). The cells were counted, and 200,000 cells/mL were seeded on 12 well plates containing 1 mL of 37°C prewarmed maintenance medium (MM, 95% neurobasal media, 1x B-27 supplement, 1x glutamine, 1x penicillin/streptomycin) and 18 mm coverslips treated overnight with 0.5% poly-L-lysine in borate buffer. Half of the media was replaced with fresh 37°C prewarmed MM five hours after seeding. On the 3rd day of culture, cytosine arabinoside (AraC) was added at a final concentration of 3 μM. Neurons were maintained for 14 days. Half of the medium was replaced every two days with fresh 37°C prewarmed MM.

Amyloid-beta (Aβ) oligomer preparation

Aβ oligomers were prepared as previously described [22]. Briefly, 0.3 mg of lyophilized peptide $A\beta_{1-42}$ (03112 Novex by Life Technologies) was resuspended in 500 μL of 50% acetonitrile/water and relyophilized. The protein powder was dissolved in 200 μL of hexafluoroisopropanol (HFIP), and the suspension was incubated for 15 min at room temperature. Next, 700 μL of cell-culture-grade ultrapure water was added, and the suspension was stirred at 500 rpm using a Teflon-coated micro stir bar for 36 h at 22°C in a fume hood. An 18-gauge needle was used to make 3 holes in the caps of the tubes to allow the evaporation of HFIP.

Astrocyte stimulation with Aβ, collection of astrocyte-conditioned medium (ACM) and recombinant PTX3 addition

After culturing astrocytes for 12 days *in vitro* (DIV), Aβ oligomers were added at a final concentration of 1 μM and the cells were incubated for a further 24 h. The next day, the astrocytes were washed twice with 37°C prewarmed cell-culture-grade sterile PBS (1X), and the medium was replaced with glial medium without phenol red and FBS. The medium was conditioned for 24 h. The next day, the ACM was collected and lyophilized. The protein powder was resuspended in 1.5% of the original lyophilized volume in cell-culture-grade sterile PBS (1X). The total protein content of the ACM was determined by the BCA method, and 10 μg was added to 14 DIV neuronal cultures. The endotoxin content of the conditioned medium was quantified using the Pierce Chromogenic Endotoxin Quantification kit (A39552, Thermo) according to the manufacturer's instructions. The conditioned media contained under 1 endotoxin unit (EU)/ml, a nontoxic concentration incapable of inducing significant glial activation [23-26]. Recombinant human PTX3 (R&D Systems 10292-TS-050) was prepared in MM at a 1 μg/mL concentration as previously described [27]. Heat-inactivated PTX3 (iPTX3) was obtained by heating aliquots of PTX3 for 10 min at 70 °C. Neurons were

incubated with the ACM for 24 h, fixed in 37°C prewarmed 4% paraformaldehyde 4% glucose in PBS 1X for 15 min, and stained for synaptic markers.

AAV production and astrocyte transduction

The mouse tau shRNA sequence (CTGGAGCAGAAATTGTGTATAA) was used for the shRNA experiments. The shRNA-tau sequence was cloned downstream the GFAP promoter and packaged into an AAV9 vector from Vectorbuilder. A scrambled shRNA sequence (shScr) was used as the transduction control. Primary astrocyte cultures were transduced with $2.44x10^{11}$ viral particles at 12 DIV for 7 days with the shRNA-tau to downregulate astrocytic tau or shScr.

Astrocyte and neuronal culture immunofluorescence (IF)

Fixed cells on coverslips were washed with 1X PBS and permeabilized with 0.1% Triton X-100 in 1X PBS for 15 min, followed by a single 5 min wash with 1X PBS. Next, nonspecific epitopes were blocked with 3% BSA in 1X PBS for 1 h at RT. Primary antibodies were diluted in 1% BSA in 1X PBS and added to the cells. The cells were then incubated overnight (ON) in a humidified chamber at 4 °C. The following antibodies were used for neurons: Synapsin-1 (1:100, ab64581 Abcam) and PSD95 (1:100, ab2723 Abcam). GFAP (1:100, G3893 Sigma) and PTX3 (1:100, PA5-101097 Invitrogen) were used for astrocytes. The next day, the coverslips were washed 3 times with 1X PBS and the corresponding mouse, and rabbit Alexa Fluor 488 and 568 secondary antibodies (Invitrogen A32723 and A11036, respectively) were added. The coverslips were then incubated in 1% BSA for 1 h at RT. Next, three 1X PBS washes were performed and the coverslips were mounted with Vectashield containing DAPI (Vector Laboratories) and sealed with nail polish.

For Review Only

The percentage of GFAP-positive cells was determined to assess astrocyte purity. The coverslips were examined using a Nikon A1R Scanning Confocal Microscope coupled with Nikon NIS Elements imaging software. Synaptic cluster quantification was performed with Fiji (ImageJ) as previously described [28]. Immunofluorescence intensity was analyzed with Fiji as previously described [29].

Brain section immunofluorescence

Paraffin sections were deparaffinized in xylene, rehydrated in ethanol (EtOH) and washed with deionized water. After blocking with 1% BSA in 1X PBS, the sections were incubated ON at 4°C with GFAP (1:100, G3893 Sigma) and PTX3 (1:100, PA5-101097 Invitrogen) antibodies. The next day, a 5 min 1X PBS wash was performed, followed by incubation for 1 h with the corresponding Alexa Fluor 488 and 568 secondary antibodies in 1% BSA. Finally, brain sections were washed 3 times with 1X PBS, quickly dipped in deionized water and mounted with Vectashield containing DAPI (Vector Laboratories). The sections were examined using the microscope and procedures described for the astrocyte and neuronal cell immunofluorescence experiments.

For Review Only

Western blots

Astrocyte cultures were washed twice with sterile 1X PBS and scraped from the dish with 100 μL RIPA buffer supplemented with 1 protease inhibitor cocktail tablet/10 mL (Roche 011836170001). The lysate was resuspended thoroughly and centrifuged at 15,000 RPM at 4℃ for 10 min. The supernatant was collected, and the protein concentration was determined using the BCA method. The samples were mixed with loading buffer, and 30 μg of total protein was loaded on a NuPAGE 4–12% Bis-Tris protein gel (Invitrogen) and separated at 70 V. The proteins were dry-transferred

into a nitrocellulose membrane. The membrane was blocked for 1 h in 5% dry milk powder reconstituted in TBS-T 0.01%; subsequently, total tau (DAKO A0024, 1:5000) and β-actin (Santa Cruz sc4778, 1:5000) primary antibodies diluted in TBS-T 0.01% -BSA 3% were added, and the membrane was incubated ON at 4°C. The next day, the antibodies were replaced with the corresponding secondary HRP-conjugated antibodies (goat anti-mouse and goat anti-rabbit Jackson Immunoresearch 711035152 and 715035150 respectively) in 5% milk, and the membranes were incubated for 1 h. at room temperature (RT). According to the manufacturer's specification, the membranes were washed twice with TBS-T and visualized using SuperSignal West Pico PLUS Chemiluminescent (ThermoFisher) developing solution.

NanoString gene expression analysis

Total mRNA was purified from primary astrocyte cultures and multiplexed using the nCounter analysis system (NanoString Technologies, Seattle, WA, USA) combined with the nCounter Mouse Glial Profiling Panel. Briefly, 100 ng total RNA per sample was loaded and hybridized with probes for 16 h at 65° C following the manufacturer's protocol. Only samples with an RNA integrity number (RIN) > 9 were used for the NanoString analysis. Counts for target genes were normalized to the best fitting housekeeping genes as determined by nSolver software to account for variation in RNA content. The background signal was calculated as the mean value of the negative hybridization control probes. The expression data were excluded when the background signals were lower than the average negative control background signal, and probes with <100 reads for 6 or more samples were removed from the analysis. Downstream analyses and visualizations of gene expression datasets were performed using the NanoString nCounter Advanced Analysis Report software.

ELISA assay on ACM

For Review Only

In order to assess the concentration of PTX3 present on our ACM, the Abcam Mouse PTX3 ELISA Kit (ab245713) was used. The 96-well plate was prepared following the manufacturer's instructions, using 50 µL of undiluted ACM, which was obtained as described before in the Methods. Finally, the plate was read at 405 nm on a microplate reader and the absorbances on the different samples were analyzed and plotted.

Statistical analysis

The experimental analyses and data collection protocols were performed blind unless otherwise stated. Statistical analyses were performed using the GraphPad Prism software. Normal distribution of data was evaluated using a Shapiro–Wilk Normality Test; subsequently, statistical comparisons and p-value calculations were conducted using one-way ANOVA and two-tailed unpaired Student's t-tests as stated for each experiment. Significance was set at $p<0.05$, and significant differences are indicated in the figures. Data are presented as the mean ± SEM. The number of biological replicates is noted in the figures for each experiment.

For Review Only

RESULTS

Decreasing neuronal tau levels mitigate astrocyte-mediated synaptotoxicity induced by Aβ.

Endogenous tau ablation has been shown to prevent Aβ toxicity in cultured rodent primary neurons [30]; additionally, astrocytes are important mediators of Aβ–induced neurotoxicity [31]. Here, we first aimed to determine if neuronal tau absence confers protection against the synaptotoxic effects of Aβ-induced astrocytes. To do so, we performed a series of primary culture experiments in which WT astrocytes were first stimulated with Aβ oligomers. The culture medium was then replaced, and fresh medium was applied and conditioned by the Aβ-stimulated astrocytes. Next, this

astrocyte conditioned media (ACM) was used to treat WT or tau$^{-/-}$ neurons. As a control, we used ACM from nonstimulated (ns) astrocytes. Synaptic integrity was determined by quantifying the number of synaptic clusters of synapsin-1 (Syn-1, presynaptic), PSD95 (postsynaptic) and their level of synaptic colocalization (Figure 1 A). When tau was absent from neurons, the toxic effects of Aβ-stimulated astrocytes were avoided, as there was no reduction in the cluster number of both Syn-1 and PSD95 and their colocalization on Aβ-ACM-treated Tau$^{-/-}$ neurons in relation to control groups (Figure 1 B, C and quantification on D, E & F). These results show that neuronal tau ablation prevents the synaptotoxic effects of Aβ-induced astrocytes.

Decreasing astrocytic tau levels mitigate astrocyte-mediated synaptotoxicity induced by Aβ.

It has been reported recently that astrocytic tau is relevant to the process of synaptic loss in several neurological diseases [17,32]. Therefore, we decided to evaluate the effect of decreasing astrocytic tau levels on astrocyte-mediated neurotoxicity under Aβ stimulation. The primary culture approach was similar, except WT and tau$^{-/-}$ astrocytes were treated with Aβ oligomers followed by an analysis of synaptic integrity in WT neurons after ACM treatment (Figure 2A). Upon exposure to Aβ oligomers, WT astrocytes and tau$^{-/-}$ astrocytes adopted a reactive phenotype, indicated by the increase in the GFAP signal (Supplementary Fig. 1A). Interestingly, when tau is absent from astrocytes, the synaptotoxic effects of Aβ induction was prevented, since Aβ-ACM treated WT neurons did not show a reduction on the clusters of both Syn-1 and PSD95 and their colocalization in comparison to controls (Figure 2 B, C and quantification on D, E & F). We measured tau mRNA and protein levels in these glial cells to determine if the neurotoxicity of WT astrocytes stimulated with Aβ oligomers was due to astrocytic tau accumulation and aggregation. The Aβ treatment did not affect the levels of tau RNA and protein in WT astrocytes

https://mc.manuscriptcentral.com/braincom

(Supplementary Fig. 1B -D). These results suggest that astrocytic tau is necessary to induce synaptic loss under pathological conditions, and its cell-specific deletion confers synaptic protection under Aβ stimulation.

Tau$^{-/-}$ astrocytes adopt a neuroprotective phenotype

Given the protective effect of astrocytic tau ablation on Aβ-induced synaptotoxicity, we wondered whether there would be heterogeneity in gene expression between WT and tau$^{-/-}$ astrocytes. Thus, we performed a partial transcriptomic analysis using the NanoString Technologies glial profiling panel, which evaluates the expression levels of 770 genes involved in glial cell biology. The volcano plot of the 770 genes analyzed confirmed that tau$^{-/-}$ astrocytes have a distinct gene expression profile compared with WT astrocytes (Figure 3A and Supplementary File 1). When the global significance scores of several gene annotations were analyzed, the A2-astrocyte annotation, indicating a neuroprotective astrocytic phenotype [33,34], had the highest score in tau$^{-/-}$ astrocytes compared with WT astrocytes (Figure 3B-C and Supplementary File 1). Next, we plotted the normalized expression values (z-scores) of the genes contained in the A2 annotation on a heat map and compared the two astrocyte phenotypes. Of the 12 A2-neuroprotective genes analyzed, the expression levels of PTX3 and Cd109 were increased in tau$^{-/-}$ astrocytes (Figure 3D). These results were confirmed by analyzing the normalized counts of both genes (Figure 3 E and F). Interestingly, when we individually analyzed the expression levels of each of the 770 genes, we found that genes known to be positive regulators of synaptic integrity (*Ncam, Nrcam, Uchl1, Fgf2 and Dnm1l*) [35-43] were upregulated in tau$^{-/-}$ astrocytes versus WT controls (Supplementary Fig. 2A and Supplementary Table 1). Consistently, we observed that negative regulators of synaptic integrity (*Ranbp9 and Hdac21*) [44-46] were downregulated in tau$^{-/-}$ astrocytes versus WT astrocytes

https://mc.manuscriptcentral.com/braincom

(Supplementary Fig. 2B and Supplementary Table 1). Taken together, these results show that the absence of astrocytic tau confers a neuroprotective phenotype marked by the increased expression of neuroprotective factors.

PTX3 expression is upregulated in tau$^{-/-}$ astrocytes and prevents the synaptotoxic effects of Aβ stimulation

PTX3, an acute-phase protein involved in the immune response to inflammation [47,48], is a newly discovered marker of anti-inflammatory A2 reactive astrocytes [49]. Interestingly, PTX3 is involved in processes promoting synaptic remodeling and neurogenesis [27,50], and astrocytic-derived PTX3 preserves blood-brain barrier integrity in pathological conditions [51]. This evidence, coupled with our findings that PTX3 is highly upregulated in tau$^{-/-}$ astrocytes (Figure 3A), suggests PTX3 is an interesting candidate for evaluation in the context of the synaptoprotective effects of astrocytic tau ablation.

We first aimed to determine if PTX3 expression is upregulated in tau$^{-/-}$ astrocytes at the protein level and if Aβ oligomers affect the levels of PTX3 in WT and tau$^{-/-}$ astrocytes. We confirmed that PTX3 expression is upregulated in tau$^{-/-}$ astrocytes by immunofluorescence. Interestingly, PTX3 levels were not affected by Aβ oligomers in WT or tau$^{-/-}$ astrocytes, suggesting that even with Aβ stimulation, PTX3 levels in tau$^{-/-}$ astrocytes remain high (Figure 4A and B). Next, we analyzed the levels of secreted PTX3 in astrocyte supernatants by ELISA and found that PTX3 levels are increased in tau$^{-/-}$ ACM compared with those in WT ACM. The levels of secreted PTX3 in WT and tau$^{-/-}$ ACM were not affected by Aβ treatment (Figure 4 C). To determine if the tau ablation-related increase in PTX3 levels also occurs *in vivo*, we performed double staining for astrocytes (GFAP) and PTX3 in brain sections from 6-month-old tau$^{-/-}$ mice and WT controls. We found

For Review Only

297 increased PTX3 expression in the hippocampi of $tau^{-/-}$ mice compared to that in WT mice.
298 Interestingly, this increase was mainly observed in astrocytes and their surrounding areas (Figure
299 4 D and quantification on E). These results suggest that astrocytic tau depletion increases PTX3
300 expression and secretion, leading to neuroprotection.

301 We repeated our cell culture system approach to determine whether PTX3 has a direct
302 synaptoprotective effect against astrocyte-mediated synaptotoxicity. ACM from Aβ-stimulated
303 WT astrocytes was incubated with recombinant PTX3 and added to WT neurons to determine
304 synaptic integrity. We used heat-inactivated PTX3 (iPTX3, Figure 5A) as a control. In the presence
305 of PTX3, the toxic effects of Aβ-stimulated astrocytes were avoided. This was demonstrated by a
306 lack of reduction in Syn-1 and PSD95 clusters and a lack of change in their colocalization
307 compared with the control group (Figure 5 B and quantification of C-E). These results show that
308 active PTX3 behaves as a synaptoprotective factor and prevents astrocyte-mediated
309 synaptotoxicity induced by Aβ oligomers.

310

311 **Astrocytic tau silencing via short hairpin leads to an increase in PTX3 and prevents the**
312 **synaptotoxic effects of Aβ**

313 To confirm that the increased PTX3 levels and the neuroprotective effect observed in $tau^{-/-}$
314 astrocytes were indeed due to a decrease in tau levels and not a gene deletion effect, we evaluated
315 whether silencing tau in WT astrocyte cultures would produce similar results. We transduced WT
316 astrocytes with an AAV expressing a short hairpin for tau (shTau) and subsequently exposed these
317 cells to Aβ oligomers. We used a virus with a scrambled shRNA sequence (shScr) as the control
318 (Figure 6 A). After confirming the decrease in tau levels in astrocytes transduced with the shTau
319 (Figure 6 B), we evaluated the effect of this decrease on PTX3 levels. As in $tau^{-/-}$ astrocytes, WT

For Review Only

astrocytes transduced with shTau showed an increase in the expression of PTX3 independently of the treatment with Aβ (Figure 6 C & D), confirming that the effect on PTX3 levels is indeed due to a decrease in tau. Finally, we evaluated the effect of downregulating astrocytic tau levels on astrocyte-mediated neurotoxicity under Aβ stimulation (Figure 7A). When tau was downregulated in WT astrocytes transduced with the shTau, the synaptotoxic effects of Aβ induction was prevented since Aβ-shTau ACM-treated WT neurons did not show a reduction in Syn-1 and PSD95 clusters or colocalization in comparison to Aβ-shScr ACM-treated neurons (Figure 7 B and quantification on C-E). These results suggest that the downregulation of tau in astrocytes triggers a neuroprotective genetic profile that mitigates astrocyte-mediated neurotoxicity induced by Aβ.

DISCUSSION

The data reported here demonstrate that astrocytic tau is necessary for astrocyte-mediated synaptotoxicity induced by Aβ. We also show that astrocytes acquire a neuroprotective profile when tau levels are downregulated. This neuroprotective effect arises from an increase in PTX3 expression and secretion, conferring a synaptoprotective effect under pathological conditions.

Tau pathology occurs downstream of Aβ accumulation in AD [52-54]. The ablation or reduction of endogenous, nonaggregated WT tau prevents or diminishes Aβ toxicity *in vitro* and *in vivo*. For example, tau reduction prevents behavioral abnormalities in hAPP transgenic mice characterized by the formation of amyloid plaques but no tau aggregates [7,9,55]. In neuronal culture, tau ablation prevents axonal transport deficits caused by Aβ oligomers [14,56]. Interestingly, decreasing endogenous tau levels is beneficial in murine models of Parkinson's disease [57], autism [58], stroke [59]

For Review Only

and epilepsy [9,60,61], despite the lack of abnormal tau in these models, suggesting that this beneficial effect does not directly involve tau aggregation processes.

Recent studies have revealed that tau reduction could suppress the aberrant neuronal network activities enabled by nonaggregated tau [6,15]. Tau reduction was shown to differentially affect functions depending on the neuronal cell type [15]. This differential effect of tau reduction could result from differences in the biological activities of tau in different cell types. These differences could be mediated by interactions between tau and cell-type-specific molecular pathways. Alternatively, tau ablation in one cell type might affect another cell type indirectly through changes in synaptic network activity. This theory is of substantial interest considering the results described herein and the known ability of astrocytes to interact with neurons at synapses responding to neurotransmitters with intracellular calcium increases [62] and to release gliotransmitters that regulate neuronal and synaptic activity [63]. Interestingly, it has been previously reported that astrocyte-to-neuronal signaling is disrupted in the APP/PS1 mouse model characterized by the accumulation of Aβ amyloid plaques [64]. The same group recently demonstrated how the astrocyte-neuronal network interplay is disrupted in the APP/PS1 model [65]. Specifically, the authors found that astrocytes show Aβ-amyloid density-related hyperactivity that may create a spatial distortion in astrocyte network activity, contributing to cortical neuronal network dysregulation. Therefore, as this study that suggests that a loss of astrocyte-mediated regulation is a major contributor to neuronal network pathophysiology in AD [65] and as total tau reduction beneficially suppresses aberrant neuronal network activities [6,15], it is feasible to postulate that the benefit of total tau ablation *in vivo* could arise in part from astrocytic tau downregulation.

Recently, transcriptomic profiling has helped identify the diverse heterogeneity and distinct molecular states of astrocytes in different disease models [66]. In an early transcriptomic study [67] and

For Review Only

its follow-up [68], it was proposed that astrocytes adopt an A1 neurotoxic phenotype after exposure to specific cytokines secreted by microglia exposed to lipopolysaccharide (LPS), whereas they acquire an A2 neuroprotective phenotype after middle cerebral arterial occlusion (MCAO), a model of ischemic stroke. However, it has become increasingly clear that more complex neuroinflammatory subtypes of astrocytic reactivity exist that do not necessarily align with the recently established A1/A2 dichotomy [69-71]. A1 astrocytes can be identified by their upregulation of complement component 3 (C3) and have been found to lose many normal homeostatic functions, such as the promotion of neuronal survival, neurite outgrowth, and synapse formation. This suggests that A1s are either unable to maintain synapses or actively disassemble them by releasing multiple complement components that help drive synaptic degeneration. A1 astrocytes also exert a toxic gain of function by secreting soluble neurotoxin(s) that induce neuronal and oligodendrocyte death, supporting the notion that A1 astrocytes are involved in the development of neurodegenerative diseases [68,72]. In contrast, A2 reactive astrocytes upregulate the expression of many neurotrophic factors that promote the survival and growth of neurons, as well as thrombospondins, which promote synapse repair. Thus, A2 astrocytes might have beneficial or reparative functions. PTX3, a newly identified A2-marker, is an acute-phase protein linked to immune responses to inflammation [49]. PTX3 increases neuronal stem cell proliferation [73] and promotes synaptogenesis in hippocampal neuronal culture [27]. PTX3 gene knockout has also been shown to reduce neuronal repair and regeneration following ischemic brain injury [51,73,74]. Additionally, PTX3 can bind and tune the complement activation pathway and prevent inflammatory reactions [74-76]. Specifically, PTX3 regulates C3-deposition by interacting with and recruiting the negative regulator Factor H [75,76]. This point has great relevance, taking into consideration novel studies demonstrating the loss of C3 and C3a receptors, which positively

For Review Only

correlated with cognitive decline and Braak staging in human AD brains, ameliorates synapse loss and neurodegeneration in AD mouse models of amyloidosis and tauopathy [77,78]. Therefore, our results suggest that the downregulation of astrocytic tau levels not only promotes the expression of anti-inflammatory and neuroprotective factors but could also directly or indirectly inhibit the upregulation of known pro-inflammatory and synaptotoxic related pathways.

In conclusion, our study expands our understanding of how reducing tau contributes to improving astrocyte function by stimulating synaptoprotective factors. Additionally, we provided evidences on the role of astrocytic tau in known neuronal networks effects of tau reduction on health and disease. Reducing endogenous astrocytic tau expression is a potential strategy for managing the deleterious effects of synaptic damage in AD and other neurological conditions.

For Review Only

ACKNOWLEDGMENTS AND DISCLOSURES

We thank Dr. Louise Pay for her critical editing of the manuscript. The authors also would like to thank Dr. Juan F. Codocedo and Hernan Brito GD for their input in the study and figure preparation. This work was supported by the Alzheimer's Association grants AARFD-643712 & AARG-D591887, the NIH grants NIH/NIA: (1R01AG059639), NIH/NINDS: (1R01NS119280), and the Department of Defense grant AZ180006.

CONFLICTS OF INTEREST

The authors declare no conflicts of interest.

AUTHORS CONTRIBUTIONS

CAL-R and PC conceived and coordinated the study; PC performed the cell culture experiments, immunofluorescence experiments and biochemical assays. XT performed the NanoString analysis and generated data with nSolver. PM designed the shTau and shScr-containing AAV and assisted with experimental design. OM supported the generation of data from neuronal cultures. PC analyzed the data and drafted the images for publication. CAL-R and PC wrote the manuscript. All authors have read and approved the final manuscript.

AVAILABILITY OF DATA

The data that supports the findings of this study are available in the supplementary material of this article.

For Review Only

REFERENCES

1 Holtzman, D. M., Morris, J. C. & Goate, A. M. Alzheimer's disease: the challenge of the second century. *Sci Transl Med* **3**, 77sr71, doi:10.1126/scitranslmed.3002369 (2011).

2 Castellani, R. J., Rolston, R. K. & Smith, M. A. Alzheimer disease. *Dis Mon* **56**, 484-546, doi:10.1016/j.disamonth.2010.06.001 (2010).

3 Serrano-Pozo, A., Frosch, M. P., Masliah, E. & Hyman, B. T. Neuropathological alterations in Alzheimer disease. *Cold Spring Harb Perspect Med* **1**, a006189, doi:10.1101/cshperspect.a006189 (2011).

4 Wang, Y. & Mandelkow, E. Tau in physiology and pathology. *Nat Rev Neurosci* **17**, 5-21, doi:10.1038/nrn.2015.1 (2016).

5 Spires-Jones, T. L. & Hyman, B. T. The intersection of amyloid beta and tau at synapses in Alzheimer's disease. *Neuron* **82**, 756-771, doi:10.1016/j.neuron.2014.05.004 (2014).

6 Chang, C. W., Shao, E. & Mucke, L. Tau: Enabler of diverse brain disorders and target of rapidly evolving therapeutic strategies. *Science* **371**, doi:10.1126/science.abb8255 (2021).

7 Roberson, E. D. *et al.* Amyloid-beta/Fyn-induced synaptic, network, and cognitive impairments depend on tau levels in multiple mouse models of Alzheimer's disease. *The Journal of neuroscience : the official journal of the Society for Neuroscience* **31**, 700-711, doi:10.1523/JNEUROSCI.4152-10.2011 (2011).

8 DeVos, S. L. *et al.* Synaptic Tau Seeding Precedes Tau Pathology in Human Alzheimer's Disease Brain. *Front Neurosci* **12**, 267, doi:10.3389/fnins.2018.00267 (2018).

9 Roberson, E. D. *et al.* Reducing endogenous tau ameliorates amyloid beta-induced deficits in an Alzheimer's disease mouse model. *Science* **316**, 750-754, doi:10.1126/science.1141736 (2007).

10 Cantero, J. L. *et al.* Role of tau protein on neocortical and hippocampal oscillatory patterns. *Hippocampus* **21**, 827-834, doi:10.1002/hipo.20798 (2011).

11 Ittner, L. M. *et al.* Dendritic function of tau mediates amyloid-beta toxicity in Alzheimer's disease mouse models. *Cell* **142**, 387-397, doi:10.1016/j.cell.2010.06.036 (2010).

12 Hall, A. M. *et al.* Tau-dependent Kv4.2 depletion and dendritic hyperexcitability in a mouse model of Alzheimer's disease. *J Neurosci* **35**, 6221-6230, doi:10.1523/JNEUROSCI.2552-14.2015 (2015).

13 Zhang, Y. *et al.* An RNA-sequencing transcriptome and splicing database of glia, neurons, and vascular cells of the cerebral cortex. *The Journal of neuroscience : the official journal of the Society for Neuroscience* **34**, 11929-11947, doi:10.1523/JNEUROSCI.1860-14.2014 (2014).

14 Vossel, K. A. *et al.* Tau reduction prevents Abeta-induced axonal transport deficits by blocking activation of GSK3beta. *J Cell Biol* **209**, 419-433, doi:10.1083/jcb.201407065 (2015).

15 Chang, C. W., Evans, M. D., Yu, X., Yu, G. Q. & Mucke, L. Tau reduction affects excitatory and inhibitory neurons differently, reduces excitation/inhibition ratios, and counteracts network hypersynchrony. *Cell Rep* **37**, 109855, doi:10.1016/j.celrep.2021.109855 (2021).

16 Zhang, T. *et al.* Purification and characterization of a novel phloretin-2'-O-glycosyltransferase favoring phloridzin biosynthesis. *Sci Rep* **6**, 35274, doi:10.1038/srep35274 (2016).

17 Richetin, K. *et al.* Tau accumulation in astrocytes of the dentate gyrus induces neuronal dysfunction and memory deficits in Alzheimer's disease. *Nat Neurosci* **23**, 1567-1579, doi:10.1038/s41593-020-00728-x (2020).

For Review Only

18 Henstridge, C. M., Hyman, B. T. & Spires-Jones, T. L. Beyond the neuron-cellular interactions early in Alzheimer disease pathogenesis. *Nat Rev Neurosci* **20**, 94-108, doi:10.1038/s41583-018-0113-1 (2019).

19 You, Y. *et al.* Tau as a mediator of neurotoxicity associated to cerebral amyloid angiopathy. *Acta Neuropathol Commun* **7**, 26, doi:10.1186/s40478-019-0680-z (2019).

20 Kaech, S. & Banker, G. Culturing hippocampal neurons. *Nature protocols* **1**, 2406-2415, doi:10.1038/nprot.2006.356 (2006).

21 Beaudoin, G. M., 3rd *et al.* Culturing pyramidal neurons from the early postnatal mouse hippocampus and cortex. *Nature protocols* **7**, 1741-1754, doi:10.1038/nprot.2012.099 (2012).

22 Guerrero-Munoz, M. J. *et al.* Amyloid-beta oligomers as a template for secondary amyloidosis in Alzheimer's disease. *Neurobiol Dis* **71**, 14-23, doi:10.1016/j.nbd.2014.08.008 (2014).

23 Gao, H. M., Hong, J. S., Zhang, W. & Liu, B. Synergistic dopaminergic neurotoxicity of the pesticide rotenone and inflammogen lipopolysaccharide: relevance to the etiology of Parkinson's disease. *J Neurosci* **23**, 1228-1236 (2003).

24 Lee, E. J. *et al.* Alpha-synuclein activates microglia by inducing the expressions of matrix metalloproteinases and the subsequent activation of protease-activated receptor-1. *J Immunol* **185**, 615-623, doi:10.4049/jimmunol.0903480 (2010).

25 Park, J. Y., Paik, S. R., Jou, I. & Park, S. M. Microglial phagocytosis is enhanced by monomeric alpha-synuclein, not aggregated alpha-synuclein: implications for Parkinson's disease. *Glia* **56**, 1215-1223, doi:10.1002/glia.20691 (2008).

26 Zhang, W. *et al.* Aggregated alpha-synuclein activates microglia: a process leading to disease progression in Parkinson's disease. *FASEB J* **19**, 533-542, doi:10.1096/fj.04-2751com (2005).

27 Fossati, G. *et al.* Pentraxin 3 regulates synaptic function by inducing AMPA receptor clustering via ECM remodeling and beta1-integrin. *EMBO J* **38**, doi:10.15252/embj.201899529 (2019).

28 Cisternas, P. *et al.* Gestational Hypothyroxinemia Affects Glutamatergic Synaptic Protein Distribution and Neuronal Plasticity Through Neuron-Astrocyte Interplay. *Mol Neurobiol*, doi:10.1007/s12035-015-9609-0

10.1007/s12035-015-9609-0 [pii] (2015).

29 Shihan, M. H., Novo, S. G., Le Marchand, S. J., Wang, Y. & Duncan, M. K. A simple method for quantitating confocal fluorescent images. *Biochem Biophys Rep* **25**, 100916, doi:10.1016/j.bbrep.2021.100916 (2021).

30 Rapoport, M., Dawson, H. N., Binder, L. I., Vitek, M. P. & Ferreira, A. Tau is essential to beta -amyloid-induced neurotoxicity. *Proc Natl Acad Sci U S A* **99**, 6364-6369, doi:10.1073/pnas.092136199 (2002).

31 Garwood, C. J., Pooler, A. M., Atherton, J., Hanger, D. P. & Noble, W. Astrocytes are important mediators of Abeta-induced neurotoxicity and tau phosphorylation in primary culture. *Cell Death Dis* **2**, e167, doi:10.1038/cddis.2011.50 (2011).

32 Briel, N., Pratsch, K., Roeber, S., Arzberger, T. & Herms, J. Contribution of the astrocytic tau pathology to synapse loss in progressive supranuclear palsy and corticobasal degeneration. *Brain Pathol*, e12914, doi:10.1111/bpa.12914 (2020).

33 Liddelow, S. A. *et al.* Neurotoxic reactive astrocytes are induced by activated microglia. *Nature* **541**, 481-487, doi:10.1038/nature21029 (2017).

34 Escartin, C. *et al.* Reactive astrocyte nomenclature, definitions, and future directions. *Nat Neurosci* **24**, 312-325, doi:10.1038/s41593-020-00783-4 (2021).

35 Washbourne, P. *et al.* Cell adhesion molecules in synapse formation. *J Neurosci* **24**, 9244-9249, doi:10.1523/JNEUROSCI.3339-04.2004 (2004).

For Review Only

36 Hillen, A. E. J., Burbach, J. P. H. & Hol, E. M. Cell adhesion and matricellular support by astrocytes of the tripartite synapse. *Prog Neurobiol* **165-167**, 66-86, doi:10.1016/j.pneurobio.2018.02.002 (2018).

37 Demyanenko, G. P. *et al.* Neural cell adhesion molecule NrCAM regulates Semaphorin 3F-induced dendritic spine remodeling. *J Neurosci* **34**, 11274-11287, doi:10.1523/JNEUROSCI.1774-14.2014 (2014).

38 Takano, T. *et al.* Chemico-genetic discovery of astrocytic control of inhibition in vivo. *Nature* **588**, 296-302, doi:10.1038/s41586-020-2926-0 (2020).

39 Cartier, A. E. *et al.* Regulation of synaptic structure by ubiquitin C-terminal hydrolase L1. *J Neurosci* **29**, 7857-7868, doi:10.1523/JNEUROSCI.1817-09.2009 (2009).

40 Li, A. J., Suzuki, S., Suzuki, M., Mizukoshi, E. & Imamura, T. Fibroblast growth factor-2 increases functional excitatory synapses on hippocampal neurons. *Eur J Neurosci* **16**, 1313-1324, doi:10.1046/j.1460-9568.2002.02193.x (2002).

41 Zou, L. H. *et al.* Effects of FGF2/FGFR1 Pathway on Expression of A1 Astrocytes After Infrasound Exposure. *Front Neurosci* **13**, 429, doi:10.3389/fnins.2019.00429 (2019).

42 Hoekstra, J. G. *et al.* Astrocytic dynamin-like protein 1 regulates neuronal protection against excitotoxicity in Parkinson disease. *Am J Pathol* **185**, 536-549, doi:10.1016/j.ajpath.2014.10.022 (2015).

43 Arriagada-Diaz, J., Prado-Vega, L., Cardenas Diaz, A. M., Ardiles, A. O. & Gonzalez-Jamett, A. M. Dynamin Superfamily at Pre- and Postsynapses: Master Regulators of Synaptic Transmission and Plasticity in Health and Disease. *Neuroscientist*, 1073858420974313, doi:10.1177/1073858420974313 (2020).

44 Palavicini, J. P. *et al.* RanBP9 aggravates synaptic damage in the mouse brain and is inversely correlated to spinophilin levels in Alzheimer's brain synaptosomes. *Cell Death Dis* **4**, e667, doi:10.1038/cddis.2013.183 (2013).

45 Wang, R. *et al.* RanBP9 overexpression accelerates loss of dendritic spines in a mouse model of Alzheimer's disease. *Neurobiol Dis* **69**, 169-179, doi:10.1016/j.nbd.2014.05.029 (2014).

46 Guan, J. S. *et al.* HDAC2 negatively regulates memory formation and synaptic plasticity. *Nature* **459**, 55-60, doi:10.1038/nature07925 (2009).

47 Rajkovic, I., Denes, A., Allan, S. M. & Pinteaux, E. Emerging roles of the acute phase protein pentraxin-3 during central nervous system disorders. *J Neuroimmunol* **292**, 27-33, doi:10.1016/j.jneuroim.2015.12.007 (2016).

48 Erreni, M., Manfredi, A. A., Garlanda, C., Mantovani, A. & Rovere-Querini, P. The long pentraxin PTX3: A prototypical sensor of tissue injury and a regulator of homeostasis. *Immunol Rev* **280**, 112-125, doi:10.1111/imr.12570 (2017).

49 Fan, Y. Y. & Huo, J. A1/A2 astrocytes in central nervous system injuries and diseases: Angels or devils? *Neurochem Int* **148**, 105080, doi:10.1016/j.neuint.2021.105080 (2021).

50 Zhou, C. *et al.* Pentraxin 3 contributes to neurogenesis after traumatic brain injury in mice. *Neural Regen Res* **15**, 2318-2326, doi:10.4103/1673-5374.285001 (2020).

51 Shindo, A. *et al.* Astrocyte-Derived Pentraxin 3 Supports Blood-Brain Barrier Integrity Under Acute Phase of Stroke. *Stroke* **47**, 1094-1100, doi:10.1161/STROKEAHA.115.012133 (2016).

52 Bloom, G. S. Amyloid-beta and tau: the trigger and bullet in Alzheimer disease pathogenesis. *JAMA Neurol* **71**, 505-508, doi:10.1001/jamaneurol.2013.5847 (2014).

53 Ittner, L. M. & Gotz, J. Amyloid-beta and tau--a toxic pas de deux in Alzheimer's disease. *Nat Rev Neurosci* **12**, 65-72, doi:10.1038/nrn2967 (2011).

54 Busche, M. A. & Hyman, B. T. Synergy between amyloid-beta and tau in Alzheimer's disease. *Nat Neurosci* **23**, 1183-1193, doi:10.1038/s41593-020-0687-6 (2020).

For Review Only

55 Morris, M. *et al.* Tau post-translational modifications in wild-type and human amyloid precursor protein transgenic mice. *Nat Neurosci* **18**, 1183-1189, doi:10.1038/nn.4067 (2015).

56 Vossel, K. A. *et al.* Tau reduction prevents Abeta-induced defects in axonal transport. *Science* **330**, 198, doi:10.1126/science.1194653 (2010).

57 Singh, B. *et al.* Tau is required for progressive synaptic and memory deficits in a transgenic mouse model of alpha-synucleinopathy. *Acta Neuropathol* **138**, 551-574, doi:10.1007/s00401-019-02032-w (2019).

58 Tai, C. *et al.* Tau Reduction Prevents Key Features of Autism in Mouse Models. *Neuron* **106**, 421-437 e411, doi:10.1016/j.neuron.2020.01.038 (2020).

59 Bi, M. *et al.* Tau exacerbates excitotoxic brain damage in an animal model of stroke. *Nat Commun* **8**, 473, doi:10.1038/s41467-017-00618-0 (2017).

60 Gheyara, A. L. *et al.* Tau reduction prevents disease in a mouse model of Dravet syndrome. *Ann Neurol* **76**, 443-456, doi:10.1002/ana.24230 (2014).

61 DeVos, S. L. *et al.* Antisense reduction of tau in adult mice protects against seizures. *J Neurosci* **33**, 12887-12897, doi:10.1523/JNEUROSCI.2107-13.2013 (2013).

62 Bazargani, N. & Attwell, D. Astrocyte calcium signaling: the third wave. *Nat Neurosci* **19**, 182-189, doi:10.1038/nn.4201 (2016).

63 Perea, G., Navarrete, M. & Araque, A. Tripartite synapses: astrocytes process and control synaptic information. *Trends Neurosci* **32**, 421-431, doi:10.1016/j.tins.2009.05.001 (2009).

64 Gomez-Gonzalo, M. *et al.* Neuron-astrocyte signaling is preserved in the aging brain. *Glia* **65**, 569-580, doi:10.1002/glia.23112 (2017).

65 Lines, J. *et al.* Astrocyte-neuronal network interplay is disrupted in Alzheimer's disease mice. *Glia* **70**, 368-378, doi:10.1002/glia.24112 (2022).

66 Anderson, M. A., Ao, Y. & Sofroniew, M. V. Heterogeneity of reactive astrocytes. *Neurosci Lett* **565**, 23-29, doi:10.1016/j.neulet.2013.12.030 (2014).

67 Zamanian, J. L. *et al.* Genomic Analysis of Reactive Astrogliosis. *The Journal of Neuroscience* **32**, 6391, doi:10.1523/JNEUROSCI.6221-11.2012 (2012).

68 Liddelow, S. A. *et al.* Neurotoxic reactive astrocytes are induced by activated microglia. *Nature* **541**, 481-487, doi:10.1038/nature21029 (2017).

69 Liddelow, S. A. *et al.* Neurotoxic reactive astrocytes are induced by activated microglia. *Nature* **541**, 481-487, doi:10.1038/nature21029 (2017).

70 Escartin, C. *et al.* Reactive astrocyte nomenclature, definitions, and future directions. *Nature Neuroscience* **24**, 312-325, doi:10.1038/s41593-020-00783-4 (2021).

71 Hasel, P., Rose, I. V. L., Sadick, J. S., Kim, R. D. & Liddelow, S. A. Neuroinflammatory astrocyte subtypes in the mouse brain. *Nature Neuroscience* **24**, 1475-1487, doi:10.1038/s41593-021-00905-6 (2021).

72 Liddelow, S. A. & Barres, B. A. Reactive Astrocytes: Production, Function, and Therapeutic Potential. *Immunity* **46**, 957-967, doi:10.1016/j.immuni.2017.06.006 (2017).

73 Rodriguez-Grande, B. *et al.* Pentraxin 3 mediates neurogenesis and angiogenesis after cerebral ischaemia. *J Neuroinflammation* **12**, 15, doi:10.1186/s12974-014-0227-y (2015).

74 Fornai, F. *et al.* Brain diseases and tumorigenesis: The good and bad cops of pentraxin3. *Int J Biochem Cell Biol* **69**, 70-74, doi:10.1016/j.biocel.2015.10.017 (2015).

75 Bonavita, E., Mantovani, A. & Garlanda, C. PTX3 acts as an extrinsic oncosuppressor. *Oncotarget* **6**, 32309-32310, doi:10.18632/oncotarget.4845 (2015).

76 Bonavita, E. *et al.* PTX3 is an extrinsic oncosuppressor regulating complement-dependent inflammation in cancer. *Cell* **160**, 700-714, doi:10.1016/j.cell.2015.01.004 (2015).

For Review Only

77 Litvinchuk, A. *et al.* Complement C3aR Inactivation Attenuates Tau Pathology and Reverses an Immune Network Deregulated in Tauopathy Models and Alzheimer's Disease. *Neuron* **100**, 1337-1353 e1335, doi:10.1016/j.neuron.2018.10.031 (2018).
78 Wu, T. *et al.* Complement C3 Is Activated in Human AD Brain and Is Required for Neurodegeneration in Mouse Models of Amyloidosis and Tauopathy. *Cell Rep* **28**, 2111-2123 e2116, doi:10.1016/j.celrep.2019.07.060 (2019).

FIGURES LEGENDS

Figure 1: Neuronal tau ablation prevents synaptic loss in neurons treated with Aβ-stimulated ACM. (A) Schematic representation of the methodology. (**B & C**) Representative images of 14 DIV WT and tau$^{-/-}$ neurons treated with 10 µg of total protein of nonstimulated (ns) or Aβ-stimulated (+Aβ) WT astrocyte conditioned medium (ACM). After 24 h of incubation, the number of Synapsin-1 (**D**, Syn-1), PSD95 (**E**) and synaptic clusters (**F**, colocalization) were quantified. n = 4 cultures, 10-12 neurons analyzed per culture. Data is shown as Mean +/- SD. Normality was assessed with a Shapiro–Wilk normality test. Significance was determined by one-way ANOVA. Significance = $p<0.05$. p values are indicated on each graph. Bar = 10 µm.

Figure 2: Astrocytic tau ablation prevents synaptic loss in WT neurons treated with Aβ-stimulated ACM. (A) Schematic representation of the methodology. (**B & C**) Representative images of 14 DIV WT neurons treated with 10 µg of total protein from nonstimulated (ns) or Aβ-stimulated (+Aβ) astrocyte conditioned medium (ACM). After 24 h of incubation, the number of Synapsin-1 (**C**, Syn-1), PSD95 (**D**) and synaptic clusters (**E**, colocalization) were quantified. n = 4 cultures, 10-12 neurons analyzed per culture. Data is shown as Mean +/- SD. Normality was

For Review Only

assessed with a Shapiro–Wilk normality test. Significance was determined by one-way ANOVA. Significance = p<0.05. p values are indicated on each graph. Bar = 10 µm.

<u>Figure 3</u>: Tau$^{-/-}$ astrocytes exhibit a neuroprotective phenotype. (A) Volcano plot of the differential gene expression levels between WT and tau$^{-/-}$ astrocytes in the NanoString Glial Profiling Panel. The analysis was performed using nSolver software. The false discovery rate corrected level of significance is shown as the horizontal line. Significance was set at p<0.05, and the log2 fold change (vertical lines) at -1 & 1. (**B)** Undirected Global Significance Scores for the top 9 gene set annotations provided by nSolver software from the comparison between WT and tau$^{-/-}$ astrocytes. (**C)** Violin plot representation of the scores of the A2 gene set annotation in WT or tau$^{-/-}$ astrocytes. Shapiro–Wilk normality test, Student's t-test, p<0.05. **D.** Gene expression heatmap of differentially expressed A2 annotation genes between WT and tau$^{-/-}$ astrocytes. (**E & F)** Normalized counts of mRNA of PTX3 and Cd109, respectively. Data is shown as Mean +/- SD. Shapiro–Wilk normality test, Student's t-test, p<0.05. p-values are indicated on each graph. n = 3 for all experiments.

For Review Only

<u>Figure 4</u>: PTX3 is increased in tau$^{-/-}$ astrocytes and tau$^{-/-}$ mice brain cortexes. (A) Representative images of WT and tau$^{-/-}$ astrocyte cultures treated with 1 µM of Aβ oligomers, stained for PTX3 (red) and GFAP (green). Nonstimulated (ns) astrocytes were used as a control. Bar = 20 µm (**B)** Quantification of the mean fluorescence intensity of PTX3 in the conditions shown in A. (**C)** PTX3 concentration in the supernatants from ns or stimulated WT and tau$^{-/-}$ astrocyte cultures. (**D)** Representative images of brain cortexes of 6-month-old WT and tau$^{-/-}$ mice stained for PTX3 (red) and GFAP (green). Bar = 10 µm **E.** Quantification of the total PTX3

positive area in D. All experiments n = 4 cultures, 10 photographs analyzed for each. B & C Normality was assessed by a Shapiro–Wilk normality test, significance with a one-way ANOVA, E Shapiro–Wilk normality test, Student's t-test. Data is shown as Mean +/- SD. Significance = $p<0.05$. p-values are indicated on each graph

Figure 5: PTX3 rescues the synaptotoxic effects of Aβ stimulated ACM. (A) Representative images of 14 DIV WT neurons treated with 10 μg of total protein from nonstimulated (ns) or Aβ-stimulated (+Aβ) astrocyte's conditioned medium (ACM) with or without 1 μg/mL recombinant PTX3 or heat-inactivated PTX3 (iPTX3). **(B)** Representative images of 14 DIV WT neurons treated with 10 μg of total protein of nonstimulated (ns) or Aβ-stimulated (+Aβ) WT ACM with or without PTX3 and iPTX3. After 24 h of incubation, the number of Synapsin-1 (**C**, Syn-1), PSD95 (**D**) and synaptic clusters (**E**, colocalization) were quantified. n = 4 cultures, 10-12 neurons analyzed per culture. Data is shown as Mean +/- SD. Normality was assessed by a Shapiro–Wilk normality test, significance with a one-way ANOVA. Significance = $p<0.05$. p values are shown on each graph Bar = 10 μm.

Figure 6: Astrocytic tau expression silencing via short hairpin causes an increase in astrocyte-derived PTX3 levels. (A) Schematic representation of the methodology. **(B)** Representative Western blot for total tau in GFP- or shTau-transduced astrocyte lysates. Thirty micrograms of total protein was loaded into each well. β-Actin was used as the loading control. **C.** PTX3 concentration in the supernatants of ns or stimulated WT GFP- or shTau-transduced astrocyte cultures. **(D)** Representative images of WT GFP- or shTau-transduced astrocyte cultures treated with 1 μM Aβ oligomers, stained for PTX3 (red) and GFAP (green). Nonstimulated (ns)

https://mc.manuscriptcentral.com/braincom
For Review Only

astrocytes were used as control. Bar = 20 µm. **E.** Quantification of the mean fluorescence intensity of PTX3 in the conditions shown in A. All experiments n = 4 cultures, 10 photographs each. B & C Shapiro–Wilk normality test, one-way ANOVA, E Shapiro–Wilk normality test, Student's t-test. Data is shown as Mean +/- SD. Significance = $p<0.05$. p-values are indicated on each graph.

Figure 7: Astrocytic tau expression silencing via short hairpin RNA prevents synaptic loss in WT neurons treated with Aβ-stimulated ACM. (A) Representative images of 14 DIV WT neurons treated with 10 µg of total protein from nonstimulated (ns) or Aβ-stimulated (+Aβ) transduced astrocyte conditioned medium (ACM). **(B)** Representative images of 14 DIV WT treated with 10 µg of total protein of AAV-shTau or AAV-shScr nonstimulated (ns) or Aβ-stimulated (+Aβ) WT ACM. After 24 h of incubation, the number of Synapsin-1 (**C**, Syn-1), PSD95 (**D**) and synaptic clusters (**E**, colocalization) were quantified. n = 4 cultures, 10 neurons analyzed per culture. Data is shown as Mean +/- SD. Normality was assessed by a Shapiro–Wilk normality test, significance with a one-way ANOVA. Significance = $p<0.05$. p values are shown on each graph Bar = 10 µm.

For Review Only

SUPPLEMENTARY FIGURES LEGENDS

Supplementary figure 1: Aβ stimulation does not modify astrocytic tau levels. 11 DIV WT or $tau^{-/-}$ astrocytes were stimulated with 1 µm of recombinant Aβ oligomers for 24 h. Nonstimulated astrocytes (ns) were used as a control. (**A**) Representative immunofluorescences for GFAP in all astrocyte groups. (**B**) qPCR for tau on both astrocyte groups. n = 3. Normality was assessed with a Shapiro–Wilk test. Significance was determined by Student's t-test, p = NS. (**C**) Representative Western blot for total tau in astrocyte lysates. Thirty micrograms of total protein was loaded into

each well. β-Actin was used as the loading control. **(D)** Quantification of 3 independent Western blots for total tau. Data is shown as Mean +/- SD. Normality was assessed with a Shapiro–Wilk normality test, significance with Student's t-test, p = NS.

<u>Supplementary figure 2</u>. Tau$^{-/-}$ astrocytes exhibit an increase in positive synaptic regulator gene expression. Normalized counts of the mRNA expression levels of several genes related to synapse homeostasis (**A**) and synapse degradation (**B**). Data is shown as Mean +/- SD. Normality was assessed with a Shapiro–Wilk normality test and significance with Student's t-test, $p<0.05$. p-values are indicated on each graph. n = 3 for all experiments.

For Review Only

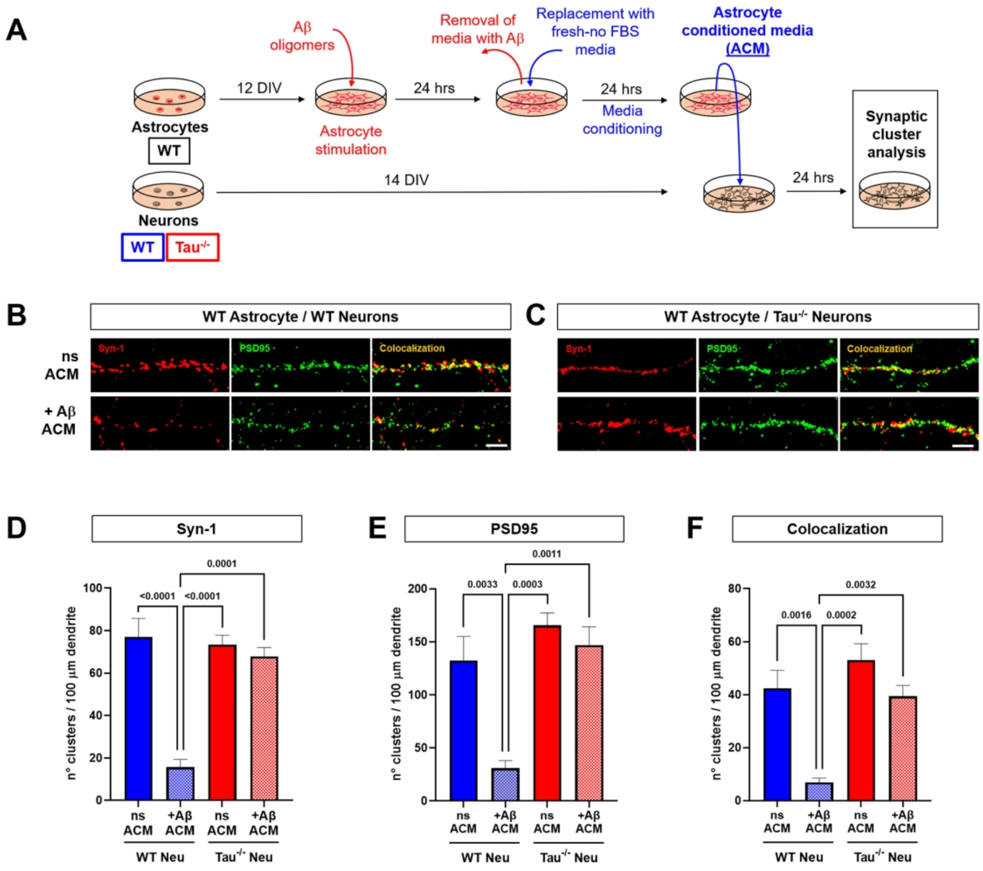

Figure 1: Neuronal tau ablation prevents synaptic loss in neurons treated with Aβ-stimulated ACM.

160x140mm (300 x 300 DPI)

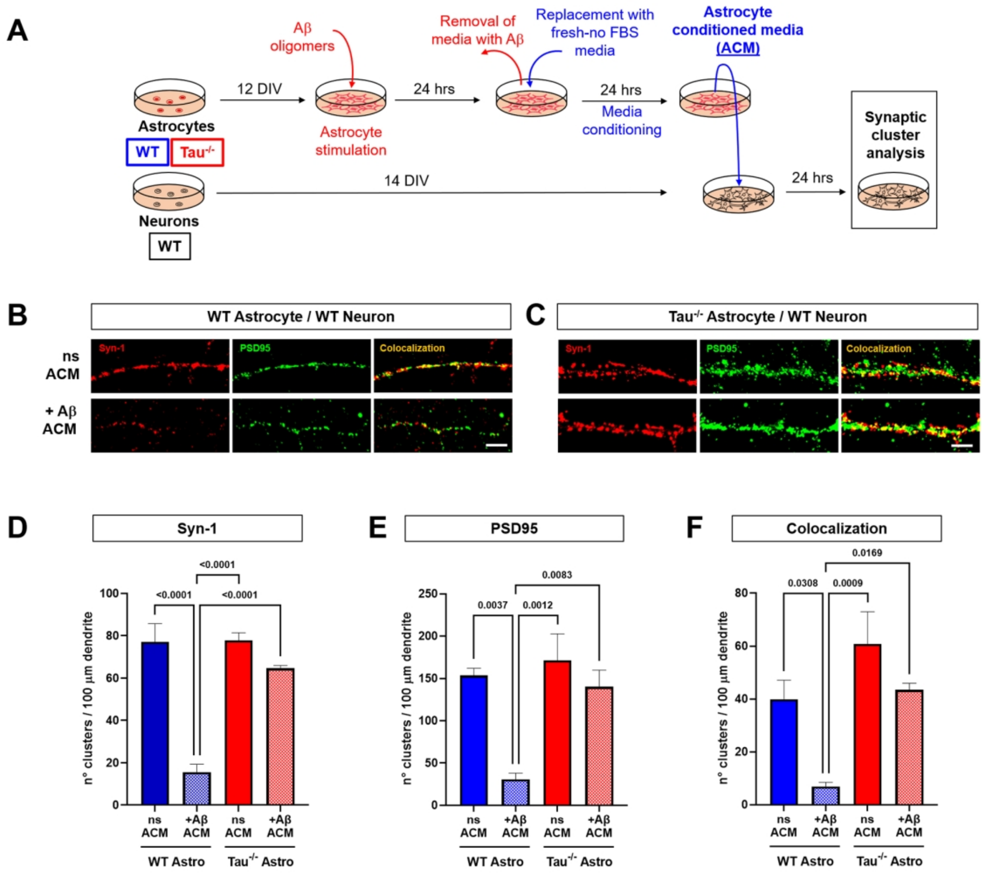

Figure 2: Astrocytic tau ablation prevents synaptic loss in WT neurons treated with Aβ-stimulated ACM.

160x141mm (300 x 300 DPI)

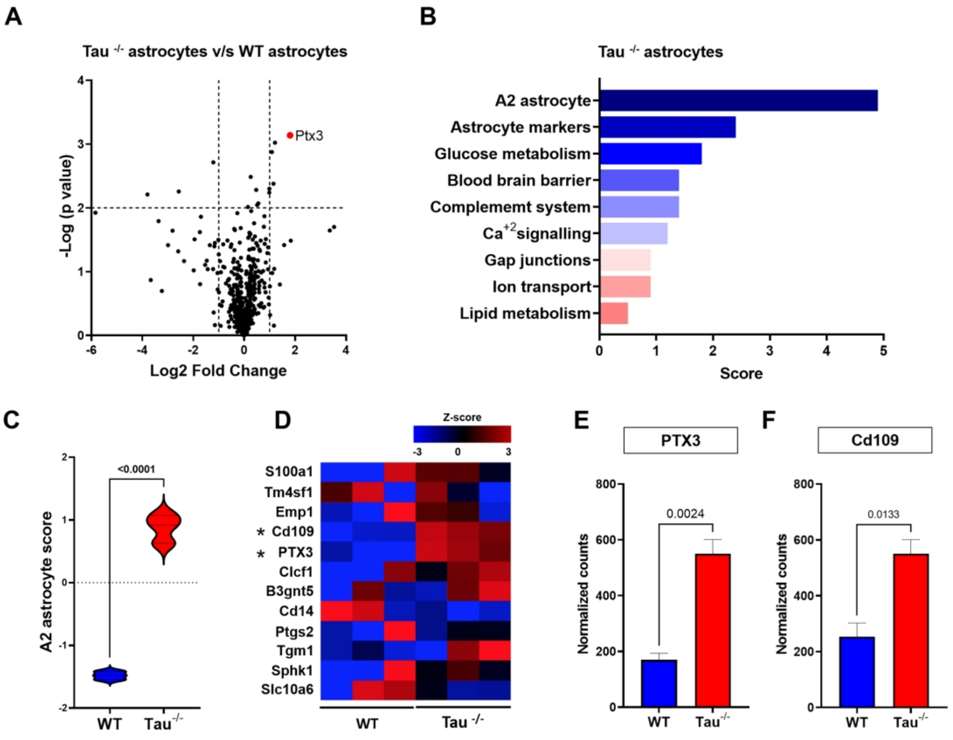

Figure 3: Tau-/- astrocytes exhibit a neuroprotective phenotype.

194x146mm (300 x 300 DPI)

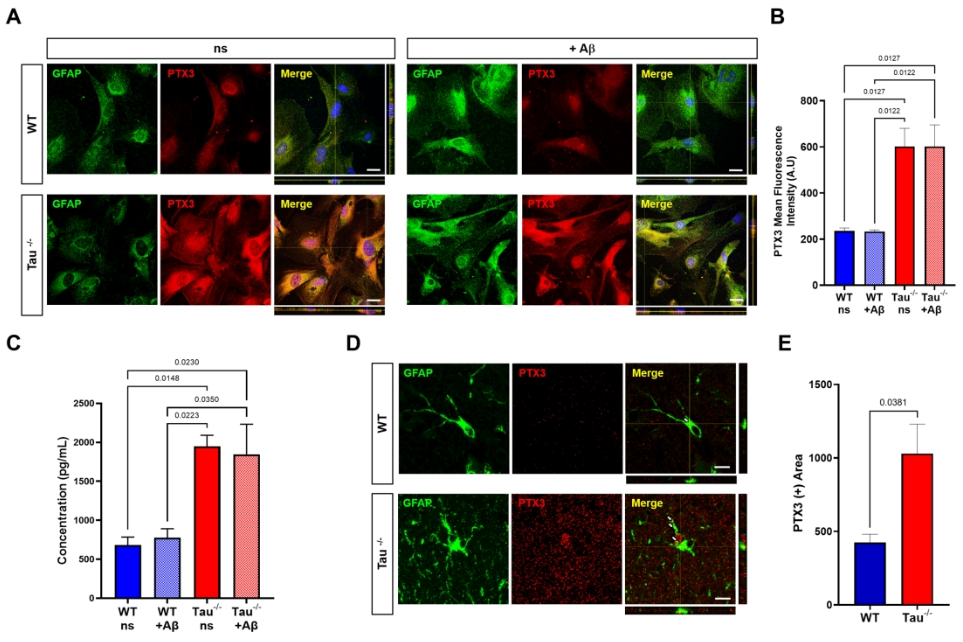

Figure 4: PTX3 is increased in tau-/- astrocytes and tau-/- mice brain cortexes.

230x148mm (300 x 300 DPI)

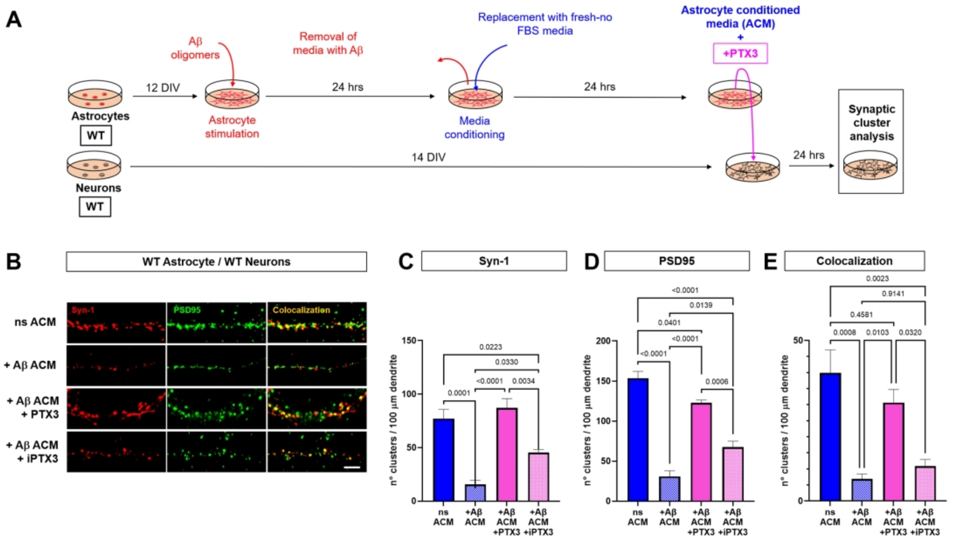

Figure 5: PTX3 rescues the synaptotoxic effects of Aβ stimulated ACM.

214x117mm (300 x 300 DPI)

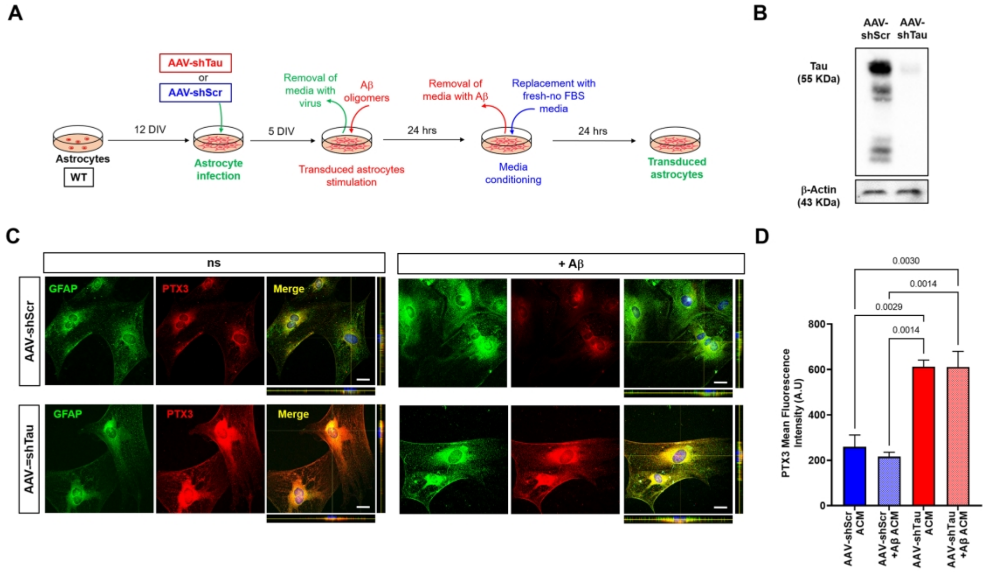

Figure 6: Astrocytic tau expression silencing via short hairpin causes an increase in astrocyte-derived PTX3 levels.

230x134mm (300 x 300 DPI)

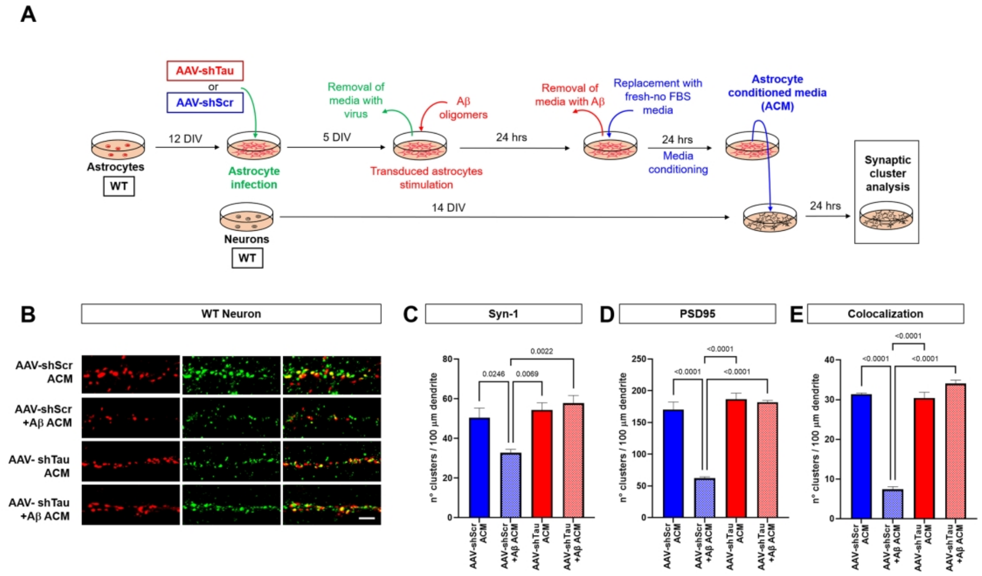

Figure 7: Astrocytic tau expression silencing via short hairpin RNA prevents synaptic loss in WT neurons treated with Aβ-stimulated ACM.

219x133mm (300 x 300 DPI)

SUPPLEMENTARY FIGURES LEGENDS

Supplementary figure 1: Aβ stimulation does not modify astrocytic tau levels. 11 DIV WT or tau$^{-/-}$ astrocytes were stimulated with 1 μm of recombinant Aβ oligomers for 24 h. Nonstimulated astrocytes (ns) were used as a control. (**A**) Representative immunofluorescences for GFAP in all astrocyte groups. (**B)** qPCR for tau on both astrocyte groups. n = 3. Normality was assessed with a Shapiro–Wilk test. Significance was determined by Student's t-test, p = NS. (**C)** Representative Western blot for total tau in astrocyte lysates. Thirty micrograms of total protein was loaded into each well. β-Actin was used as the loading control. **(D)** Quantification of 3 independent Western blots for total tau. Normality was assessed with a Shapiro–Wilk normality test, significance with Student's t-test, p = NS.

Supplementary figure 2. Tau$^{-/-}$ astrocytes exhibit an increase in positive synaptic regulator gene expression. Normalized counts of the mRNA expression levels of several genes related to synapse homeostasis (**A**) and synapse degradation (**B**). Normality was assessed with a Shapiro–Wilk normality test and significance with Student's t-test, $p<0.05$. p-values are indicated on each graph. n = 3 for all experiments.

For Review Only

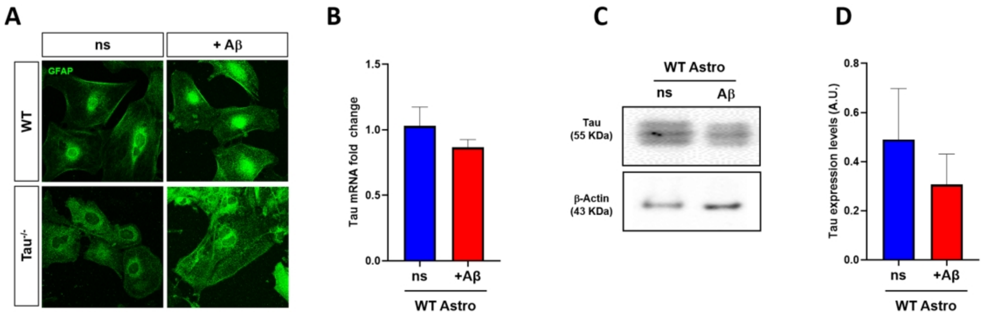

Supplementary figure 1: Aβ stimulation does not modify astrocytic tau levels.

170x56mm (300 x 300 DPI)

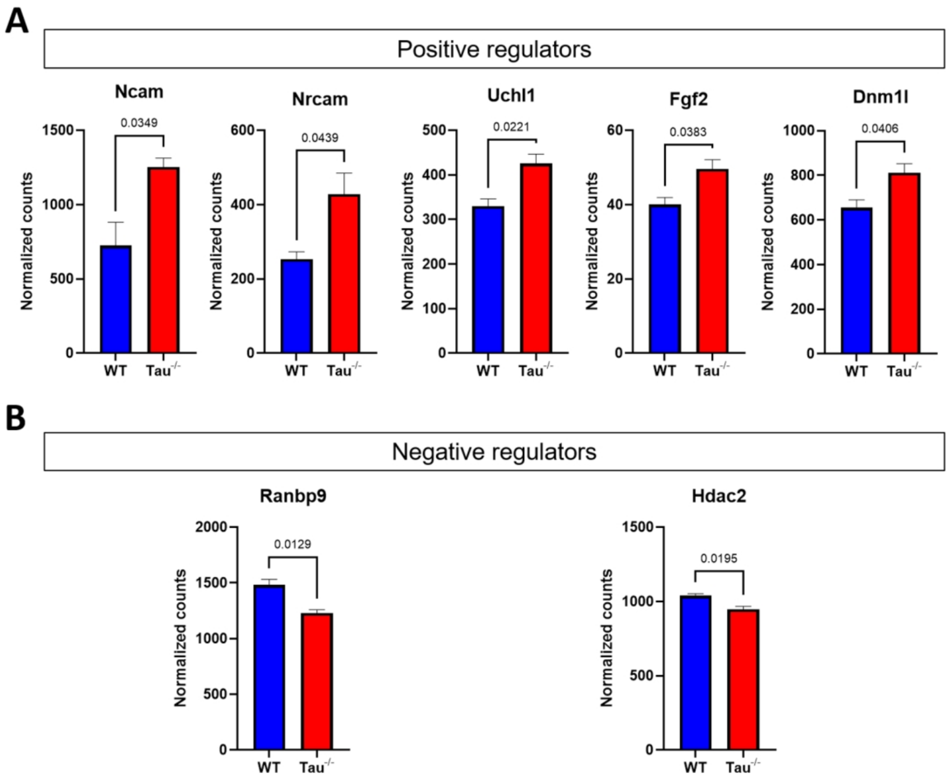

Supplementary figure 2. Tau-/- astrocytes exhibit an increase in positive synaptic regulator gene expression.

140x112mm (300 x 300 DPI)

Table 1: Positive and negative synaptic regulators

Name	Type	Role	References
**Ncam**	+	Cell adhesion protein, promotes formation and stabilization of synapses	(Hillen et al., 2018, Washbourne et al., 2004)
**Nrcam**	+	Cell adhesion protein, control of excitatory/inhibitory synapse balance	(Demyanenko et al., 2014, Takano et al., 2020)
**Uchhl1**	+	Deubiquitinating enzyme, involved in synaptic remodeling and function	(Cartier et al., 2009)
**Fgf2**	+	Signaling protein, promotes synapse function and inhibits reactive astrocyte turnover	(Li et al., 2002, Zou et al., 2019)
**Dnm1l**	+	GTPase, neuroprotective role, regulates synaptic vesicle recycling and plasticity	(Arriagada-Diaz et al., 2020, Hoekstra et al., 2015)
**Ranbp 9**	-	Small GTP binding protein, contributes to synaptic damage in AD	(Palavicini et al., 2013, Wang et al., 2014)
**Hdac2**	-	Histone deacetylase, negatively regulates synaptic plasticity	(Guan et al., 2009)

Supplementary Table 1: Partial transcriptome analysis of the differential expression of the 770 genes in the NanoString glial profiling panel. Analysis was performed using nSolver software. Gene names, log2fold changes and p-values are provided for each gene. Significance was set at $p<0.05$.

For Review Only
