# Supplementary material for: The reduction of astrocytic tau prevents amyloid-β-induced synaptotoxicity
Source: Brain Commun. 2022 Sep 19;4(5):fcac235. doi: 10.1093/braincomms/fcac235 (PMC9527666; doi:10.1093/braincomms/fcac235)
Supplement: fcac235_Supplementary_Data [file fcac235_supplementary_data.zip › Supplementary_figures.docx]

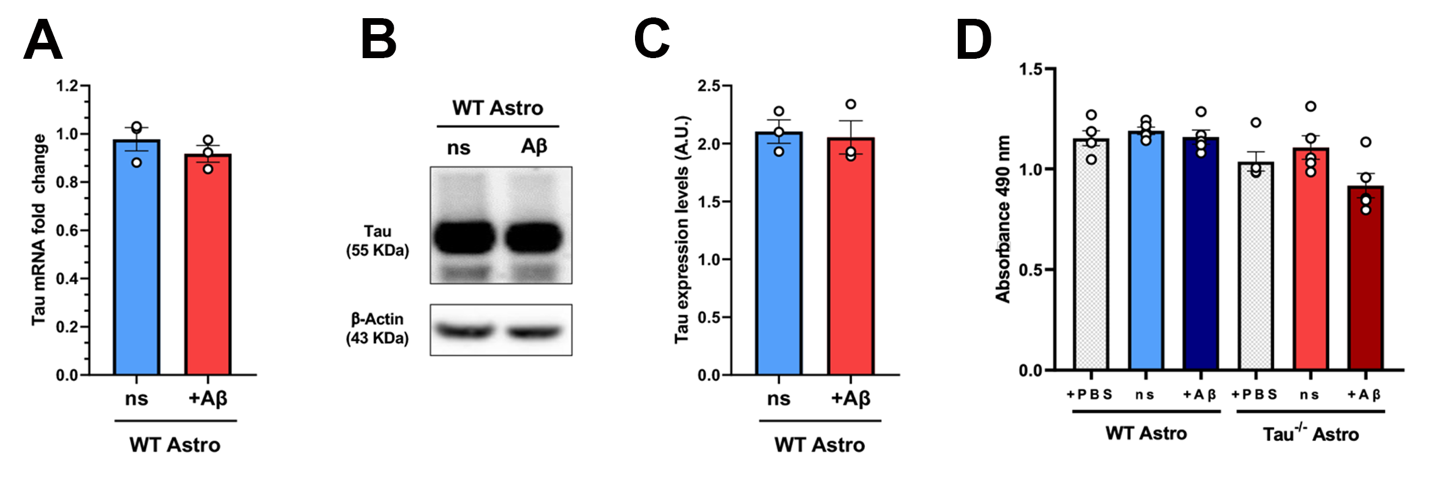


**Supplementary Figure 1: Ab treatment does not modify astrocytic endogenous tau levels nor astrocyte viability *in vitro*.** 11 DIV WT astrocytes were stimulated with 1μM of recombinant Aβ oligomers for 24 hrs. Non-stimulated astrocytes (ns) were used as a control. (**A**) qPCR for tau on WT astrocyte groups. n = 3, Mann-Whitney test p = NS. (**B**) Representative western blot for total tau on astrocyte lysates. β-Actin was used as loading control. **(C)** Quantification of total tau protein. n = 3, Mann-Whitney test p = NS. **(D)** WT and Tau^-/-^ astrocyte viability after Ab treatment measured by MTS assay. n = 4, Kruskal–Wallis test.


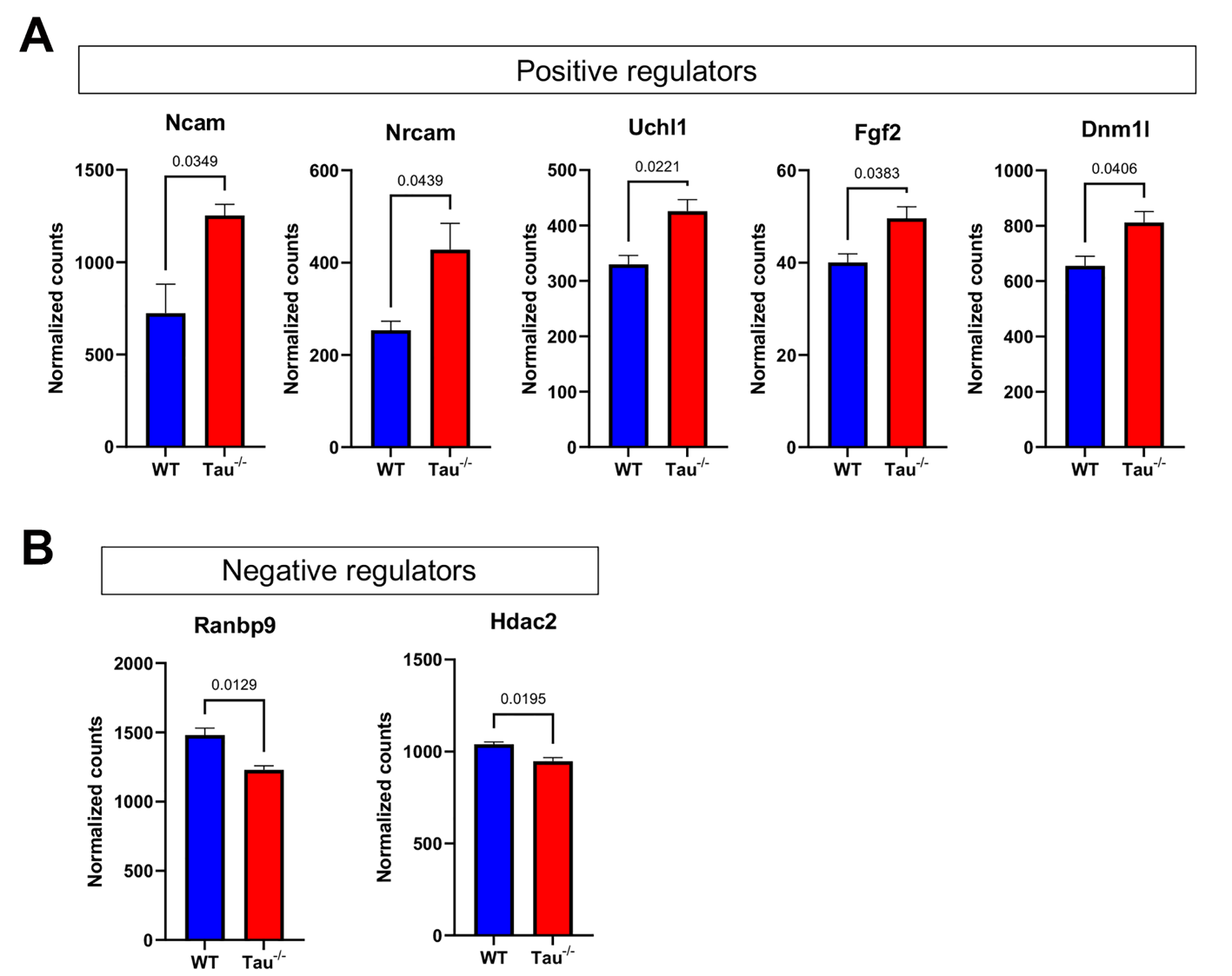


**Supplementary Figure 2. Tau^-/-^ astrocytes exhibit an increase in positive synaptic regulator genes.** Normalized counts of the mRNA expression of several genes related with synapse homeostasis (**A**) and synapse degradation (**B**). Shapiro-Wilk normality test, t-Student P<0.05. n = 3 on all experiments.


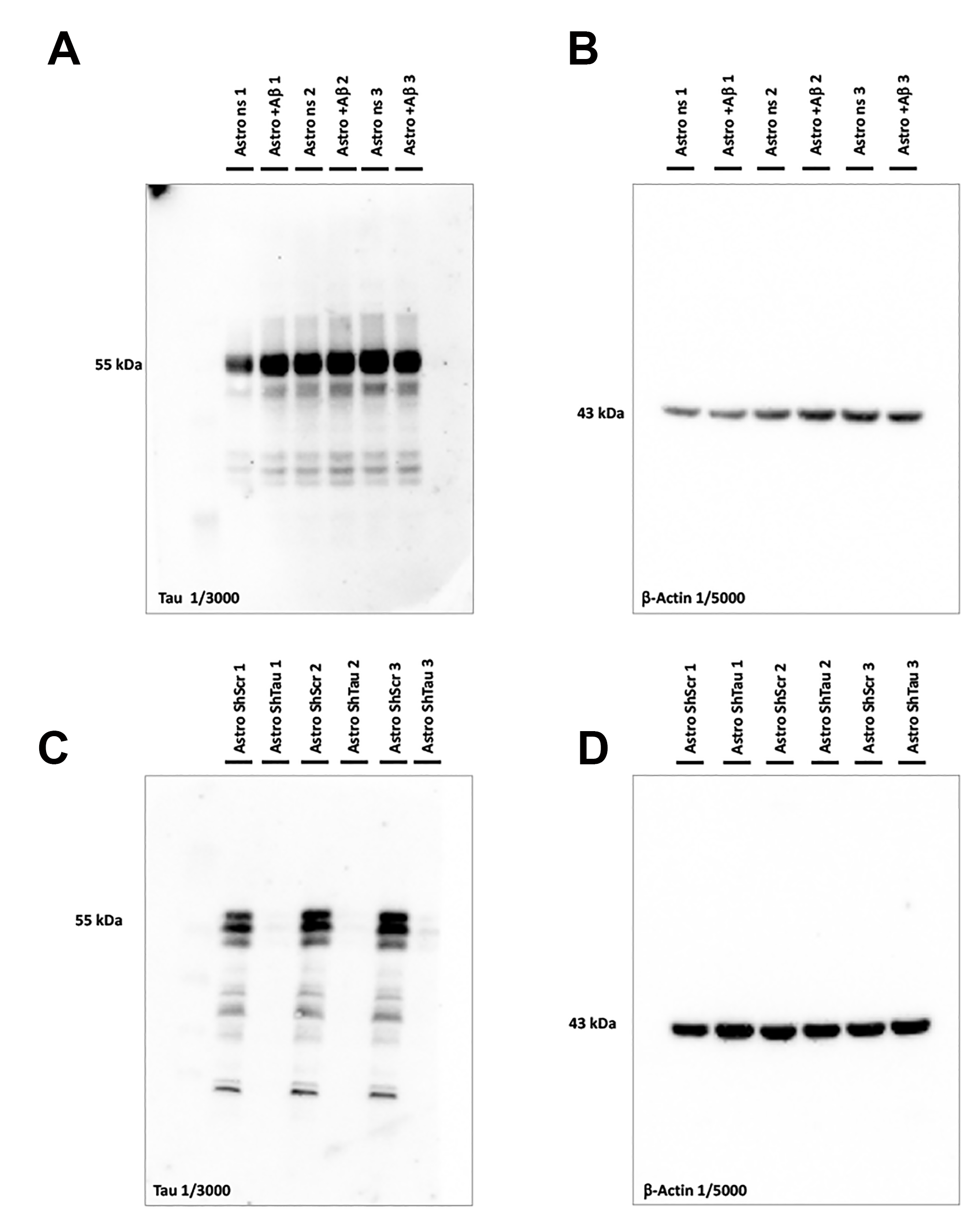


**Supplementary Figure 3: uncropped western blots used for Supplementary Figure 1B and Figure 7B.** (A) Total tau detected on cell lysates from non-stimulated (ns) or Aβ-stimulated (+Aβ) 11 DIV WT astrocyte culture, used in supplementary Figure 1B (**B**) β-Actin detected on the same blot in (A), corresponding to the loading control used in supplementary Figure 1B. (**C**) Total tau detected on cell lysates from 11 DIV WT astrocyte cultures infected with an AAV-shTau or an AAV-shScr, used in Figure 7B. (**D**) β-Actin detected on the same blot in (**C**), corresponding to the loading control used in Figure 7B.
