# Supplementary material for: The reduction of astrocytic tau prevents amyloid-β-induced synaptotoxicity
Source: Brain Commun. 2022 Sep 19;4(5):fcac235. doi: 10.1093/braincomms/fcac235 (PMC9527666; doi:10.1093/braincomms/fcac235)
Supplement: fcac235_Supplementary_Data [file fcac235_supplementary_data.zip › Supplementary_Table.docx]

**Supplementary Table 1: Positive and negative synaptic regulators**

| Name | Type | Role | References |
| --- | --- | --- | --- |
| Ncam | + | Cell adhesion protein, promotes formation and stabilization of synapses | (Hillen et al., 2018, Washbourne et al., 2004) |
| Nrcam | + | Cell adhesion protein, control of excitatory/inhibitory synapse balance | (Demyanenko et al., 2014, Takano et al., 2020) |
| Uchl1 | + | Deubiquitinating enzyme, involved in synaptic remodeling and function | (Cartier et al., 2009) |
| Fgf2 | + | Signaling protein, promotes synapse function and inhibits reactive astrocyte turnover | (Li et al., 2002, Zou et al., 2019) |
| Dnm1l | + | GTPase, neuroprotective role, regulates synaptic vesicle recycling and plasticity | (Arriagada-Diaz et al., 2020, Hoekstra et al., 2015) |
| Ranbp9 | - | Small GTP binding protein, contributes to synaptic damage in AD | (Palavicini et al., 2013, Wang et al., 2014) |
| Hdac2 | - | Histone deacetylase, negatively regulates synaptic plasticity | (Guan et al., 2009) |

Supplementary Table 1: Transcriptome analysis using NanoString glia profiling panel reveals differential expression of genes related with synaptic integrity in tau^-/-^ astrocytes vs WT astrocytes.
