# Supplementary material for: The reduction of astrocytic tau prevents amyloid-β-induced synaptotoxicity
Source: Brain Commun. 2022 Sep 19;4(5):fcac235. doi: 10.1093/braincomms/fcac235 (PMC9527666; doi:10.1093/braincomms/fcac235)
Supplement: fcac235_Supplementary_Data [file Original_submission_decision_letter.pdf › Original_submission_decision_letter.pdf]

Subject: Brain Communications - Decision on Manuscript ID BRAINCOM-2022-015

Body: 07-Feb-2022

Dear Prof. Lasagna-Reeves,

Thank you for your recent submission BRAINCOM-2022-015 entitled "ASTROCYTIC TAU REDUCTION PREVENTS Aβ-INDUCED SYNAPTOTOXICITY" to Brain Communications.

Your manuscript has been reviewed, and while the reviewers and editorial team appreciate many aspects of the paper, they raise some critiques that you will need to address in order for the paper to be considered for publication.

If you can address these concerns, I invite you to submit a revised manuscript and a response to each of the reviewers' comments. The reviewer(s)' comments are included at the bottom of this message.

In addition to the reviewers' comments, please also address our Scientific Editor's comments added below to comply with our requirements for presentation of scientific data at Brain Communications.

Please format your revised manuscript according to our Instructions to Authors https://academic.oup.com/braincomms/pages/General_Instructions – a summary of key points is attached to help with this.

To revise your manuscript, click the link below or log into https://mc.manuscriptcentral.com/braincom and enter your Author Centre, where you will find your manuscript title listed under "Manuscripts with Decisions." Under "Actions," click on "Create a Revision." Your manuscript number will be appended to denote a revision.

*** PLEASE NOTE: This is a two-step process. After clicking on the link, you will be directed to a webpage to confirm. ***

https://mc.manuscriptcentral.com/braincom?URL_MASK=e786d92f606147f1874b51c6a9b9008a

Please upload a revised manuscript with changes tracked with track changes or in coloured text.

When submitting your revised manuscript, you will be asked to respond to the reviewer(s) comments. Please be as specific as possible in your response to all of the comments.

The editorial team at Brain Communications aims to increase transparency in scientific publishing and to demonstrate the value that peer review brings to the scientific process. Please therefore consider whether you are willing to have reviewer(s)' comments on your paper and your response published as supplementary data with the paper if it is accepted. This is entirely optional and will only be done if both the authors and reviewers agree. While submitting your revision there will be an opportunity to review or update the answer you gave previously, if necessary.

IMPORTANT: Your original files are available to you when you upload your revised manuscript. Please delete any redundant files before completing the submission.

Because we are trying to facilitate timely publication of manuscripts submitted to Brain Communications, we would appreciate your revised manuscript as soon as possible. If it is not possible for you to resubmit within 3 months, the option to submit a revision will expire, and we would then consider your revised paper as a new submission. However, if you need a little more time, please get in touch and we can extend this deadline for you.

Once again, thank you for submitting your manuscript to Brain Communications and I look forward to receiving your revision.

Best wishes

Professor Tara Spires-Jones
Founding Editor, Brain Communications

Reviewer(s)' Comments to Author:

Reviewer: 1

Comments to the Author
See attached document

Reviewer: 2

Comments to the Author
This is an elegant study that shows a potential role of astrocytic tau in abeta mediated synaptotoxicity.
The main caveat of the study is the use of high levels Abeta to stimulate the cells. We know that levels of Abeta in AD brains do not reach these levels, so it compromises the relevance of the findings. I think the interpretation of the results would improve if the authors showed that there is not toxicity after treatment of astrocytes with abeta and that the effects seen are the consequence of unspecific cell death. The same with toxicity in neurons treated with ACM.
Measuring astrocytic reactivity by GFAP expression in vitro is not the best approach and it seems like there is not change in the morphology of the cells after adding Abeta.
The authors claim that tau-/- astrocytes acquire a more A2 phenotype, but (without taking into account that A1/A2 dichotomy might be a bit controversial) they don't show A1 phenotype, it could be that they also show a less A1 phenotype?
The authors do not mention the concentration of PTX3 used for the experiments, is it similar to the concentration found in Tau-/- astrocytes?
I think the authors could explain a bit better what is the potential link between tau and PTX3

Comments from the Scientific Editor:

- Please, provide details of the experimental unit (n= mice, cells, wells, plate downs, etc)) for each group analysed in these plots, and if the number is <10 show the single data-points. Please note our policy on pseudoreplication, we do not accept analyses where individual cells are the experimental unit when there is likely biological variability is between animals, culture replicates, etc. Please see instructions for authors for more details. Fort his type of data, we require either that a mean/median be taken per animal/culture replicate followed by statistical analyses or that more sophisticated analyses are performed such as mixed effects modelling which can account for random variables.

-Please, add the scale bar to Supp Fig1a.

- please, provide Full- size and uncropped blots/gels as Supplementary material.

-Please supply a graphical abstract. A graphical abstract is a visual representation of the central finding or methodology of the paper. It will be displayed in the table of contents. Authors should strive to make the graphical abstract informative, interesting, visually appealing, and straightforward (no caption needed, no abbreviations except gene names). Please upload as a separate high resolution image file (tiff preferred). The technical requirements for the graphical abstracts are:
--size of the submitted image: 1200 pixels square at 300dpi
--font of the text: Arial 12-16 point (smaller fonts will not be legible online)

-Please add an Abbreviated Summary (up to 50 words) of your paper that captures the main purpose and conclusions of the work. The summary should state the main results written in the third person ('[1st author surname] et al. report that …') and conclusion. This summary will be used in the contents list online and should not contain any abbreviations other than those on the accepted abbreviations list and accepted gene/protein names, making it accessible to the non-specialist. Please upload it as a Word document. Allowed abbreviations: AIDS; ANOVA; AMPA; ATP; A,T,C,G; CNS; CSF; CT; DNA; ECG; EEG; EMG; GABA; HIV; MRI; NMDA; PET; PCR; RNA.

- To comply with our ethical standards, the appropriate checklist must be included (see Reporting Guideline section within 'Instructions to authors').

-Please include a paragraph about data availability in the 'Materials and Methods' section. We strongly encourage data sharing either as supplementary information or uploads to repositories which you can link in the paper.

Reviewer 1 attachment:

1 BRAINCOMM 2022-015

The study by Cisternas et col. shows that conditioned media from Abeta-treated astrocytes decrease synaptic density in neuronal cultures, and that the effect is mediated by both astrocytic and neuronal tau. The evidence was obtained in primary astrocyte and neuronal cultures from cortex of tau -/- mice, as well as by the silencing of tau in mouse WT astrocytes using shRNA technology. Gene profiling showed that tau depletion modifies the transcriptome of astrocytes, and revealed upregulation of the gene encoding the synaptomodulatory factor, Petraxin 3 (PTX3). Increased expression of PTX3 was confirmed in tau depleted astrocytes, and addition of PTX3 to neuronal cultures reversed the detrimental effects of the conditioned media from Abeta-treated astrocytes.

NOVELTY: The study supports three novel concepts with regards to astrocytic tau. First, a modulatory role of astrocytic phenotype(s). Second, a role in AD-related pathology. Third, the existence of an intriguing interplay between neuronal and astrocytic tau.

CONTEXT: Tau is found in astrocytes in tauopathies. The term of aging-related tau astrogliopathy (ARTAG) has been coined to reflect the idea that astrocytes contribute to, or are damaged by, tau pathology (Ferrer et col., 2018). The consequences for disease symptoms and progression of ARTAG are not established, and their study represents an emerging line of research in the tau field. For example, the recent finding that targeted accumulation of 3Rtau in hippocampal astrocytes causes alteration of gamma waves and cognitive deficits supports that ARTAG contributes to Alzheimer (Richetin et col., 2020). The study by Cisternas et col., lends credence to this idea, and opens a novel therapeutic avenue aimed to increase the production of synaptogenic factors from astrocytes by targeted reduction of tau.

LIMITATIONS: The rationale, experimental design and data interpretation are overly influenced by the now obsolete A1/A2 neurotoxic/neuroprotective framework and terminology. Further, there are several experimental concerns. One is the lack of demonstration that there is tau in astrocytes, because the two Western blots shown to this effect are contradictory (Fig. 6B and Supplementary Fig. 1), and there is no immunohistochemical data in WT vs tau -/- mice. This is an important issue, because it is not known whether the tau accumulated in astrocytes in AD is astrocytic or is taken up from the extracellular milieu. Moreover, even if there is endogenous tau expression in astrocytes, its pathological impact may differ from the one accumulated in ARTAG, whatever its origin, because they are plausibly in very different conformations. The limitations of the model need to be discussed. Important controls are also missing in the studies with astrocyte conditioned media (ACM).

Another issue is that the experimental variability among cultures, batches of astrocyte conditioned media, or changes in synaptic density in different neurites from the same neurons is not sufficiently clarified. A remarkable feature of the study is that all the changes are impressive—perhaps too impressive. We also study the effect of ACM in synaptic density in a model of neurodegeneration and, at least in our hands, there is more variability among astrocyte and neuronal cultures.

SPECIFIC CRITIQUES

1. Rationale.

   The study design and data interpretation are based on the unproven premises that astrocytes 'are important mediators of Abeta-induced neurotoxicity', as stated in the abstract (line 31) and in the Results section (line 223), and that astrocytes switch between A1 neurotoxic /A2 neuroprotective/antiinflammatory astrocytes, as repeatedly stated throughout the text.
   The evidence supporting the notion that astrocytes mediate Abeta neurotoxicity comes from a study from 2011 showing that the presence of astrocytes moderately exacerbated the damage elicited by Abeta in primary neuronal cultures, as measured by increases in LDH production, caspase 3 activation and tau phosphorylation (Garwood et col., 2011). Although this study pioneered the notion that Abeta induces a phenotypic change in astrocytes that might be detrimental for neurons, it did not prove that astrocytes, let alone in vivo, are key to the synaptotoxic effects of Abeta. On the contrary, numerous studies document a direct detrimental effect of Abeta in neurons via endogenous TauC3.

   A recent consensus article published in Nature Neuroscience (Escartin et col., 2020) discouraged the misuse of the A1/A2 classification. A2 strictly is the transcriptomic state of astrocytes isolated from a mouse model of stroke. There is NO evidence whatsoever supporting that A2 astrocytes are neuroprotective, and signatures of 12 genes, let alone one gene, are not enough to define a phenotype. On the other hand, A1 strictly is the transcriptomic state of astrocytes isolated from a mouse model of septic shock. In Escartin et col. it was recommended in not to gratuitously extend the terms A1 and A2 to other diseases, and to use 'neurotoxic' only when such neurotoxic factor is identified.

   In the study by Cisternas et al. it is not appropriate to describe Abeta-treated astrocytes as 'neurotoxic' for three reasons. First, no neurotoxic factor was identified. Second, the reduced production of synapses in the presence of ACM from Abeta-treated astrocytes may be interpreted to indicate loss of the welldemonstrated permissive capacity of ACM media for neuritogenesis (see point 2 for missing controls). Third, as shown by the maintenance of synapses in tau-/- neurons exposed to ACM from Abeta-treated astrocytes, what is truly synaptotoxic is neuronal tau. Rigorous data interpretation and use of semantics are essential.

   As admitted by the authors themselves, 'Alterations in astrocytic function may therefore contribute to synaptic loss in AD; however, the extent of this contribution is unclear' (line 70). This is exactly the situation. What current evidence supports is that Abeta impairs the lysosomal and mitochondrial systems of astrocytes (Abramov, 2004; Sollvander, 2016; Gomez-Arboledas, 2018; Sánchez-Mico, 2020); promotes GABA release from astrocytes (Jo,

2014); and presents complex phenotypic changes in Abeta and tau mouse models encompassing adaptive and maladaptive changes (study from Jiwaji, 2022). How exactly these changes affect AD still is an outstanding question in the field. Plausibly, there is a complex interaction of factors with opposing impact on AD pathology.

In summary, the study by Cisternas et col. does not prove that ‘astrocytes are important mediators of Abeta-induced neurotoxicity’ (line 74), because there are other compatible explanations of the findings, or that down-regulation of tau in astrocytes promotes a ‘neuroprotective genetic profile’ (line 36), because up-regulation one or two genes does not render a phenotype ‘neuroprotective’. Mentions to A1/A2 should be removed and the molecular profiling in Fig. 3 reanalyzed grouping the genes according to neutral functional categories. The terms ‘positive and negative synaptic modulators’, as used in the supplementary table, are more appropriate than ‘neuroprotective’ and ‘neurotoxic’, because they touch on the loss of homeostatic modulation of synaptic plasticity by astrocytes in AD. Finally, the study does not ‘clarify the role of astrocytic tau in known neuronal networks effects of tau reduction in health and disease’ (line 40), because no studies in neural circuits and behavior were performed. The conclusions should be toned down, and the state-of-the art of astrocytes in AD updated.

2. Model and experimental design.

Importantly, the presence of tau in astrocytes is not convincingly demonstrated. Supplementary Fig. 1 does not show a significant expression of tau in WT astrocytes nor a reduction in tau -/- astrocytes. There is no tau immunohistochemistry in Fig. 4D comparing cortices from control and tau-/- mice. The Western blot in Fig. Fig. 6B showing the effect of silencing with shRNATau shows a remarkable expression in WT astrocytes unlike the one in Supplementary Fig. 1, when the expression of tau in control conditions should be similar—unless infection with a viral vector up-regulates tau in astrocytes. And contrary to the description in the text, there is indeed increased tau fluorescence in Abeta-treated astrocytes. Comparisons with neurons are in order.

Fields at lower magnification should be shown to prove that PTX3 is increased in all cortical astrocytes. Fluorescence quantification should be described in more detail (number areas analyzed, magnification, number of astrocytes per field, etc.). The same for synaptic markers.

Although it is stated in the manuscript that ‘The data that support the findings of this study are available in the supplementary material of this article’, raw data cannot be found. It might be helpful to provide some of the raw materials that were used to produce data, such as western blots from independent cultures or manipulations, or the whole set of images used in fluorescent analyses.

Justify the choice of 10 ug of ACM to treat neuronal cultures. Were different ACM and doses tested? A missing control is the synaptic density in the absence of any ACM. It is probable that ACM from control astrocytes increase synaptic density in neuronal cultures, such that the reduction of synapses in the presence of Abeta/ACM represents a loss rather than a gain of toxic function mediated by astrocytic tau. Justify the use of the Mouse Glial Profiling Panel. Glial cells are highly distinct, there is no such thing as a ‘glia’ marker. Perhaps only

astrocytic genes should be used in the analysis. Justify the dose used of PTX3 in reversal experiments. Was blockade of endogenous PTX3 tested?

3. Statistical analysis.

   Whether cultures or individual neurons are considered as independent observations in the means +/+ SD should be clarified; if it is single neurons, the variability among experiments with different cultures should be depicted. Likewise, as noted, how many batches of ACM were used from independent astrocyte cultures and treatments with Abeta? Indeed, the molecular analysis in Fig. 3D shows that differences among different batches of tau null astrocytes are not negligible.

4. Limitations.

   Whether the model truly mimics ARTAG should be discussed, as well as the caveats and possible artifacts in ACM experiments.

5. Style and presentation.
   In the Abstract, explain better why tau pathology in astrocytes deserves attention.
   The size of text in all figs should be increased. Many figs could be combined, and only data for one synaptic marker may be shown and the rest may be moved to supplementary data, because the data with the two synaptic markers and the merge are almost identical.

   The article is clearly written but there is room for improvement. For example, there are too many double noun modifiers, e.g. 'Astrocytic tau reduction' (title), 'Astrocytic tau deletion', better 'deletion and reduction of astrocytic tau'; 'neuronal tau absence' line 224 should be 'absence of tau in neurons'; 'primary culture experiments' should be 'experiments in primary cultures'; specify brain region and species; 'cortices' is a better choice than 'cortexes'; spell out CMF-HBSS (line 91); the authors probably mean that 'tissue was disaggregated' and not 'desegregated', as though there were racial conflicts among tissue elements; 'astrocyte and neuronal culture immunofluorescence' may be changed to 'immunofluorescence studies in astrocyte and neuronal cultures', to cite a few changes.

Abramov, A. Y., Canevari, L., & Duchen, M. R. (2004). β-Amyloid Peptides Induce Mitochondrial Dysfunction and Oxidative Stress in Astrocytes and Death of Neurons through Activation of NADPH Oxidase. The Journal of Neuroscience, 24(2), 565-575. doi:10.1523/jneurosci.4042-03.2004

Escartin C et col. Reactive astrocyte nomenclature, definitions, and future directions. Nat Neurosci. 2021 Mar;24(3):312-325. doi: 10.1038/s41593-020-00783-4. Epub 2021 Feb 15. PMID: 33589835; PMCID: PMC8007081.

Garwood CJ, Pooler AM, Atherton J, Hanger DP, Noble W. Astrocytes are important mediators of Aβ-induced neurotoxicity and tau phosphorylation in primary culture. Cell Death Dis. 2011 Jun 2;2(6):e167. doi: 10.1038/cddis.2011.50. PMID: 21633390; PMCID: PMC3168992.

Gomez-Arboledas, A., Davila, J. C., Sanchez-Mejias, E., Navarro, V., Nunez-Diaz, C., Sanchez-Varo, R., . . . Gutierrez, A. (2018). Phagocytic clearance of presynaptic dystrophies by reactive astrocytes in Alzheimer's disease. Glia, 66(3), 637-653. doi:10.1002/glia.23270

Ferrer I, García MA, González IL, Lucena DD, Villalonga AR, Tech MC, Llorens F, Garcia-Esparcia P, MartinezMaldonado A, Mendez MF, Escribano BT, Bech-Serra JJ, Sabido E, de la Torre Gómez C, Del Rio JA. Aging-related tau astrogliopathy (ARTAG): not only tau phosphorylation in astrocytes. Brain Pathol. 2018 Nov;28(6):965-985. doi: 10.1111/bpa.12593. Epub 2018 Feb 25. PMID: 29396893; PMCID: PMC8028270.

Jiwaji, Z., Tiwari, S.S., Avilés-Reyes, R.X. et al. Reactive astrocytes acquire neuroprotective as well as deleterious signatures in response to Tau and Aß pathology. Nat Commun 13, 135 (2022). https://doi.org/10.1038/s41467-021- 27702-w

Jo, S., Yarishkin, O., Hwang, Y. J., Chun, Y. E., Park, M., Woo, D. H., . . . Lee, C. J. (2014). GABA from reactive astrocytes impairs memory in mouse models of Alzheimer's disease. Nat Med, 20(8), 886-896. doi:10.1038/nm.3639

Richetin K, Steullet P, Pachoud M, Perbet R, Parietti E, Maheswaran M, Eddarkaoui S, Bégard S, Pythoud C, Rey M, Caillierez R, Q Do K, Halliez S, Bezzi P, Buée L, Leuba G, Colin M, Toni N, Déglon N. Tau accumulation in astrocytes of the dentate gyrus induces neuronal dysfunction and memory deficits in Alzheimer's disease. Nat Neurosci. 2020 Dec;23(12):1567-1579. doi: 10.1038/s41593-020-00728-x. Epub 2020 Nov 9. PMID: 33169029.

Sollvander, S., Nikitidou, E., Brolin, R., Soderberg, L., Sehlin, D., Lannfelt, L., & Erlandsson, A. (2016). Accumulation of amyloid-beta by astrocytes result in enlarged endosomes and microvesicle-induced apoptosis of neurons. Mol Neurodegener, 11(1), 38. doi:10.1186/s13024-016-0098-z 5

Sanchez-Mico, M. V., Jimenez, S., Gomez-Arboledas, A., Munoz-Castro, C., Romero-Molina, C., Navarro, V., . . . Vitorica, J. (2020). Amyloid-beta impairs the phagocytosis of dystrophic synapses by astrocytes in Alzheimer's disease. Glia. doi:10.1002/glia.23943
