# Supplementary material for: The reduction of astrocytic tau prevents amyloid-β-induced synaptotoxicity
Source: Brain Commun. 2022 Sep 19;4(5):fcac235. doi: 10.1093/braincomms/fcac235 (PMC9527666; doi:10.1093/braincomms/fcac235)
Supplement: fcac235_Supplementary_Data [file Response_to_reviewers_comments_original.pdf › Response_to_reviewers_comments_original.pdf]

BRAINCOM-2022-015: Astrocytic Tau Reduction Prevents Aβ-Induced Synaptotoxicity.

Response to Reviewers and Editor

We would like to thank the reviewers and the editor for their constructive suggestions, which helped us improve our manuscript. Our responses to specific comments are below. Original reviewer comments are formatted in *italics* followed by our answers in regular font.

<u>Reviewer Comments</u>

<u>Reviewer 1</u>

The study by Cisternas et col. shows that conditioned media from Abeta-treated astrocytes decrease synaptic density in neuronal cultures, and that the effect is mediated by both astrocytic and neuronal tau. The evidence was obtained in primary astrocyte and neuronal cultures from cortex of tau -/- mice, as well as by the silencing of tau in mouse WT astrocytes using shRNA technology. Gene profiling showed that tau depletion modifies the transcriptome of astrocytes and revealed upregulation of the gene encoding the synaptomodulatory factor, Petraxin 3 (PTX3). Increased expression of PTX3 was confirmed in tau depleted astrocytes, and addition of PTX3 to neuronal cultures reversed the detrimental effects of the conditioned media from Abeta-treated astrocytes.

NOVELTY: The study supports three novel concepts with regards to astrocytic tau. First, a modulatory role of astrocytic phenotype(s). Second, a role in AD-related pathology. Third, the existence of an intriguing interplay between neuronal and astrocytic tau.

CONTEXT: Tau is found in astrocytes in tauopathies. The term of aging-related tau astrogliopathy (ARTAG) has been coined to reflect the idea that astrocytes contribute to, or are damaged by, tau pathology (Ferrer et col., 2018). The consequences for disease symptoms and progression of ARTAG are not established, and their study represents an emerging line of research in the tau field. For example, the recent finding that targeted accumulation of 3Rtau in hippocampal astrocytes causes alteration of gamma waves and cognitive deficits supports that ARTAG contributes to Alzheimer (Richetin et col., 2020). The study by Cisternas et col., lends credence to this idea, and opens a novel therapeutic avenue aimed to increase the production of synaptogenic factors from astrocytes by targeted reduction of tau.

We thank the reviewer for this comment. The aim of our study was to determine the synaptoprotective effects of astrocytic tau downregulation under pathological stimuli rather than evaluate the effect of astrocytic tau aggregation, which are two distinct potential tau-related pathomechanisms. Numerous studies have demonstrated that reducing tau expression prevents or diminish Aβ-induced neurotoxicity *in vitro* and *in vivo*. It has also recently been suggested that physiological tau functions can enable brain disease by allowing other factors and processes to alter signaling pathway[1]. To better clarify the goals of the present study, we have updated the introduction (lines 64 to 70) and discussion (lines 401 to 415). For further reading, please refer to Dr. Lennart Mucke's work described in [1].

LIMITATIONS: The rationale, experimental design and data interpretation are overly influenced by the now obsolete A1/A2 neurotoxic/neuroprotective framework and terminology.

As the reviewer suggested, we have avoided designating A1 astrocytes as a universal neurotoxic phenotype. We have rewritten a more balanced discussion (lines 426 to 436), specifically citing newly published research on neuroinflammatory astrocyte subtypes, which had not been published at the time of the initial submission[2,3]. We have also incorporated a statement acknowledging the limitation of the A1/A2 dichotomy provided by the NanoString panel in the Results section (lines 327 to 330).

Further, there are several experimental concerns. One is the lack of demonstration that there is tau in astrocytes, because the two Western blots shown to this effect are contradictory (Fig. 6B and Supplementary Fig. 1), and there is no immunohistochemical data in WT vs tau -/- mice. This is an important issue, because it is not known whether the tau accumulated in astrocytes in AD is astrocytic or is taken up from the extracellular milieu. Moreover, even if there is endogenous tau expression in astrocytes, its pathological impact may differ from the one accumulated in ARTAG, whatever its origin, because they are plausibly in very different conformations. The limitations of the model need to be discussed.

We thank the reviewer for these comments. We have included new western blots that more clearly demonstrate the presence of tau in WT astrocytes (Supplementary Figure 1 B and Figure 7). These blots were performed using cell lysates obtained from pure astrocytes in culture in the absence of other cell types, validating the endogenous expression of astrocytic tau. As mentioned before, it was not the goal of this study to mimic a model of ARTAG. Additionally, it has been well described that the Tau-/- mouse model utilized in this study and available through the Jackson laboratory (JAX stock #007251) does not express tau in glia or neurons.

Important controls are also missing in the studies with astrocyte conditioned media (ACM).

Although performed at the same time, we originally decided to leave this control (non-treated neurons) out of our first draft. Nevertheless, it is included in this revision following the reviewer's request. Please refer to Figures 1, 2, 6 and 8 (non-treated neurons indicated in gray bars).

Another issue is that the experimental variability among cultures, batches of astrocyte conditioned media, or changes in synaptic density in different neurites from the same neurons is not sufficiently clarified. A remarkable feature of the study is that all the changes are impressive—perhaps too impressive. We also study the effect of ACM in synaptic density in a model of neurodegeneration and, at least in our hands, there is more variability among astrocyte and neuronal cultures.

We thank the reviewer for pointing out these concerns. We expanded our Methods section and added a detailed explanation of the n breakdown of each of the experiments in our study (line 242 to 268).
Briefly, regarding the n breakdown for *Synaptic quantification (Figs. 1, 2, 6 & 8)*, we performed 4 independent neuronal cultures from individual littermates (n = 4). A set of 10 photographs per condition per culture were randomly taken across the coverslip, depicting 1 neuron. A single

neurite was randomly chosen from each image and analyzed, and synaptic markers were quantified. The final n was obtained averaging the 10 quantifications of each marker and their colocalization. We would like to mention that we were confused by meaning of the reviewer's statement that our results were "*perhaps too impressive*". We are optimistic that the changes included in this revision will help alleviate any potential apprehension that may have arisen previously.

SPECIFIC CRITIQUES
1. Rationale.

The study design and data interpretation are based on the unproven premises that astrocytes 'are important mediators of Abeta-induced neurotoxicity', as stated in the abstract (line 31) and in the Results section (line 223), and that astrocytes switch between A1 neurotoxic /A2 neuroprotective/anti-inflammatory astrocytes, as repeatedly stated throughout the text.

The evidence supporting the concept that astrocytes mediate Aβ neurotoxicity comes from a study published in 2011 that shows that the presence of astrocytes moderately exacerbated the damage elicited by Aβ in primary neuronal cultures, measured by increases in LDH production, caspase 3 activation and tau phosphorylation[4]. Although this study pioneered the concept of Aβ causing a phenotypic change in astrocytes that could be detrimental to neurons, it did not demonstrate that astrocytes are solely responsible for these toxic effects *in vivo*. We acknowledge that the Garwood *et al*.'s study focused solely on *in vitro* effects. However, a recent i*n vitro* study supports the notion that astrocytes are important in Aβ-mediated synaptotoxicity, showing that reactivity produced in astrocytes due to Aβ exposure leads to a failure in their capacity to maintain synaptic puncta[5]. Additionally, *in vivo* studies using the APP/PS1 mouse model revealed that astrocytes showed an Aβ-density-related hyperactivity that might be driving a spatial distortion in the astrocyte network, contributing to cortical neuronal dysregulation[6]. To avoid any confusion, we toned down the statement "Astrocytes are important mediators of Aβ-induce neurotoxicity" to "It has been proposed that astrocytes could be active mediators of Aβ-induced neurotoxicity' (Results section line 279 to 280).

A recent consensus article published in Nature Neuroscience (Escartin et col., 2020) discouraged the misuse of the A1/A2 classification. A2 strictly is the transcriptomic state of astrocytes isolated from a mouse model of stroke. There is NO evidence whatsoever supporting that A2 astrocytes are neuroprotective, and signatures of 12 genes, let alone one gene, are not enough to define a phenotype. On the other hand, A1 strictly is the transcriptomic state of astrocytes isolated from a mouse model of septic shock. In Escartin et col. it was recommended in not to gratuitously extend the terms A1 and A2 to other diseases, and to use 'neurotoxic' only when such neurotoxic factor is identified.

As mentioned before, we have avoided the use of the A1 astrocyte designation as a universal neurotoxic phenotype. We have rewritten a more balanced discussion (lines 426 to 436), specifically citing newly published research on neuroinflammatory astrocyte subtypes, which had not been published at the time of the initial submission[2,3]. In the Results section, we have also incorporated a statement acknowledging the limitation of the A1/A2 dichotomy provided by the NanoString panel (lines 327 to 330).

In the study by Cisternas et al. it is not appropriate to describe Abeta-treated astrocytes as 'neurotoxic' for three reasons. First, no neurotoxic factor was identified.

We thank the reviewer for this comment. In the Discussion, we mention that a toxic astrocyte is defined not by the secretion of an identified toxic factor but by the detrimental effect it has on neurons or, as shown by our study, synapses. To avoid confusion and be more specific about our findings, the concept of "neurotoxic" astrocytes was adjusted to "synaptotoxic" astrocytes.

Second, the reduced production of synapses in the presence of ACM from Abeta-treated astrocytes may be interpreted to indicate loss of the well demonstrated permissive capacity of ACM media for neuritogenesis (see point 2 for missing controls).

We agree with the reviewer about how ACM can affect neuritogenesis. However, as clearly stated in our Methods sections, the neuronal cultures used for ACM stimulation were 14 DIV. *In vitro* neuritogenesis starts at DIV 1 until DIV 5. At 14 DIV, neurites are already formed, synapses are established, and neurons are fully mature[7,8]; thus, the results shown in our paper cannot be a representation of the adverse effects of neuritogenesis or, quoting the reviewer, "*production of synapses*", since these processes are already complete at the time of our ACM treatment. Nevertheless, as the reviewer suggested (point 2 regarding the missing control), we included non-treated neurons as controls.

Third, as shown by the maintenance of synapses in tau-/- neurons exposed to ACM from Abeta-treated astrocytes, what is truly synaptotoxic is neuronal tau. Rigorous data interpretation and use of semantics are essential.

Although thankful, we respectfully disagree with the reviewer's observation. Based on the pioneering research of Dr. Lennart Mucke's laboratory, physiological tau is not toxic by itself, but rather physiological tau function can enable toxicity by allowing other factors and processes to alter further signaling pathways (please refer to Dr. Lennart Mucke's work described in [1]).

As admitted by the authors themselves, 'Alterations in astrocytic function may therefore contribute to synaptic loss in AD; however, the extent of this contribution is unclear' (line 70). This is exactly the situation. What current evidence supports is that Abeta impairs the lysosomal and mitochondrial systems of astrocytes (Abramov, 2004; Sollvander, 2016; Gomez-Arboledas, 2018; S nchez-Mico, 2020); promotes GABA release from astrocytes (Jo, 2014); and presents complex phenotypic changes in Abeta and tau mouse models encompassing adaptive and maladaptive changes (study from Jiwaji, 2022). How exactly these changes affect AD still is an outstanding question in the field. Plausibly, there is a complex interaction of factors with opposing impact on AD pathology.

We agree with the reviewer. For this reason, in this revision, we included the following statement: "Alterations in astrocytic function may therefore contribute to synaptic loss in AD; however, the extent of this contribution is currently unclear" (line77 to 79).

In summary, the study by Cisternas et col. does not prove that 'astrocytes are important mediators of Abeta-induced neurotoxicity' (line 74), because there are other compatible explanations of the

findings, or that down-regulation of tau in astrocytes promotes a 'neuroprotective genetic profile' (line 36), because up-regulation one or two genes does not render a phenotype 'neuroprotective'. Mentions to A1/A2 should be removed and the molecular profiling in Fig. 3 reanalyzed grouping the genes according to neutral functional categories. The terms 'positive and negative synaptic modulators', as used in the supplementary table, are more appropriate than 'neuroprotective' and 'neurotoxic', because they touch on the loss of homeostatic modulation of synaptic plasticity by astrocytes in AD. Finally, the study does not 'clarify the role of astrocytic tau in known neuronal networks effects of tau reduction in health and disease' (line 40), because no studies in neural circuits and behavior were performed. The conclusions should be toned down, and the state-of-the art of astrocytes in AD updated.

As the reviewer suggested, several aspects of the manuscript had been toned down. We changed the statement "astrocytes are important mediators of Aβ-induced neurotoxicity" to "results suggested that astrocytes could be mediators of Aβ-induced neurotoxicity" (line 84). Additionally, we changed the term "neuroprotective genetic profile' to 'expression of neuroprotective factors' (line 338). As mentioned before, we have avoided designating A1 astrocytes as a universal neurotoxic phenotype and have rewritten a more balanced discussion, specifically citing newly published research on neuroinflammatory astrocyte subtypes, which was not yet published at the time of the initial submission of our manuscript[2,3]. We must note that after a discussion with the NanoString expert team, we were advised to stay consistent with the annotations produced and developed by their complex algorithms. Nevertheless, we have also incorporated a statement acknowledging the limitation of the A1/A2 dichotomy provided by the NanoString panel in the Result section (lines 327 to 330). Finally, based on the reviewer's comment, we decided to rephrase the statement "clarify the role of astrocytic tau in known neuronal networks effects of tau reduction in health and disease", to "provided evidence for the possible role of astrocytic-tau in the known neuronal effects of tau reduction on health and disease". This is a concept that has been described in detail in the context of tau downregulation [1].

2. Model and experimental design.
Importantly, the presence of tau in astrocytes is not convincingly demonstrated. Supplementary Fig. 1 does not show a significant expression of tau in WT astrocytes nor a reduction in tau -/- astrocytes.

As indicated before, we have improved our western blots showing the presence of tau in WT astrocytes (Supplementary Figure 1 B and Figure 7 B). To avoid confusion, it is of particular importance to clarify that Supplementary Figure 1 B shows tau expression in WT astrocytes with and without Aβ oligomer treatment, and not $tau^{-/-}$ astrocytes as stated by the reviewer.

There is no tau immunohistochemistry in Fig. 4D comparing cortices from control and tau-/- mice.

The goal for figure 4D (figure 5 in the revised version) was to evaluate the levels of PTX3 in $tau^{-/-}$ brain samples versus WT controls, not to confirm that tau is not expressed in $Tau^{-/-}$ mice. As mentioned before, it has been demonstrated multiple times that the Tau KO mouse model used in this study does not express tau.

The Western blot in Fig. Fig. 6B showing the effect of silencing with shRNATau shows a remarkable expression in WT astrocytes unlike the one in Supplementary Fig. 1, when the expression of tau in control conditions should be similar—unless infection with a viral vector up-regulates tau in astrocytes. And contrary to the description in the text, there is indeed increased tau fluorescence in Abeta-treated astrocytes. Comparisons with neurons are in order.

We have included new western blots that appropriately demonstrate the presence of tau in WT astrocytes and its decreased levels after the AAV infection (Supplementary Figure 1 B and Figure 7 B). We were confused by the statement expressed by the reviewer ("*there is indeed increased tau fluorescence in Abeta-treated astrocytes*") since there are no tau immunofluorescence experiments in our study. We decided to remove Supplementary Figure 1 A, which originally showed GFAP staining, to avoid any misunderstandings.

Fields at lower magnification should be shown to prove that PTX3 is increased in all cortical astrocytes. Fluorescence quantification should be described in more detail (number areas analyzed, magnification, number of astrocytes per field, etc.). The same for synaptic markers.

As the reviewer suggested, we have included lower magnification photographs (20X and 60X) of the PTX3 positive staining in the brain slides (Figure 5) and expanded our previous results by assessing the expression of PTX3 in cortical astrocytes. As stated in the Methods section, we improved our colocalization analysis by using the Pearson correlation coefficient as a colocalization index[9]. Additionally, we added a detailed explanation of the n breakdown for this set of experiments (Line 242 to 268).

Although it is stated in the manuscript that 'The data that support the findings of this study are available in the supplementary material of this article', raw data cannot be found. It might be helpful to provide some of the raw materials that were used to produce data, such as western blots from independent cultures or manipulations, or the whole set of images used in fluorescent analyses.

We included a data availability statement in our manuscript in the Methods section. Uncropped western blot images have been included as supplementary figure 3. NanoString raw data have been uploaded to NCBI's Gene Expression Omnibus (GEO).

Justify the choice of 10 ug of ACM to treat neuronal cultures. Were different ACM and doses tested? A missing control is the synaptic density in the absence of any ACM. It is probable that ACM from control astrocytes increase synaptic density in neuronal cultures, such that the reduction of synapses in the presence of Abeta/ACM represents a loss rather than a gain of toxic function mediated by astrocytic tau.

We thank the reviewer for these comments. In the initial exploratory experiments of our study, 10 μg of ACM was found to be the lowest dose that caused changes in the number of synaptic clusters without alterations in the neuronal culture viability. As mentioned before, we included the control suggested by the reviewer. Please refer to Figure 1, 2, 6 and 8 (gray bars). The addition of this control (synaptic density in the absence of any ACM), confirmed that the synapse in the presence of Abeta/ACM represents a gain of toxic function, as the reviewer indicated.

Justify the use of the Mouse Glial Profiling Panel. Glial cells are highly distinct, there is no such thing as a 'glia' marker. Perhaps only astrocytic genes should be used in the analysis.

As stated by the manufacturer, the Nanostring Mouse Glia Profiling Panel covers 770 genes across 50+ pathways involved in glial cell biology. These pathways include "Cell stress & Damage Response", "Pathway regulating glia", "Inflammation & Peripheral Immune Invasion", "Glial Cell Homeostasis & Activation" and "Neurotransmission". It is not stated in the manuscript that these 770 genes are "glial" markers *per se* since it is well known that many of these genes are also expressed in other cell types, such as neurons. In the manuscript, we stated that "the NanoString Technologies glial profiling panel evaluates the expression levels of 770 genes involved in glial cell biology".

Justify the dose used of PTX3 in reversal experiments. Was blockade of endogenous PTX3 tested?

We chose this PTX3 concentration following the recently published study by Fossati *et al.*[10] where it was shown that 1 μg/mL of PTX3 is sufficient to induce synaptic establishment and function when added to neurons *in vitro*. We did not test endogenous blockage of PTX3.

3. Statistical analysis.
Whether cultures or individual neurons are considered as independent observations in the means +/+ SD should be clarified; if it is single neurons, the variability among experiments with different cultures should be depicted. Likewise, as noted, how many batches of ACM were used from independent astrocyte cultures and treatments with Abeta? Indeed, the molecular analysis in Fig. 3D shows that differences among different batches of tau null astrocytes are not negligible.

We agree with the reviewer about these concerns. As mentioned before, we have expanded our Methods section and added a detailed explanation of the n breakdown for each of the experiments in our study as follows: The n breakdown for each set of experiments is as follows. *Synaptic quantification (Figures 1, 2, 6 & 8)*: we established 4 independent neuronal cultures from individual littermates (n = 4). Ten photographs of each culture per condition were randomly taken across the coverslip, depicting 1 neuron. A single neurite was randomly chosen, analyzed and quantified for synaptic markers per image. The final n was obtained by averaging the 10 quantifications of each marker and their colocalization. For *tau western blot, tau qPCR (Supplementary Figure 1A-C), differential gene expression analysis (Figure 3 and Supplementary Figure 2), astrocyte-derived PTX3 concentration (Figure 4C), AAV-infection and generation of astrocyte conditioned media (ACM) (Fig 7)*, we performed 3 independent astrocyte cultures for each condition (n = 3), established from 3 independent littermates. For *GFAP/PTX3 co-staining in vitro (Figure 4A & B and Figure 7C & D)*, we performed 3 independent astrocyte cultures (n = 3). We prepared 3 technical replicates with each culture. Five pictures per replicate were randomly taken across the coverslip and quantified. We obtained a "technical n" by averaging the data obtained from these 5 photographs, and to obtain the final n, we averaged the corresponding 3 technical n values. For *GFAP/PTX3 co-staining in vivo (Fig. 5)*, we analyzed the brains of 6 different mice. A total of 4 photographs per mouse were taken randomly across the hippocampi and cortexes. The final n was obtained by averaging the quantification of the colocalization of both markers in those 4 photos. For the *MTS astrocyte viability assay (Supplementary Figure 1D)*, 5

independent cultures were performed. A total of 2 technical replicates were performed per culture and averaged to obtain the final n. Statistical analyses were performed using the GraphPad Prism software. Normal distribution of data was evaluated using a Shapiro–Wilk Normality Test; subsequently, statistical comparisons and p-value calculations were conducted using one-way ANOVA and two-tailed unpaired Student's t-tests as stated for each experiment. Alternatively, the Kruskal–Wallis test was used when this criterion was not met. Significance was set at $p<0.05$. Statistically significant P values are shown in the figures. Data are presented as the mean ± SEM.

4. Limitations.
Whether the model truly mimics ARTAG should be discussed, as well as the caveats and possible artifacts in ACM experiments.

As mentioned above, it was not the goal of this study to mimic ARTAG. The aim of our study was to determine the synaptoprotective effects of astrocytic tau downregulation in the presence of pathological stimuli rather than to evaluate the effect of astrocytic tau aggregation, which are two distinct potential tau-related pathomechanisms. Numerous studies have shown that reducing tau expression prevents or diminishes Aβ-induced neurotoxicity *in vitro* and *in vivo*. Additionally, it has recently been suggested that physiological tau functions can enable brain disease by allowing other factors and processes to alter signaling pathways[1]. We have updated the introduction (lines 64 to 70) and discussion (lines 401 to 415) to better clarify the goals of the present study. For further reading, please refer to Dr. Lennart Mucke's work described in [1]

5. Style and presentation.
In the Abstract, explain better why tau pathology in astrocytes deserves attention.

Following the reviewer's suggestion, we clarified this point in the abstract.

The size of text in all figs should be increased. Many figs could be combined, and only data for one synaptic marker may be shown and the rest may be moved to supplementary data, because the data with the two synaptic markers and the merge are almost identical.

We thank the reviewer for these constructive suggestions. However, using two distinct pre-and post-synaptic markers provides the advantage of simultaneously evaluating them individually and in proximity to each other since the colocalization is a direct measure of a conformed synapse. Assessing only a single synaptic marker does not define its true synaptic position, leaving interesting effects unevaluated. As the reviewer suggested, we have increased the size of the text in all figures.

The article is clearly written but there is room for improvement. For example, there are too many double noun modifiers, e.g. 'Astrocytic tau reduction' (title), 'Astrocytic tau deletion', better 'deletion and reduction of astrocytic tau'; 'neuronal tau absence' line 224 should be 'absence of tau in neurons'; 'primary culture experiments' should be 'experiments in primary cultures'; specify brain region and species; 'cortices' is a better choice than 'cortexes'; spell out CMF-HBSS (line 91); the authors probably mean that 'tissue was disaggregated' and not 'desegregated', as though there were racial conflicts among tissue elements; 'astrocyte and neuronal culture immunofluorescence' may be changed to 'immunofluorescence studies in

astrocyte and neuronal cultures', to cite a few changes.

As the reviewer suggested, we have improved the manuscript with the assistance of a scientific editor.

Finally, as a constructive criticism, I would like to bring to your attention that it is not appropriate to make jokes about somebody's mistake during the manuscript review process. Every author of this manuscript is a member of an underrepresented minority group in the biomedical and science research environment. In some cases, English is their second language. Your comment/joke ("*desegregated', as though there were racial conflicts among tissue elements*") was not well received by my trainees. As mentors, we have an obligation to our trainees, which involves guiding them on a professional path of mutual respect, independent of the position one holds.

<u>Reviewer 2</u>

Comments to the Author

This is an elegant study that shows a potential role of astrocytic tau in abeta mediated synaptotoxicity. The main caveat of the study is the use of high levels Abeta to stimulate the cells. We know that levels of Abeta in AD brains do not reach these levels, so it compromises the relevance of the findings.

We thank the reviewer and agree with this concern. We decided to use 1 μM of Aβ to treat astrocytes based on previous studies that used a broad range of Aβ concentrations to stimulate astrocytes *in vitro* (0.1 μM to 25 μM)[11-18]. One of these studies demonstrated that concentrations above 1.5 μM of Aβ can induce apoptosis in astrocytes *in vitro* [18], and Kawano *et al*. showed that astrocytes previously treated with 0.5 μM of Aβ failed to promote synaptic function[17]. Therefore, we decided to stimulate astrocytes with 1 μM of Aβ for our experiments.
However, we acknowledge in the manuscript that this concentration is higher than physiological Aβ levels (lines 144 to 145).

I think the interpretation of the results would improve if the authors showed that there is not toxicity after treatment of astrocytes with abeta and that the effects seen are the consequence of unspecific cell death. The same with toxicity in neurons treated with ACM.

As the reviewer suggested we performed viability assays in astrocytes (WT and $Tau^{-/-}$) treated with Aβ (Supplementary Fig. 1D). The results demonstrated that Aβ-treatment did not affect cell viability in either WT or $tau^{-/-}$ astrocyte cultures (Supplementary Fig. 1 D), suggesting that the astrocyte-mediated synaptotoxicity under Aβ stimulation could be due to active secretion of toxic factors rather than nonspecific astrocytic death.

Measuring astrocytic reactivity by GFAP expression in vitro is not the best approach and it seems like there is no change in the morphology of the cells after adding Abeta.

We thank and agree with the reviewer on this point. Considering that this figure does not add value to the manuscript, we have removed it and the associated discussion.

The authors claim that tau-/- astrocytes acquire a more A2 phenotype, but (without taking into account that A1/A2 dichotomy might be a bit controversial) they don't show A1 phenotype, it could be that they also show a less A1 phenotype?

We thank the reviewer for mentioning this point. Reviewer 1 expressed similar concerns regarding the A1/A2 controversy. Therefore, we have avoided designating A1 astrocytes as a universal neurotoxic phenotype and stating that the $tau^{-/-}$ astrocytes have less of an A1 profile. We have rewritten a more balanced discussion (lines 426 to 436), specifically citing newly published research on neuroinflammatory astrocyte subtypes, which was not yet published at the time of the initial submission[2,3]. We have also incorporated a statement acknowledging the limitation of the A1/A2 dichotomy provided by the NanoString panel in the results section (lines 327 to 330).

The authors do not mention the concentration of PTX3 used for the experiments, is it similar to the concentration found in Tau-/- astrocytes?

We thank the reviewer for this observation. The concentration of PTX3 used in our experiments is now included in the Methods section and the legend of Figure 6 (1 ug/mL). As explained in response to Reviewer 1, we chose this PTX3 concentration following the recently published study by Fossati *et al.*[10], in which it was shown that 1 μg/mL of PTX3 is sufficient to induce synaptic establishment and function when added to neurons *in vitro*. We acknowledge that this concentration is higher than the concentration found in ACM. This caveat of the study is discussed in this revised version (lines 459 to 464). We mentioned that "our data suggest that a mechanism involving PTX3 could be responsible of the synaptoprotective effect of astrocytic tau ablation. In the present study, we demonstrated that the addition of recombinant active PTX3 to cellular media was synaptoprotective (Figure 6). Nevertheless, the concentration of recombinant active PTX3 was higher than the level of endogenous PTX3 secreted by $tau^{-/-}$ astrocytes (Figure 4). Therefore, considering that other neuroprotective factors are upregulated in $tau^{-/-}$ astrocytes (Figure 3 and Supplementary Figure 2), we cannot rule out that other factors could also play a synaptoprotective role in the context of astrocytic tau downregulation."

I think the authors could explain a bit better what is the potential link between tau and PTX3

We thank the reviewer's suggestion. The central idea of our study is that the physiological function of tau in astrocytes could be associated with a signaling pathway responsible for the differential PTX3 expression and secretion. We have included this concept in our Discussion section (line 465 to 470).

<u>Comments from the Scientific Editor</u>

Please, provide details of the experimental unit (n= mice, cells, wells, plate downs, etc)) for each group analysed in these plots, and if the number is <10 show the single data-points. Please note our policy on pseudoreplication, we do not accept analyses where individual cells are the experimental unit when there is likely biological variability is between animals, culture replicates, etc. Please see instructions for authors for more details. Fort this type of data, we require either that a mean/median be taken per animal/culture replicate followed by statistical analyses or that more sophisticated analyses are performed such as mixed effects modelling which can account for random variables.

We thank the Editor for these suggestions. We have expanded our Methods section (line 242 to 268) and added a detailed explanation of the n breakdown for each of the experiments in our study. We have include the single data points for each quantification.

Please, add the scale bar to Supp Fig1a.

Supplementary Figure 1A has been excluded from this revision.

Please, provide Full- size and uncropped blots/gels as Supplementary material.

We have added all uncropped blots to Supplementary Figure 3.

Please supply a graphical abstract. A graphical abstract is a visual representation of the central finding or methodology of the paper. It will be displayed in the table of contents. Authors should strive to make the graphical abstract informative, interesting, visually appealing, and straightforward (no caption needed, no abbreviations except gene names). Please upload as a separate high resolution image file (tiff preferred). The technical requirements for the graphical abstracts are:

- *size of the submitted image: 1200 pixels square at 300dpi*
- *font of the text: Arial 12-16 point (smaller fonts will not be legible online)*

A graphical abstract has been included in the revised version.

Please add an Abbreviated Summary (up to 50 words) of your paper that captures the main purpose and conclusions of the work. The summary should state the main results written in the third person (‘[1st author surname] et al. report that ...’) and conclusion. This summary will be used in the contents list online and should not contain any abbreviations other than those on the accepted abbreviations list and accepted gene/protein names, making it accessible to the non-specialist. Please upload it as a Word document. Allowed abbreviations: AIDS; ANOVA; AMPA; ATP; A,T,C,G; CNS; CSF; CT; DNA; ECG; EEG; EMG; GABA; HIV; MRI; NMDA; PET; PCR; RNA.

An abbreviated summary has been included in the revised version.

To comply with our ethical standards, the appropriate checklist must be included (see Reporting Guideline section within 'Instructions to authors').

We have stated in the manuscript that all work involving mice was performed following the guidelines of the Institutional Animal Care and Use Committee and in compliance with the Animal Welfare Act, the Guide for the Care and Use of Laboratory Animals, the Office of Laboratory Animal Welfare and the guidelines of Indiana University School of Medicine.

Please include a paragraph about data availability in the 'Materials and Methods' section. We strongly encourage data sharing either as supplementary information or uploads to repositories which you can link in the paper.

Data availability has been included in the methods sections. NanoString data have also been uploaded to the NCBI's Gene Expression Omnibus (GEO) (line 269 to 273).

Addition of co-author to revised manuscript

Dr. Nur Jury was included as a co-author in the revised version of this manuscript. Dr. Jury assisted with the viability assay requested by reviewer 2. She was involved in the analysis of new data and the re-analysis of existing data after the addition of new control groups. She also prepared figures and wrote the revised version of the manuscript.

References

1 Chang, C. W., Shao, E. & Mucke, L. Tau: Enabler of diverse brain disorders and target of rapidly evolving therapeutic strategies. *Science* **371**, doi:10.1126/science.abb8255 (2021).

2 Escartin, C. *et al.* Reactive astrocyte nomenclature, definitions, and future directions. *Nature neuroscience* **24**, 312-325, doi:10.1038/s41593-020-00783-4 (2021).

3 Hasel, P., Rose, I. V. L., Sadick, J. S., Kim, R. D. & Liddelow, S. A. Neuroinflammatory astrocyte subtypes in the mouse brain. *Nature Neuroscience* **24**, 1475-1487, doi:10.1038/s41593-021-00905-6 (2021).

4 Garwood, C. J., Pooler, A. M., Atherton, J., Hanger, D. P. & Noble, W. Astrocytes are important mediators of Abeta-induced neurotoxicity and tau phosphorylation in primary culture. *Cell death & disease* **2**, e167, doi:10.1038/cddis.2011.50 (2011).

5 Wasilewski, D. *et al.* Reactive Astrocytes Contribute to Alzheimer's Disease-Related Neurotoxicity and Synaptotoxicity in a Neuron-Astrocyte Co-culture Assay. *Frontiers in cellular neuroscience* **15**, 739411, doi:10.3389/fncel.2021.739411 (2021).

6 Lines, J. *et al.* Astrocyte-neuronal network interplay is disrupted in Alzheimer's disease mice. *Glia* **70**, 368-378, doi:10.1002/glia.24112 (2022).

7 Kaech, S. & Banker, G. Culturing hippocampal neurons. *Nature protocols* **1**, 2406-2415, doi:10.1038/nprot.2006.356 (2006).

8 Beaudoin, G. M., 3rd *et al.* Culturing pyramidal neurons from the early postnatal mouse hippocampus and cortex. *Nature protocols* **7**, 1741-1754, doi:10.1038/nprot.2012.099 (2012).

9 Bolte, S. & Cordelieres, F. P. A guided tour into subcellular colocalization analysis in light microscopy. *J Microsc* **224**, 213-232, doi:10.1111/j.1365-2818.2006.01706.x (2006).

10 Fossati, G. *et al.* Pentraxin 3 regulates synaptic function by inducing AMPA receptor clustering via ECM remodeling and beta1-integrin. *The EMBO journal* **38**, doi:10.15252/embj.201899529 (2019).

11 Hu, J., Akama, K. T., Krafft, G. A., Chromy, B. A. & Van Eldik, L. J. Amyloid-beta peptide activates cultured astrocytes: morphological alterations, cytokine induction and nitric oxide release. *Brain research* **785**, 195-206 (1998).

12 Chiarini, A., Armato, U., Gardenal, E., Gui, L. & Dal Pra, I. Amyloid beta-Exposed Human Astrocytes Overproduce Phospho-Tau and Overrelease It within Exosomes, Effects Suppressed by Calcilytic NPS 2143-Further Implications for Alzheimer's Therapy. *Frontiers in neuroscience* **11**, 217, doi:10.3389/fnins.2017.00217 (2017).

13 Abdullah, M. *et al.* Amyloid-beta Reduces Exosome Release from Astrocytes by Enhancing JNK Phosphorylation. *Journal of Alzheimer's disease : JAD* **53**, 1433-1441, doi:10.3233/JAD-160292 (2016).

14 Wyssenbach, A. *et al.* Amyloid beta-induced astrogliosis is mediated by beta1-integrin via NADPH oxidase 2 in Alzheimer's disease. *Aging cell* **15**, 1140-1152, doi:10.1111/acel.12521 (2016).

15 Rama Rao, K. V., Curtis, K. M., Johnstone, J. T. & Norenberg, M. D. Amyloid-beta inhibits thrombospondin 1 release from cultured astrocytes: effects on synaptic protein expression. *Journal of neuropathology and experimental neurology* **72**, 735-744, doi:10.1097/NEN.0b013e31829bd082 (2013).

16 Allaman, I. *et al.* Amyloid-beta aggregates cause alterations of astrocytic metabolic phenotype: impact on neuronal viability. *The Journal of neuroscience : the official journal of the Society for Neuroscience* **30**, 3326-3338, doi:10.1523/JNEUROSCI.5098-09.2010 (2010).

17 Kawano, H. *et al.* Astrocytes with previous chronic exposure to amyloid beta-peptide fragment 1-40 suppress excitatory synaptic transmission. *Journal of neurochemistry* **143**, 624-634, doi:10.1111/jnc.14247 (2017).

18 Saha, P. & Biswas, S. C. Amyloid-beta induced astrocytosis and astrocyte death: Implication of FoxO3a-Bim-caspase3 death signaling. *Molecular and cellular neurosciences* **68**, 203-211, doi:10.1016/j.mcn.2015.08.002 (2015).
