# Supplementary material for: The reduction of astrocytic tau prevents amyloid-β-induced synaptotoxicity
Source: Brain Commun. 2022 Sep 19;4(5):fcac235. doi: 10.1093/braincomms/fcac235 (PMC9527666; doi:10.1093/braincomms/fcac235)
Supplement: fcac235_Supplementary_Data [file Response_to_reviewers_comments_revision_1.pdf › Response_to_reviewers_comments_revision_1.pdf]

BRAINCOM-2022-015.R1: The Reduction of Astrocytic Tau Prevents Aβ-Induced Synaptotoxicity.

Response to Reviewers and Editor

We would like to thank the reviewers for their constructive suggestions, which helped us improve our revised manuscript. Our responses to specific comments are below. Original reviewer comments are formatted in *italics* followed by our answers in regular font. All new additions to the study based on the reviewer's comments are in blue font in the revised manuscript.

<u>Reviewer Comments</u>

<u>Reviewer 1</u>

Many of the critiques were addressed but important issues remain concerning the model, the analysis, the use of the A1/A2 framework and the writing.

We believe we have adequately addressed all issues regarding the A1/A2 astrocyte terminology. We clearly stated in the revised version that astrocyte states are not binary. Nevertheless, in the revised version, we mentioned the A1/A2 terminology when we discussed previous data that classified astrocytes in that manner.
The manuscript has been revised three times by a scientific editor (Dr. Louise Pay). All editorial suggestions from reviewer 1 have been included in this final version.

1-Whether this is a case of aging-related tau-mediated astrocytopathy (ARTAG)
The authors explain 'The aim of our study was to determine the synaptoprotective effects of astrocytic tau downregulation under pathological stimuli rather than evaluate the effect of astrocytic tau aggregation, which are two distinct potential tau-related pathomechanisms.' This is a fair argument. Still, it may benefit the study to frame it as an ARTAG scenario. <u>Arguments</u>:

<u>First</u>, ARTAG refers to spectrum of astrocytic pathology detected by tau immunohistochemistry, particularly phosphorylation-dependent, 4R isoform-specific antibodies and tangles. In the present study, there is no analysis of the impact of Abeta on tau phosphorylation or cleavage in astrocytes. Thus, it cannot be ruled out that toxic tau species are generated in astrocytes upon Abeta exposure. Moreover, the authors seem to implicitly assume that this may be the case, for the refs they use as rationale for the study are cases of ARTAG (refs 19, 38), and they argue that reducing endogenous tau would bypass the need to determine which forms of tau are most detrimental (Line 72).
<u>Second</u>, the natural readership of this study is researchers with an interest in ARTAG; they will thus wonder whether this is ARTAG. In my opinion, it is not farfetched to propose that ARTAG encompasses a wide spectrum of tau-derived pathologies caused by many forms of tau, including physiological tau and all the spectrum of tau aggregates from oligomeric Tau to tangles. This idea indeed aligns well with the spirit of the piece by Dr. Mucke in ref 6.

We thank the reviewer for these valuable arguments. Considering the concerns of reviewer 1 regarding the effect of Aβ on astrocytic tau phosphorylation and cleavage, we included the

subsequent statement in the results section "Nevertheless, we cannot rule out that Aβ treatment affected tau phosphorylation or cleavage that is not detected by the total tau antibody utilized in this study".

Along these lines, the issue of whether astrocytes express tau is highly relevant. Ref 16 in line 76 is wrong, as this study does not show that astrocytes express tau; and ref 13 (brainseq) shows that human astrocytes express 10 times less tau than neurons. This should be stated and discussed in more length because reasonable doubts may arise as to the relevance of basal astrocytic tau in Alzheimer's disease given these numbers. Line 80 is misleading, because it is not that the expression of tau in astrocytes in AD is low, but that its basal expression is low.

We thank the reviewer for pointing out the mistake regarding reference 16. We have updated the references.

As the reviewer suggested, we have included the following statement: "Under basal conditions, tau expression is 5 and 10 times lower in astrocytes than in neurons in humans and mice, respectively".

It is not our intention to provide misleading statements. Therefore, we have rephrased the sentence "As the expression levels of astrocytic tau in AD and related dementias are low, its relevance has yet to be studied in detail" as follows: "As the expression levels of astrocytic tau are low, its relevance has yet to be studied in detail".

Now the authors present proof of the expression of tau in primary astrocytes from WT mice by Western Blots. It is a pity that there is no immunostaining in mouse brains showing presence of tau in astrocytes and reduction thereof in tau null brains. The fact that tau is removed in all cell types does not prove that there is a physiologically relevant amount of tau in astrocytes, it just means that the genetic deletion was effective. Nevertheless, the main argument the authors can present is that depletion of tau in astrocytes, regardless of its levels of expression, has consequences.

We completely agree with the reviewer. The main argument in this study is that depletion of tau in astrocytes, regardless of its levels of expression, has consequences. We have not claimed in our study that physiological levels of tau in astrocytes are as relevant as in neurons.

We did not perform immunostaining in WT mouse brains to demonstrate the presence of tau in astrocytes. It not possible by immunostaining to rule out that the tau detected in astrocytes is indeed neuronal tau that has been internalized by astrocytes.

Lines 401-415. It is assumed that the relevant tau in these studies is physiological tau. Was it proven that there was no abnormal phosphorylation of tau and/or oligomeric aggregates?

Yes, in all these studies (ref 9,64,65,66 and 67), it was proven that endogenous murine tau is not abnormally phosphorylated and does not form oligomeric aggregates.

3-Statistics and methodology
--In the previous submission, it was specified in the figure legends that data were expressed as the means +/- SD. Now this is gone, and SEM is mentioned in Methods, as in the previous version. Please, specify in the figure legends whether the variability is expressed as SD or SEM.

The information is included in each figure legend and in the Materials and Methods section. All results are expressed as SEM.

--Line 18. Correct that GFAP was not used to assess purity but to determine whether PTX3 was expressed in astrocytes. To assess purity, antibodies against microglia and oligodendrocyte progenitors should have been used. The authors do not need to do these experiments now, since they were not asked in the first round, and because it is very difficult to remove microglia from cultures, such that one should assume that a proportion of cells are microglia. Arguably, this makes the cultures a better model of the in vivo interactions between astrocytes and microglia.

We appreciate the reviewer's valuable comment. We will keep this in mind for future studies.

--Lines 34 and 19. In addition to a reference to a methodological article, the image processing and variables used when quantifying immunofluorescence should be specified in more detail in Methods. For instance, PTX3 immunohistochemistry has a high background. How was it dealt with? Was the quantification automatic or manual?

As the reviewer suggested, we added more detail about immunofluorescence quantification to the methods section.

Suggestions for editing the language:

We thank the reviewer for his editorial suggestions. Before every submission the manuscript has been proofread and edited by our Institute scientific editor, Dr. Louise Pay.

--Line 34: 'We performed a series of experiments' is too vague. Please, succinctly describe the models in the abstract.
As the reviewer suggested, we change this sentence to "we performed a series of astrocytic and neuronal primary cultures".

--Line 37: the sentence is unclear since the model is not explained earlier. Particularly, in 'their exposure', the subject of 'their' (synapses or astrocytes) needs to be clarified.
As the reviewer requested in the previous observation, we explained the model in line 34. The explanation of the model in line 34 clarified the statement in line 37.

--Line 64: 'enable' is not the right word here and in the rest of the text. For two reasons. First, even though Dr. Mucke uses it with physiological tau in the sense of 'making pathology possible' in his article in ref 6, it is only one scenario among others with regards to the mechanisms

whereby tau contributes to pathology. As noted above, astrocytic tau may be aberrantly phosphorylated and aggregated in highly toxic oligomers in astrocytes exposed to amyloid-beta. That is, there is no reason to assume that it is the physiological tau that promotes the phenotypic transformation of astrocytes—or mediates the reduction of pathology in refs 7-12 observed upon reduction of tau expression, for, as the authors argue, the reduction of tau would affect any tau-derived species. Second, 'enable' is normally used with a positive connotation such that 'to enable diseases' is an oxymoron. Better alternatives: mediate, lead to, contribute, play a role, etc.
As the reviewer suggested, we changed "enable" to "contribute to"

--*Line 65: 'other factors' 'other signaling pathways' are too unprecise.*
We thank the reviewer for this comment. The signaling pathways involved in this process have not been identified in detail. Therefore, we decided to keep the term "other factors" considering that these factors have not been identified with precision.

--*Line 66: the sentence makes no sense; it should be the adverse effects of Abeta on neuronal plasticity and cognition or Abeta-mediated neurodegeneration and cognitive deficits.*
As the reviewer suggested, we have changed the sentence to "<u>Aβ-mediated neurodegeneration and cognitive deficits</u>".

--*Line 43: 'to manage the deleterious effects of synaptic damage in AD' points to therapies such as memory enhancers. Probably the authors mean 'to prevent synaptic damage in AD'.*
As the reviewer suggested, we changed the sentence to "<u>to prevent synaptic damage in AD</u>".

--*Line 82. Give some details of the model to study the interaction between astrocytes and neurons to provide a link with line 84.*
Due to space constrictions, we cannot further expand the introduction and discussion. Nevertheless, we provided references for the statements in lines 82 and 84.

--*Line 246: 'depicting' is not the right word.*
Based on the reviewer's comments, we decided to delete '<u>depicting 1 neuron</u>' from the phrase in line 246.

--*Line 279: For the sake of precision, the statement 'Additionally, it has been proposed that astrocytes could be active mediators of A β −induced neurotoxicity' can be completed with 'by exacerbating Tau cleavage by caspase-3' (ref 37).*
As the reviewer suggested, this statement has been included in the manuscript.

--*Line 280: Perhaps a better way to describe the experiment is: We first aimed to confirm this study (ref 37) by determining whether depletion of neuronal tau prevented the synaptotoxic effects of Ab-stimulated astrocytes.*
We respectfully disagree with the reviewer's observation. Our goal was never to confirm the study described in ref 37. Our first goal was to determine if the absence of tau in neurons confers protection against the synaptotoxic effects of Aβ-stimulated astrocytes independent of the mechanism. Adding this statement could be misleading.

--*Line 282: remove 'a series of experiments', too vague.*
As the reviewer suggested, we changed the sentences to 'a series of astrocytic and neuronal primary cultures.'

--*Line 285: 'as a control' or 'as controls' but not 'as a controls'.*
We thank the reviewer for this observation. We changed the sentence to 'As a control'.

--*Line 289: 'avoided' is not the right word.*
We changed the word 'avoided' to 'prevented'

--*Line 292: 'Ab-induced' astrocytes, 'induced' is not the right word.*
We changed the word 'induced' to 'stimulated.'

--*Line 302: In comparison 'to' control neurons, not 'to in' control neurons.*
We thank the reviewer for this observation. The word 'in' was deleted from the sentence.

--*Line 303: Quantification 'in' not 'on'.*
We changed the word 'on' to 'in'.

--*Line 309: rather than 'to' non-specific astrocyte death.*
The word 'to' has been included in the sentence.

Reviewer 2

The authors have done a great job answering all the comments raised by the other reviewer and myself. I think the manuscript has improved a lot, I still think that the concentrations of ABeta are too high and somehow compromise the relevance of the findings, but overall the experimental design, results and interpretation of the data are well performed.

We thank the reviewer for the valuable comments. To mitigate the reviewer's concern regarding the concentration of Aβ utilized in this study, we have included the following statement in the conclusion: 'Considering that the current study was performed in astrocytic primary cultures stimulated with a supraphysiological concentration of Aβ, further *in vivo* studies are necessary to validate the observations.'
