# Supplementary material for: The reduction of astrocytic tau prevents amyloid-β-induced synaptotoxicity
Source: Brain Commun. 2022 Sep 19;4(5):fcac235. doi: 10.1093/braincomms/fcac235 (PMC9527666; doi:10.1093/braincomms/fcac235)
Supplement: fcac235_Supplementary_Data [file Revision_1_decision_letter.pdf › Revision_1_decision_letter.pdf]

Subject: Brain Communications - Decision on Manuscript ID BRAINCOM-2022-015.R1

Body: 08-Jul-2022

Dear Prof. Lasagna-Reeves,

Thank you for your recent submission BRAINCOM-2022-015.R1
entitled "THE REDUCTION OF ASTROCYTIC TAU PREVENTS Aβ-INDUCED SYNAPTOTOXICITY" to Brain Communications.

Your manuscript has been reviewed, and while the reviewers had largely positive comments, they have some constructive critiques that I think you could address in a minor revision of the manuscript. Therefore, I invite you to respond to the reviewer(s)' comments and revise your manuscript. I agree with reviewer 2 that 1 micromolar Abeta is a supraphysiological concentration. Citiing previous studies using this is fine but please also include a clear statement about the limitations of using high concentrations of Abeta in the paper. Reviewer 1 raised points about A1 vs A2 terminology which have been adequately addressed in the revision in my view. While it is clear that astrocyte states are not binary, it is fine to refer to previous data that classified them in that way. I have forwarded reviewer 1's full comments in a separate message. Below find the shortened version with their comments that you may wish to consider in the revision.

Please format your revised manuscript according to our Instructions to Authors https://academic.oup.com/braincomms/pages/General_Instructions – a summary of key points is attached to help with this.

To revise your manuscript, click the link below or log into https://mc.manuscriptcentral.com/braincom and enter your Author Centre, where you will find your manuscript title listed under "Manuscripts with Decisions." Under "Actions," click on "Create a Revision." Your manuscript number will be appended to denote a new revision.

*** PLEASE NOTE: This is a two-step process. After clicking on the link, you will be directed to a webpage to confirm. ***

https://mc.manuscriptcentral.com/braincom?URL_MASK=3caf0e03f77f44efacd0ab60bb466b7a

Please upload a revised manuscript with changes tracked using track changes or in coloured text.

When submitting your revised manuscript, you will be asked to respond to the reviewer(s) comments. Please be as specific as possible in your response to all of the comments.

The editorial team at Brain Communications also aims to increase transparency in scientific publishing and to demonstrate the value that peer review brings to the scientific process. Please therefore consider whether you are willing to have reviewer comments on your paper and your response published as supplementary data with the paper if it is accepted. This is entirely optional and will only be done if both the authors and reviewers agree. When submitting your revision you will have the opportunity to update the answer you entered previously.

IMPORTANT: Your original files are available to you when you upload your revised manuscript. Please delete any redundant files before completing the submission.

Because we are trying to facilitate timely publication of manuscripts submitted to Brain Communications, we would appreciate your revised manuscript as soon as possible. If it is not possible for you to resubmit within 3 months, the option to submit a revision will expire, and we would then consider your revised paper as a new submission. However, if you need a little more time, please get in touch and we can extend this deadline for you.

Once again, thank you for submitting your manuscript to Brain Communications and I look forward to receiving your revision.

Best wishes

Professor Tara Spires-Jones
Founding Editor, Brain Communications

Reviewer(s)' Comments to Author:

Reviewer: 2

Comments to the Author
The authors have done a great job answering all the comments raised by the other reviewer and myself. I think the manuscript has improved a lot, I still think that the concentrations of ABeta are too high and somehow compromise the relevance of the findings, but overall the experimental design, results and interpretation of the data are well performed.

Reviewer: 1

Comments to the Author
Many of the critiques were addressed but important issues remain concerning the model, the analysis, the use of the A1/A2 framework and the writing.

1-Whether this is a case of aging-related tau-mediated astrocytopathy (ARTAG)
The authors explain 'The aim of our study was to determine the synaptoprotective effects of astrocytic tau downregulation under pathological stimuli rather than evaluate the effect of astrocytic tau aggregation, which are two distinct potential tau-related pathomechanisms.' This is a fair argument. Still, it may benefit the study to frame it as an ARTAG scenario. Arguments:

First, ARTAG refers to spectrum of astrocytic pathology detected by tau immunohistochemistry, particularly phosphorylation-dependent, 4R isoform-specific antibodies and tangles. In the present study, there is no analysis of the impact of Abeta on tau phosphorylation or cleavage in astrocytes. Thus, it cannot be ruled out that toxic tau species are generated in astrocytes upon Abeta exposure. Moreover, the authors seem to implicitly assume that this may be the case, for the refs they use as rationale for the study are cases of ARTAG (refs 19, 38), and they argue that reducing endogenous tau would bypass the need to determine which forms of tau are most detrimental (Line 72).

Second, the natural readership of this study is researchers with an interest in ARTAG; they will thus wonder whether this is ARTAG. In my opinion, it is not farfetched to propose that ARTAG encompasses a wide spectrum of tau-derived pathologies caused by many forms of tau, including physiological tau and all the spectrum of tau aggregates from oligomeric Tau to tangles. This idea indeed aligns well with the spirit of the piece by Dr. Mucke in ref 6.

Along these lines, the issue of whether astrocytes express tau is highly relevant. Ref 16 in line 76 is wrong, as this study does not show that astrocytes express tau; and ref 13 (brainseq) shows that human astrocytes express 10 times less tau than neurons. This should be stated and discussed in more length because reasonable doubts may arise as to the relevance of basal astrocytic tau in Alzheimer's disease given these numbers. Line 80 is misleading, because it is not that the expression of tau in astrocytes in AD is low, but that its basal expression is low.

Now the authors present proof of the expression of tau in primary astrocytes from WT mice by Western Blots. It is a pity that there is no immunostaining in mouse brains showing presence of tau in astrocytes and reduction thereof in tau null brains. The fact that tau is removed in all cell types does not prove that there is a physiologically relevant amount of tau in astrocytes, it just means that the genetic deletion was effective. Nevertheless, the main argument the authors can present is that depletion of tau in astrocytes, regardless of its levels of expression, has consequences.

Lines 401-415. It is assumed that the relevant tau in these studies is physiological tau. Was it proven that there was no abnormal phosphorylation of tau and/or oligomeric aggregates?

3-Statistics and methodology

--In the previous submission, it was specified in the figure legends that data were expressed as the means +/- SD. Now this is gone, and SEM is mentioned in Methods, as in the previous version. Please, specify in the figure legends whether the variability is expressed as SD or SEM.
--Line 18. Correct that GFAP was not used to assess purity but to determine whether PTX3 was expressed in astrocytes. To assess purity, antibodies against microglia and oligodendrocyte progenitors should have been used. The authors do not need to do these experiments now, since they were not asked in the first round, and because it is very difficult to remove microglia from cultures, such that one should assume that a proportion of cells are microglia. Arguably, this makes the cultures a better model of the in vivo interactions between astrocytes and microglia.
--Lines 34 and 19. In addition to a reference to a methodological article, the image processing and variables used when quantifying immunofluorescence should be specified in more detail in Methods. For instance, PTX3 immunohistochemistry has a high background. How was it dealt with? Was the quantification automatic or manual?

Suggestions for editing the language:
--Line 34: 'We performed a series of experiments' is too vague. Please, succinctly describe the models in the abstract.
--Line 37: the sentence is unclear since the model is not explained earlier. Particularly, in 'their exposure', the subject of 'their' (synapses or astrocytes) needs to be clarified.
--Line 64: 'enable' is not the right word here and in the rest of the text. For two reasons. First, even though Dr. Mucke uses it with physiological tau in the sense of 'making pathology possible' in his article in ref 6, it is only one scenario among others with regards to the mechanisms whereby tau contributes to pathology. As noted above, astrocytic tau may be aberrantly phosphorylated and aggregated in highly toxic oligomers in astrocytes exposed to amyloid-beta. That is, there is no reason to assume that it is the physiological tau that promotes the phenotypic transformation of astrocytes—or mediates the reduction of pathology in refs 7-12 observed upon reduction of tau expression, for, as the authors argue, the reduction of tau would affect any tau-derived species. Second, 'enable' is normally used with a positive connotation such that 'to enable diseases' is an oxymoron. Better alternatives: mediate, lead to, contribute, play a role, etc.
--Line 65: 'other factors' 'other signaling pathways' are too unprecise.
--Line 66: the sentence makes no sense; it should be the adverse effects of Abeta on neuronal plasticity and cognition or Abeta-mediated neurodegeneration and cognitive deficits.
--Line 43: 'to manage the deleterious effects of synaptic damage in AD' points to therapies such as memory enhancers. Probably the authors mean 'to prevent synaptic damage in AD'.
--Line 82. Give some details of the model to study the interaction between astrocytes and neurons to provide a link with line 84.
--Line 246: 'depicting' is not the right word.
--Line 279: For the sake of precision, the statement 'Additionally, it has been proposed that astrocytes could be active mediators of A β −induced neurotoxicity' can be completed with 'by exacerbating Tau cleavage by caspase-3' (ref 37).
--Line 280: Perhaps a better way to describe the experiment is: We first aimed to confirm this study (ref 37) by determining whether depletion of neuronal tau prevented the synaptotoxic effects of Ab-stimulated astrocytes.
--Line 282: remove 'a series of experiments', too vague.
--Line 285: 'as a control' or 'as controls' but not 'as a controls'.
--Line 289: 'avoided' is not the right word.
--Line 292: 'Ab-induced' astrocytes, 'induced' is not the right word.
--Line 302: In comparison 'to' control neurons, not 'to in' control neurons.
--Line 303: Quantification 'in' not 'on'.
--Line 309: rather than 'to' non-specific astrocyte death.

Date Sent: 08-Jul-2022
